# Changes in psychotropic polypharmacy and high‐potency prescription following policy change: Findings from a large scale Japanese claims database

**DOI:** 10.1111/pcn.13432

**Published:** 2022-07-02

**Authors:** Masahiro Takeshima, Minori Enomoto, Masaya Ogasawara, Mizuki Kudo, Yu Itoh, Kazuhisa Yoshizawa, Dai Fujiwara, Yoshikazu Takaesu, Kazuo Mishima

**Affiliations:** ^1^ Department of Neuropsychiatry Akita University Graduate School of Medicine Akita Japan; ^2^ Department of Medical Technology, School of Health Sciences Tokyo University of Technology Tokyo Japan; ^3^ Department of Neuropsychiatry, Graduate School of Medicine University of the Ryukyus Okinawa Japan

Dear editor,

Psychotropic polypharmacy and long‐term benzodiazepine receptor agonist (BzRA) use are major health issues as they can increase the risk of adverse effects. To promote the appropriate use of psychotropic drugs in Japan, medical fee reductions were implemented four times between 2012 and 2018 (Table S1).^1^

This observational study aimed to evaluate the effect of medical fee revisions on the polypharmacy and prescription of high‐potency psychotropics by distinguishing between before and after intervention periods using a Japan Medical Data Center (JMDC) dataset containing all medical fee data of health insurance service subscribers aged 0–74 years (workers and their family members) (Table S2). Medical information of health insurance service subscribers who visited any department of a medical institution (hospitals, clinics, etc.) every April from 2005 to 2019 and were prescribed psychotropic drugs was extracted. Dosages of sulpiride <300 and ≥300 mg/day were considered antidepressant and antipsychotic dosages, respectively. The potency of psychotropics was calculated based on the psychotropic dose equivalence in Japan.^2^ Drugs not listed in the psychotropic dose equivalence in Japan were defined as follows: flunitrazepam 1 mg/day = suvorexant 20 mg/day = ramelteon 8 mg/day and chlorpromazine 100 mg/day = asenapine 2.5 mg/day = brexpiprazole 0.5 mg/day (Table S3). The proportion of patients prescribed three or more psychotropics—such as anxiolytics, hypnotics, antidepressants, and antipsychotics—(polypharmacy rate) among the total number of prescribed psychotropics was calculated. Subsequently, the proportions of those prescribed high‐potency psychotropics among those prescribed psychotropics (rate of prescription of high‐potency psychotropics) were calculated. High‐potency anxiolytics, hypnotics, antidepressants, and antipsychotics were defined as those equivalent to >15 mg/day diazepam, >2 mg/day flunitrazepam, >300 mg/day imipramine, and >600 mg/day chlorpromazine, respectively. The rates of polypharmacy and prescription of high‐potency psychotropics were adjusted using the Japanese annual vital statistics. We set as benchmarks a reduction in the rates of the polypharmacy and prescriptions of high‐potency psychotropics to qualify the intervention as beneficial.

This study was approved by the Ethics Committee of Akita University Graduate School of Medicine (approval number: 2352). The study was conducted per the ethical principles of the Declaration of Helsinki as revised in 1989 and the International Conference on Harmonization Guideline for Good Clinical Practice. As we analyzed an anonymized dataset, informed consent was waived.

Figure 1 shows changes in the rates of polypharmacy and prescription of high‐potency psychotropics every April from 2005 to 2019 (Tables [Link], [Link], [Link], [Link], [Link], Figs [Link], [Link], [Link], [Link], [Link]). Rates of polypharmacy of all four psychotropics were considered to have decreased. However, the polypharmacy rates of anxiolytics and hypnotics from 2015 onward and antidepressants and antipsychotics from 2017 onward remained stable. In contrast, the rates of prescription of high‐potency antipsychotics decreased, those of anxiolytics and hypnotics remained generally unchanged, and those of antidepressants increased.

**Fig. 1:**
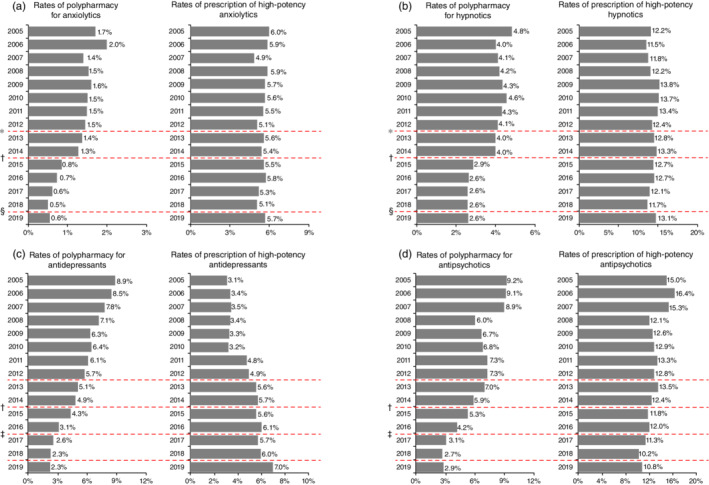
Patients prescribed three or more drugs and those prescribed high‐potency psychotropic drugs, including (a) anxiolytics, (b) hypnotics, (c) antidepressants, and (d) antipsychotics. Based on the census data, the rates of polypharmacy and prescription of high‐potency psychotropic drugs were adjusted for age based on 5‐year age groups and sex. The figure on the left shows the proportion of subscribers who were prescribed three or more of each class of psychotropic drugs among subscribers who were prescribed psychotropic drugs every April between 2005 and 2019. The figure on the right shows the proportion of subscribers prescribed high‐potency psychotropic drugs among subscribers prescribed psychotropic drugs. High‐potency anxiolytics, hypnotics, antidepressants, and antipsychotics were defined as those equivalent to >15 mg/day diazepam, >2 mg/day flunitrazepam, >300 mg/day imipramine, and >600 mg/day chlorpromazine, respectively. ^†^Revision in 2012. ^‡^Revision in 2014. ^§^Revision in 2016. ^¶^Revision in 2018.

It is difficult to reduce the prescribed dosage or discontinue the use of BzRAs, the most frequently prescribed anxiolytics and hypnotics, due to physical dependence.^3^ Patients who do not successfully reduce or discontinue BzRAs may require health policy and medical intervention. Cognitive‐behavioral therapy (CBT) may be considered in patients with anxiety disorders who cannot reduce or discontinue anxiolytic use; CBT promotes the discontinuation of both short and long‐term anxiolytic use.^4^ In contrast, CBT for insomnia facilitates the short‐term discontinuation of benzodiazepines; however, the effects may not last long.^5^ Therefore, new long‐term effective treatments for discontinuing hypnotics in patients with insomnia are required. Throughout the study period, polypharmacy rates decreased for antidepressants, whereas the rates of prescription of high‐potency psychotropics increased annually. Although guidelines recommend that antidepressants be prescribed as monotherapy when treating depression,^6^, ^7^ patients suffering from depression failed to achieve remission with antidepressant monotherapy.^8^ The increased rates of prescription of high‐potency antidepressants in this study may be due to combination therapy of antidepressants for patients who did not achieve remission with antidepressant monotherapy. Rates of polypharmacy and prescription of high‐potency antipsychotics decreased over time. Schizophrenia treatment guidelines recommend using antipsychotic monotherapy, possibly promoting appropriate antipsychotic use.^9^, ^10^

There were some limitations. First, it is unclear to what extent the JMDC dataset represents the general Japanese population. Also, this study could not exclude other societal factors, besides medical fee revisions, that might have influenced psychotropic prescribing during the study period.

In conclusion, our results indicate that medical fee revision significantly reduced the polypharmacy rates for all psychotropic drugs. However, fee revision did not reduce the prescription rates of high‐potency psychotropics other than antipsychotics. Further studies examining the effects of medical fee revisions for reducing long‐term BzRAs use are warranted.

## Disclosure statement

Masahiro Takeshima has received speaker's honoraria from Daiichi Sankyo Company, Sumitomo Dainippon Pharma, Meiji Seika Pharma, Viatris Pharmaceuticals Japan, and Yoshitomi Pharmaceutical, and research grants from Otsuka Pharmaceutical, EISAI, Shionogi and the Japanese Ministry of Health, Labour and Welfare (R3‐21GC1016) outside the submitted work. Yoshikazu Takaesu has received speaker's honoraria from Takeda Pharmaceutical, Sumitomo Dainippon Pharma, Otsuka Pharmaceutical, Meiji Seika Pharma, Kyowa Pharmaceutical, EISAI, MSD, and Yoshitomi Pharmaceutical outside the submitted work. Kazuo Mishima has received speaker's honoraria from EISAI Co., Ltd., Nobelpharma Co., Ltd., and MSD Inc., and research grants from the Japanese Ministry of Health, Labour and Welfare (19GC1012, 21GC0801) outside the submitted work. Mizuki Kudo has received speaker's honoraria from Meiji Seika Pharma outside the submitted work. Minori Enomoto, Masaya Ogasawara, Yu Itoh, Kazuhisa Yoshizawa, and Dai Fujiwara have no competing interests to declare.

## Supporting information


**Fig. S1** Trends in the rates of polypharmacy and prescription of high‐potency anxiolytics over time.

**Fig. S2** Trends in the rates of polypharmacy and prescription of high‐potency hypnotics over time.

**Fig. S3** Trends in the rates of polypharmacy and prescription of high‐potency antidepressants over time.

**Fig. S4** Trends in the rates of polypharmacy and prescription of high‐potency antipsychotics over time.

**Fig. S5** Trends in the monthly prescription rates of psychotropic drugs over time.

**Table S1** Details of the medical fee revisions to reduce psychotropic polypharmacy and long‐term use of benzodiazepine receptor agonists in Japan.

**Table S2** Demographic data of the subscribers to the health insurance service.

**Table S3** List of the psychotropic drugs that can be prescribed in Japan and their potencies.

**Table S4** Prescription of concomitant psychotropic drugs.

**Table S5** The proportion of those prescribed three or more psychotropic drugs among subscribers to the health insurance service who were prescribed psychotropic drugs (by 5‐year age group and sex).

**Table S6** Potency of psychotropics.

**Table S7** The proportion of those prescribed high‐potency psychotropics among subscribers to the health insurance service who were prescribed psychotropic drugs (by 5‐year age group and sex).

**Table S8** Monthly prescription rate of psychotropics (by 5‐year age group and sex).
