# Supplementary figures and images for: Changes in psychotropic polypharmacy and high‐potency prescription following policy change: Findings from a large scale Japanese claims database
Source: Psychiatry Clin Neurosci. 2022 Jul 2;76(9):475–7. doi: 10.1111/pcn.13432 (PMC9546399; doi:10.1111/pcn.13432)

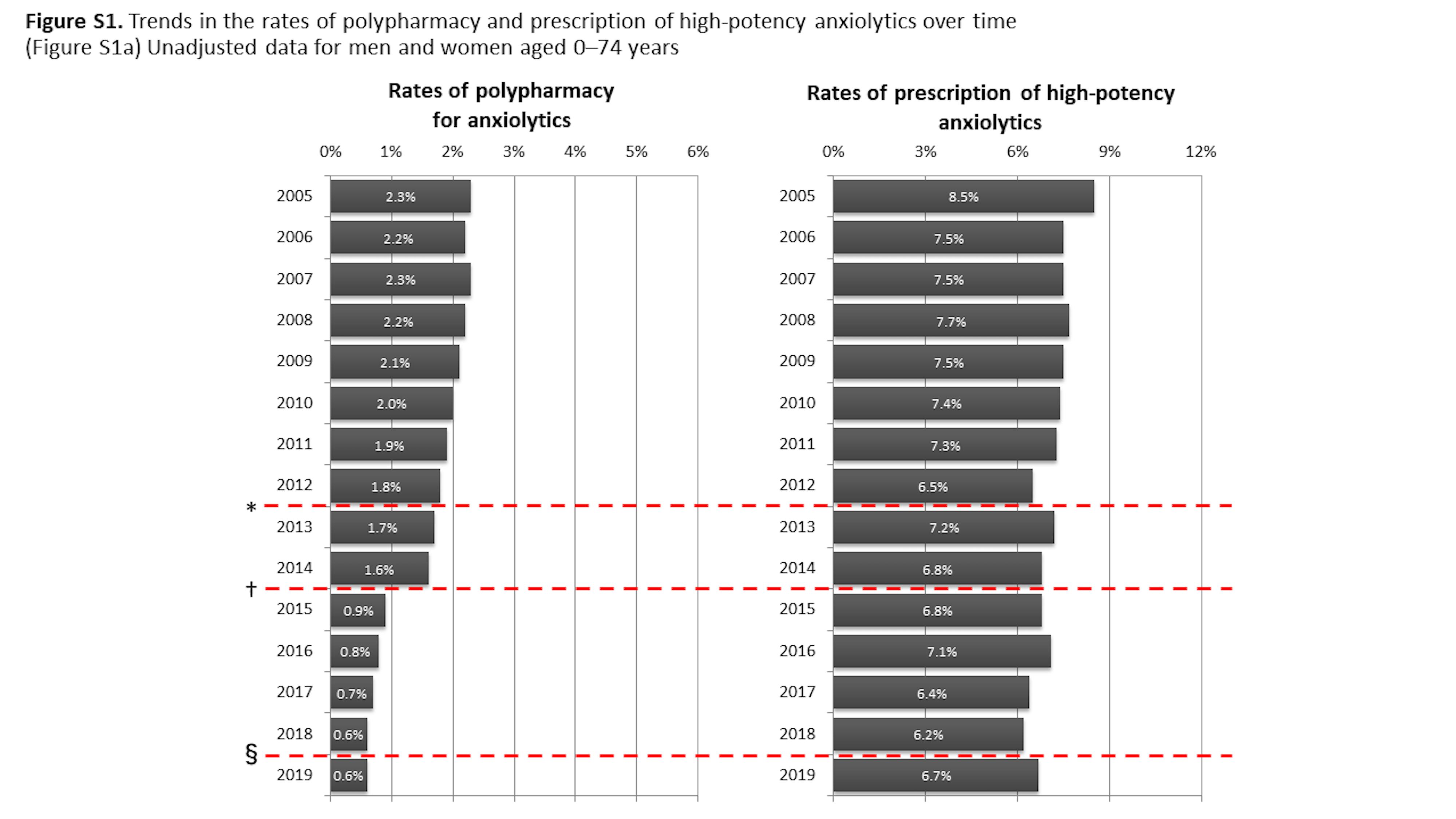

Supplement: Supplementary file 1 — Fig. S1 Trends in the rates of polypharmacy and prescription of high‐potency anxiolytics over time. [file PCN-76-475-s003.zip › FigureS1a.TIF]

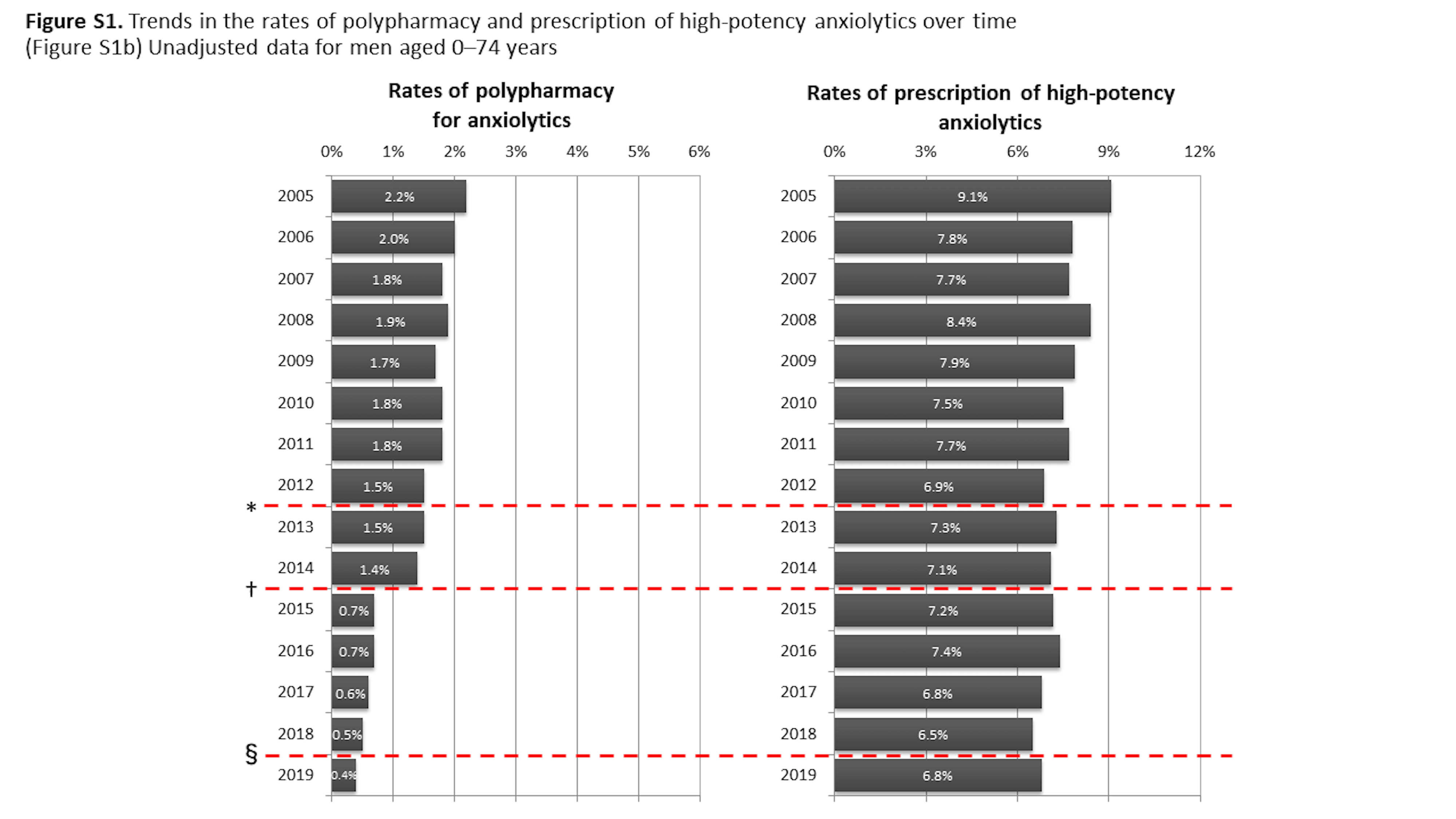

Supplement: Supplementary file 1 — Fig. S1 Trends in the rates of polypharmacy and prescription of high‐potency anxiolytics over time. [file PCN-76-475-s003.zip › FigureS1b.TIF]

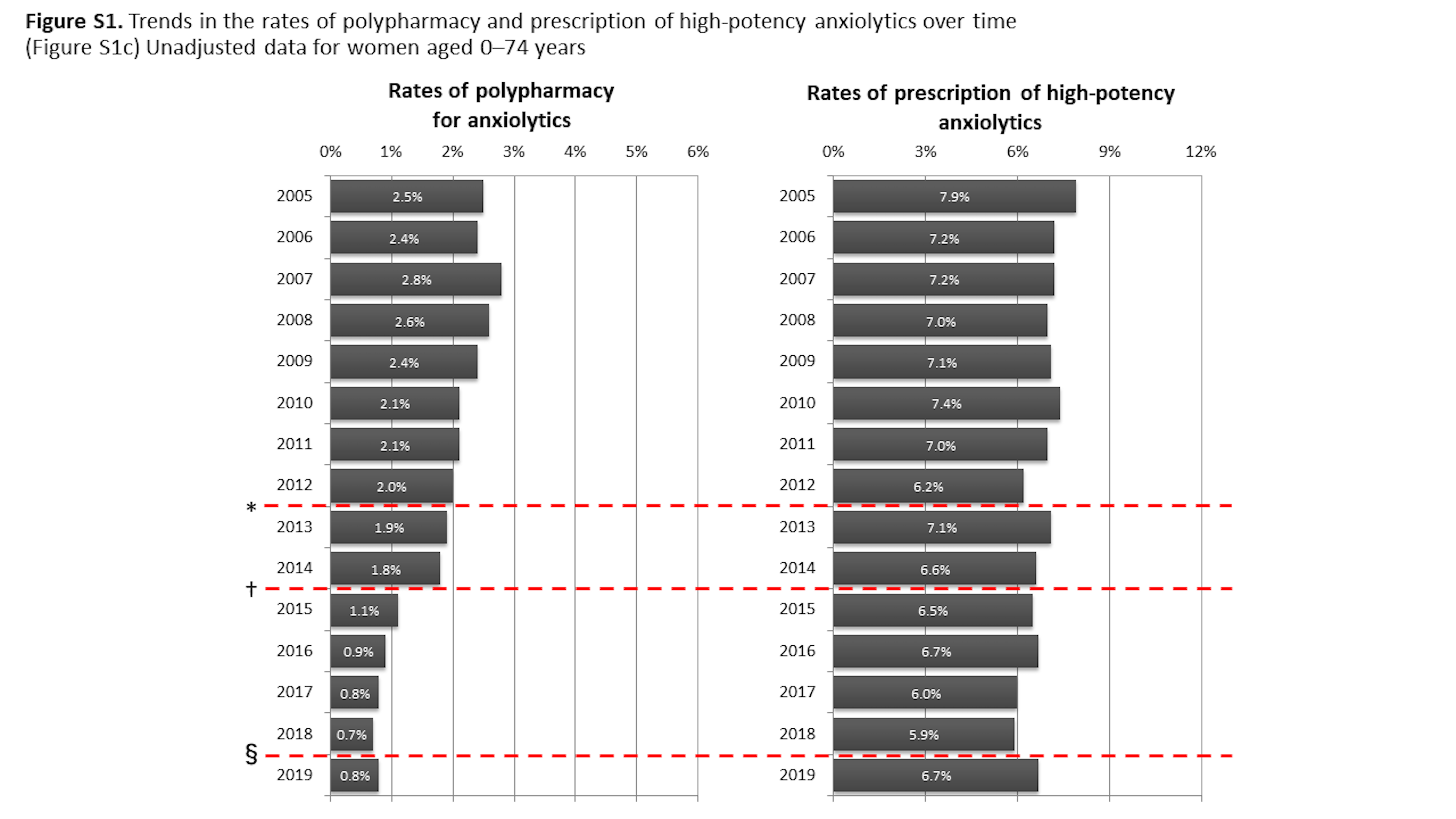

Supplement: Supplementary file 1 — Fig. S1 Trends in the rates of polypharmacy and prescription of high‐potency anxiolytics over time. [file PCN-76-475-s003.zip › FigureS1c.TIF]

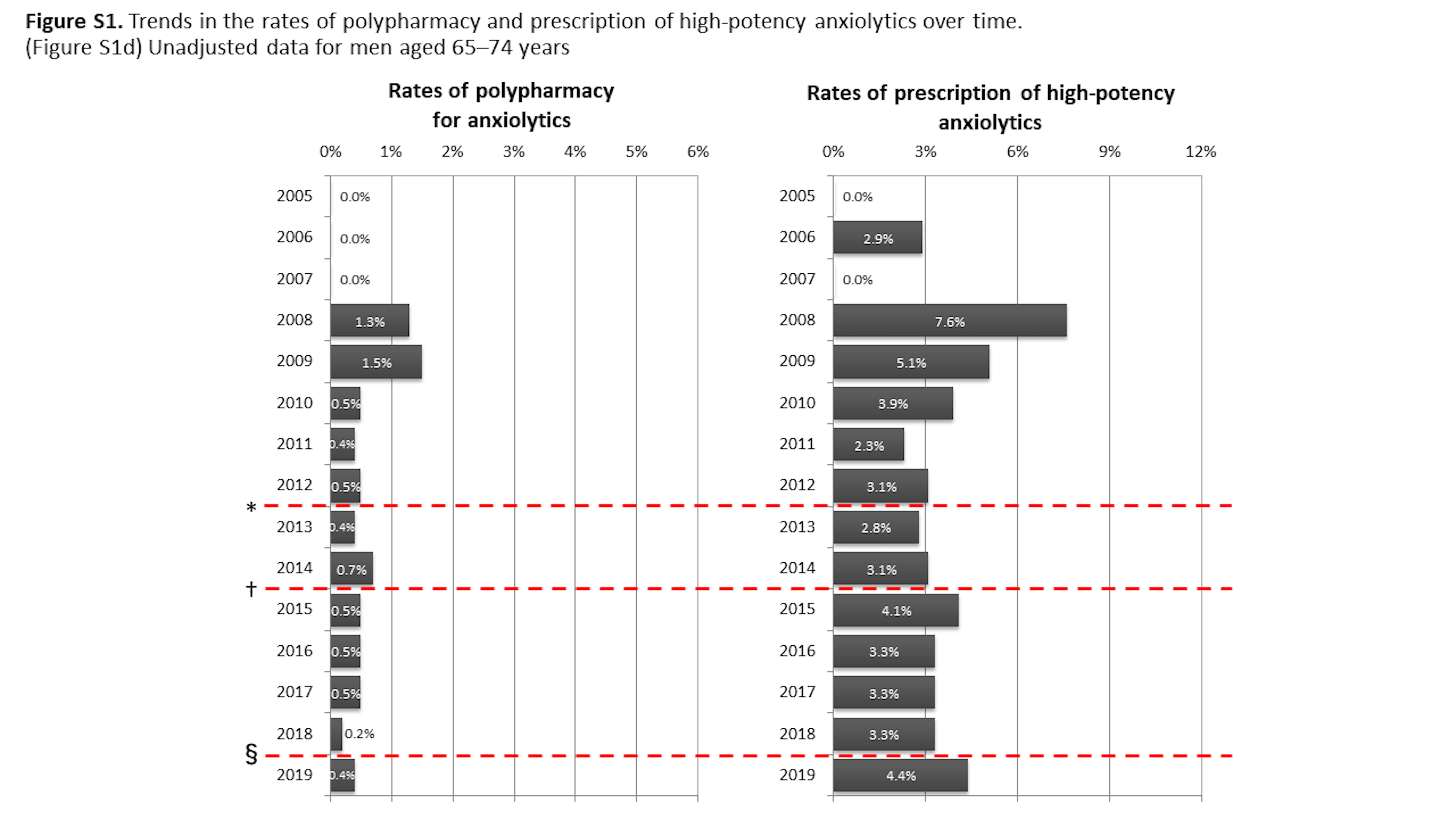

Supplement: Supplementary file 1 — Fig. S1 Trends in the rates of polypharmacy and prescription of high‐potency anxiolytics over time. [file PCN-76-475-s003.zip › FigureS1d.TIF]

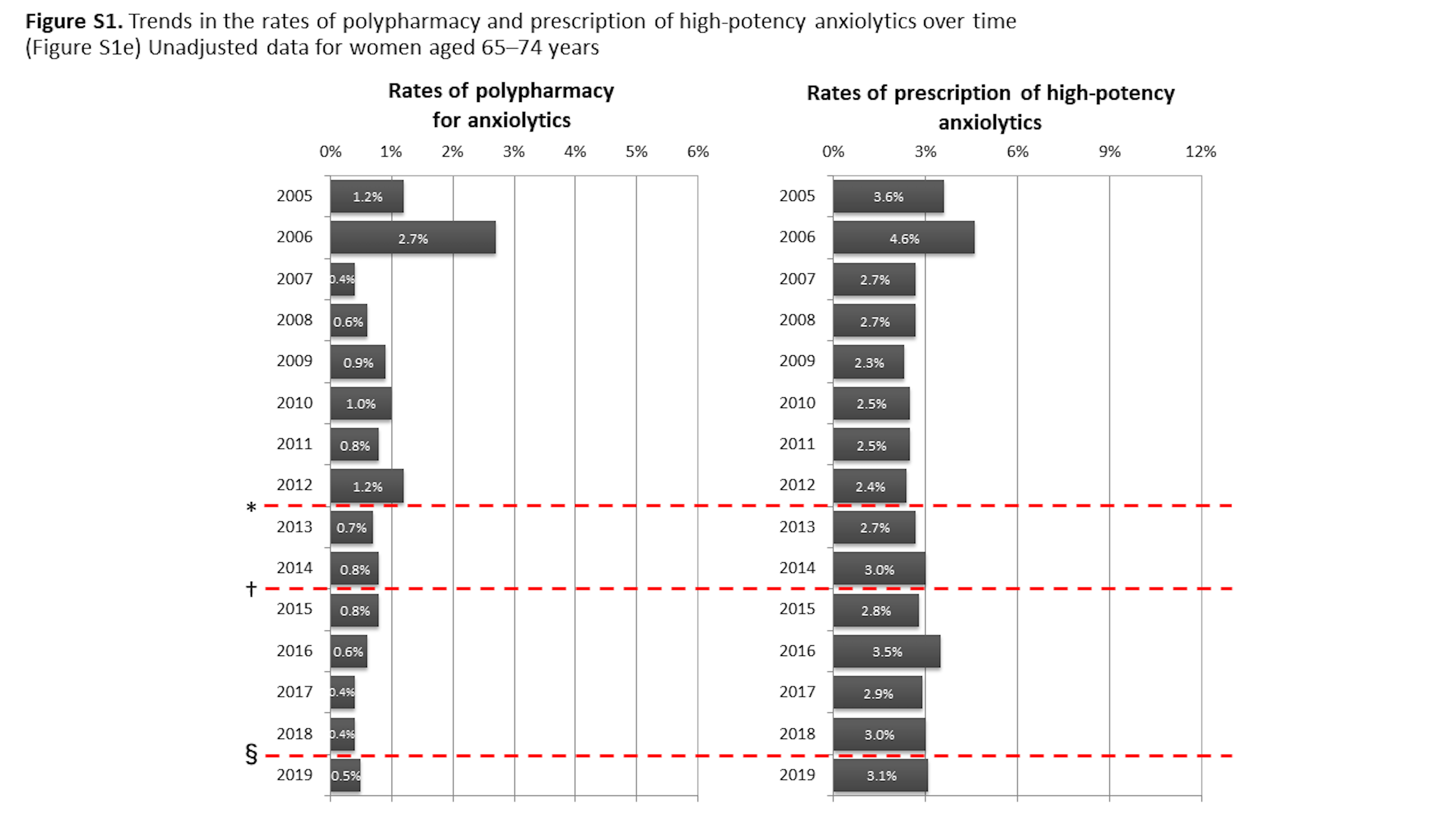

Supplement: Supplementary file 1 — Fig. S1 Trends in the rates of polypharmacy and prescription of high‐potency anxiolytics over time. [file PCN-76-475-s003.zip › FigureS1e.TIF]

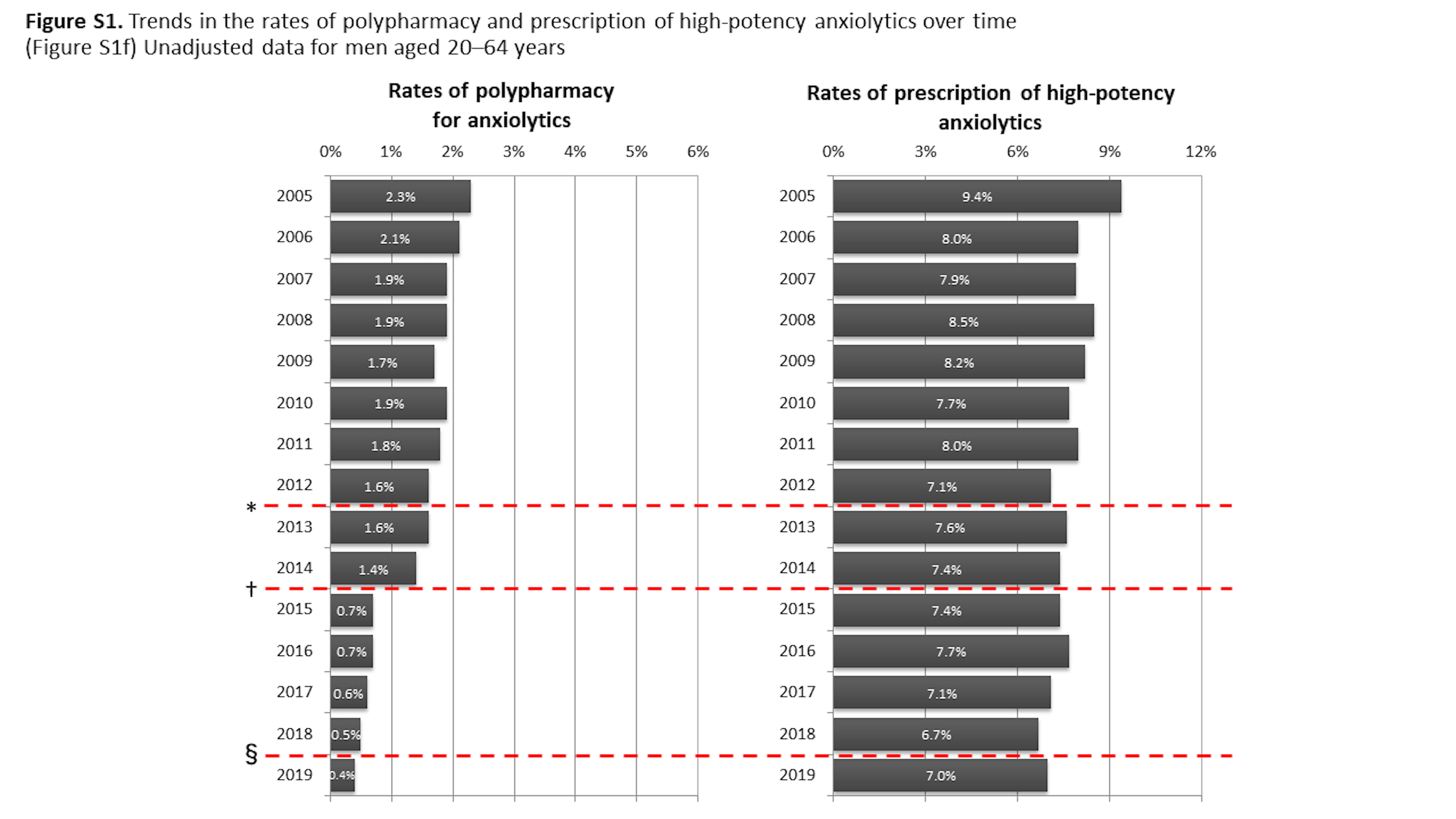

Supplement: Supplementary file 1 — Fig. S1 Trends in the rates of polypharmacy and prescription of high‐potency anxiolytics over time. [file PCN-76-475-s003.zip › FigureS1f.TIF]

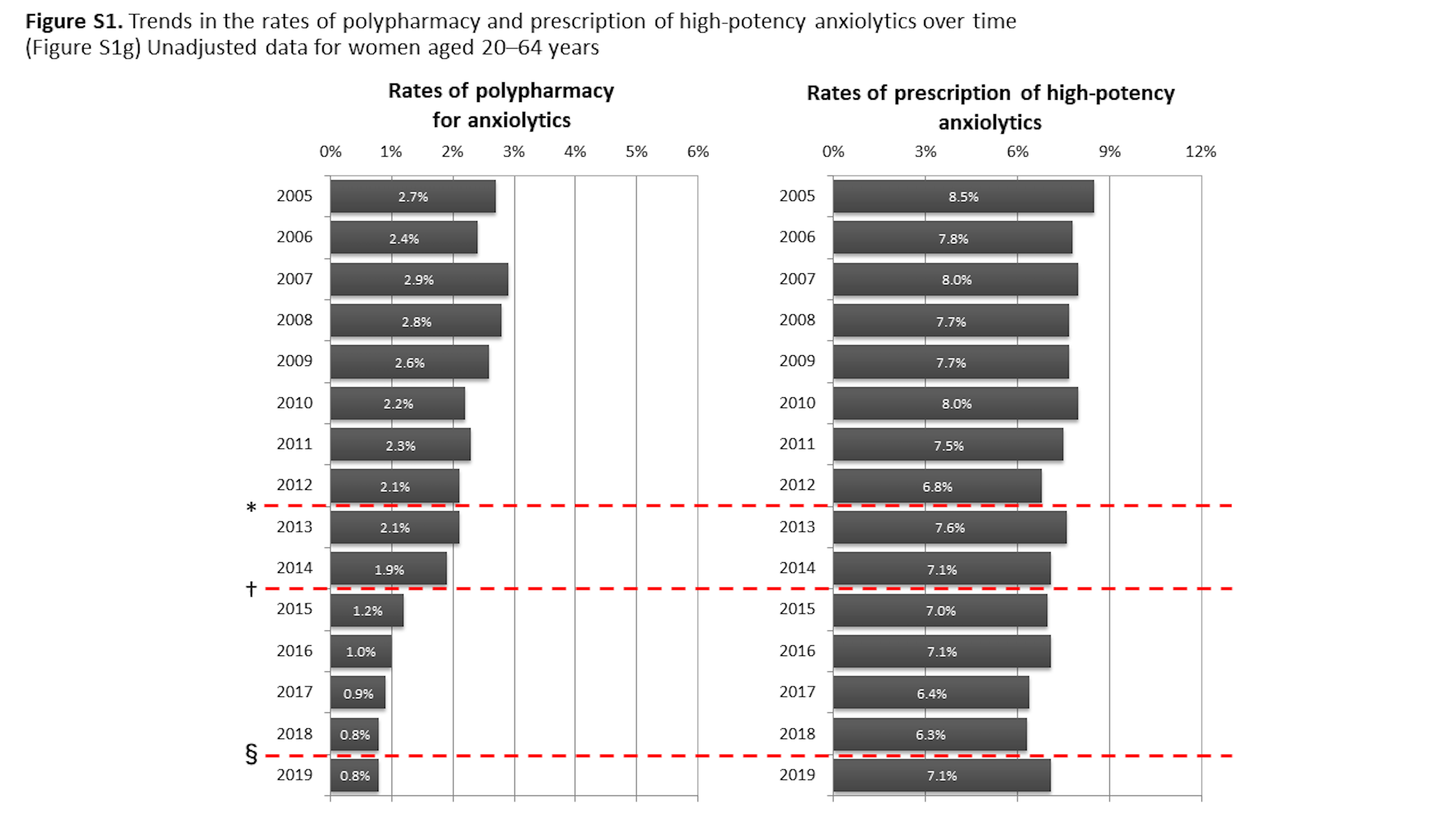

Supplement: Supplementary file 1 — Fig. S1 Trends in the rates of polypharmacy and prescription of high‐potency anxiolytics over time. [file PCN-76-475-s003.zip › FigureS1g.TIF]

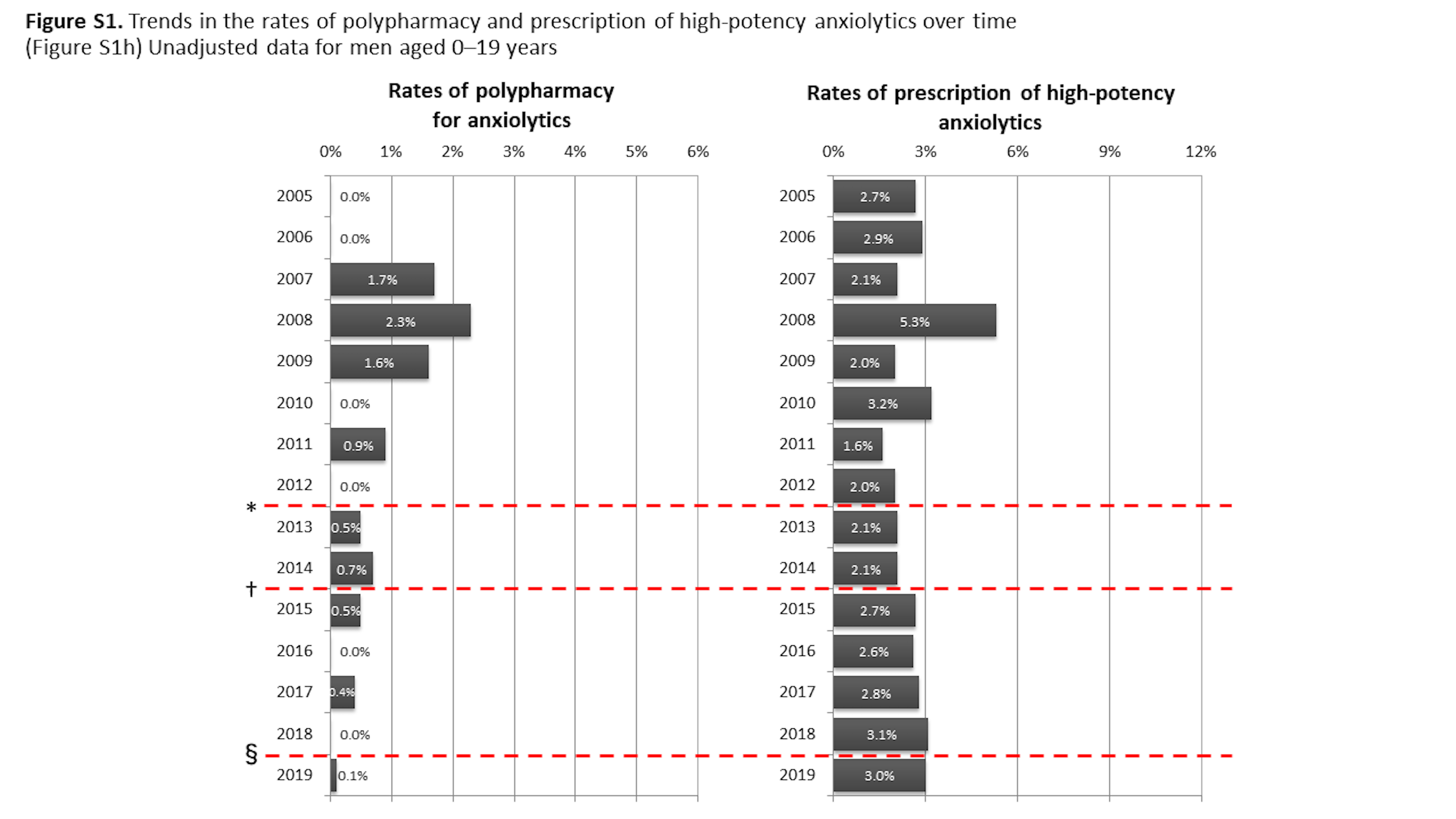

Supplement: Supplementary file 1 — Fig. S1 Trends in the rates of polypharmacy and prescription of high‐potency anxiolytics over time. [file PCN-76-475-s003.zip › FigureS1h.TIF]

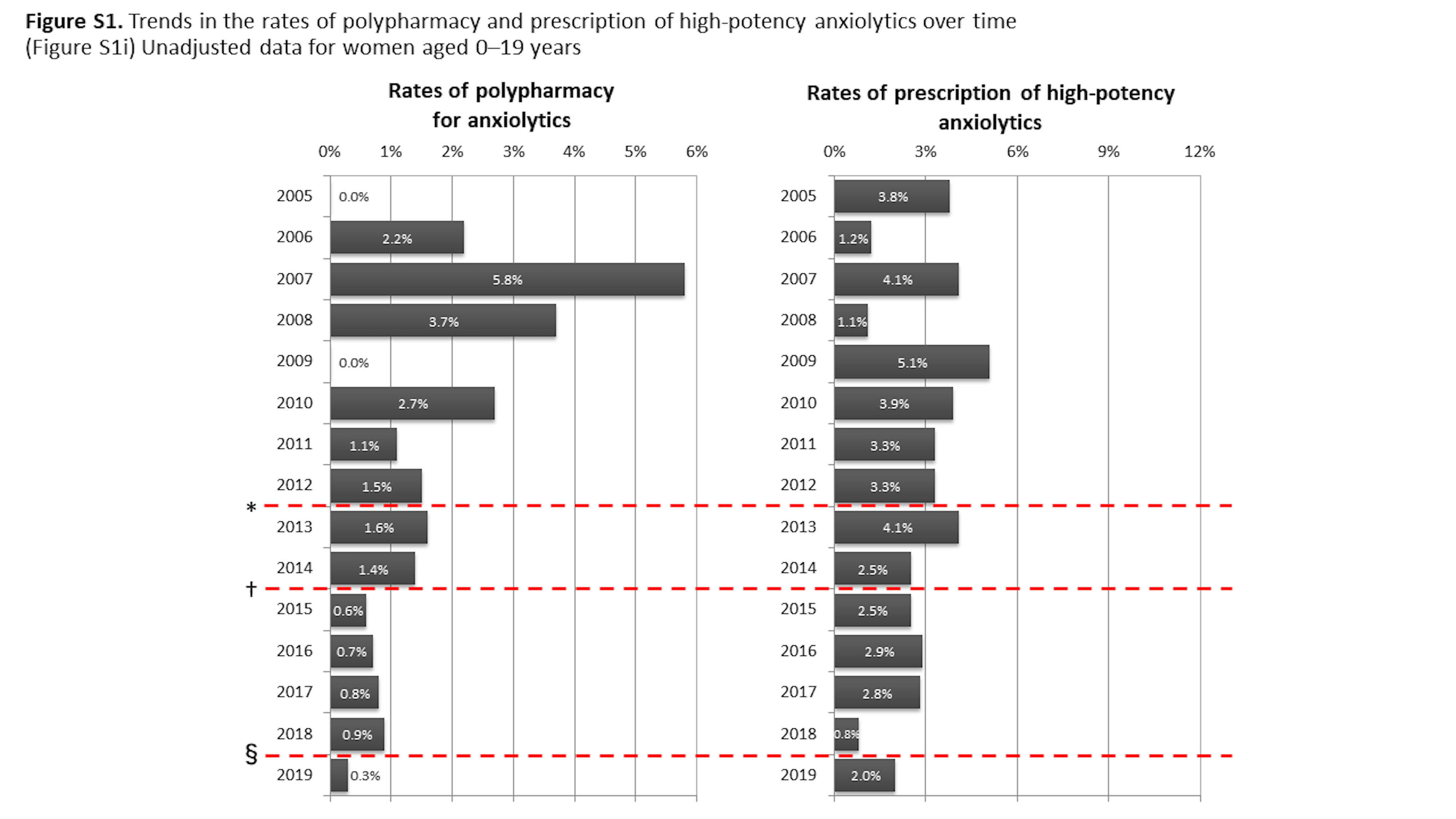

Supplement: Supplementary file 1 — Fig. S1 Trends in the rates of polypharmacy and prescription of high‐potency anxiolytics over time. [file PCN-76-475-s003.zip › FigureS1i.TIF]

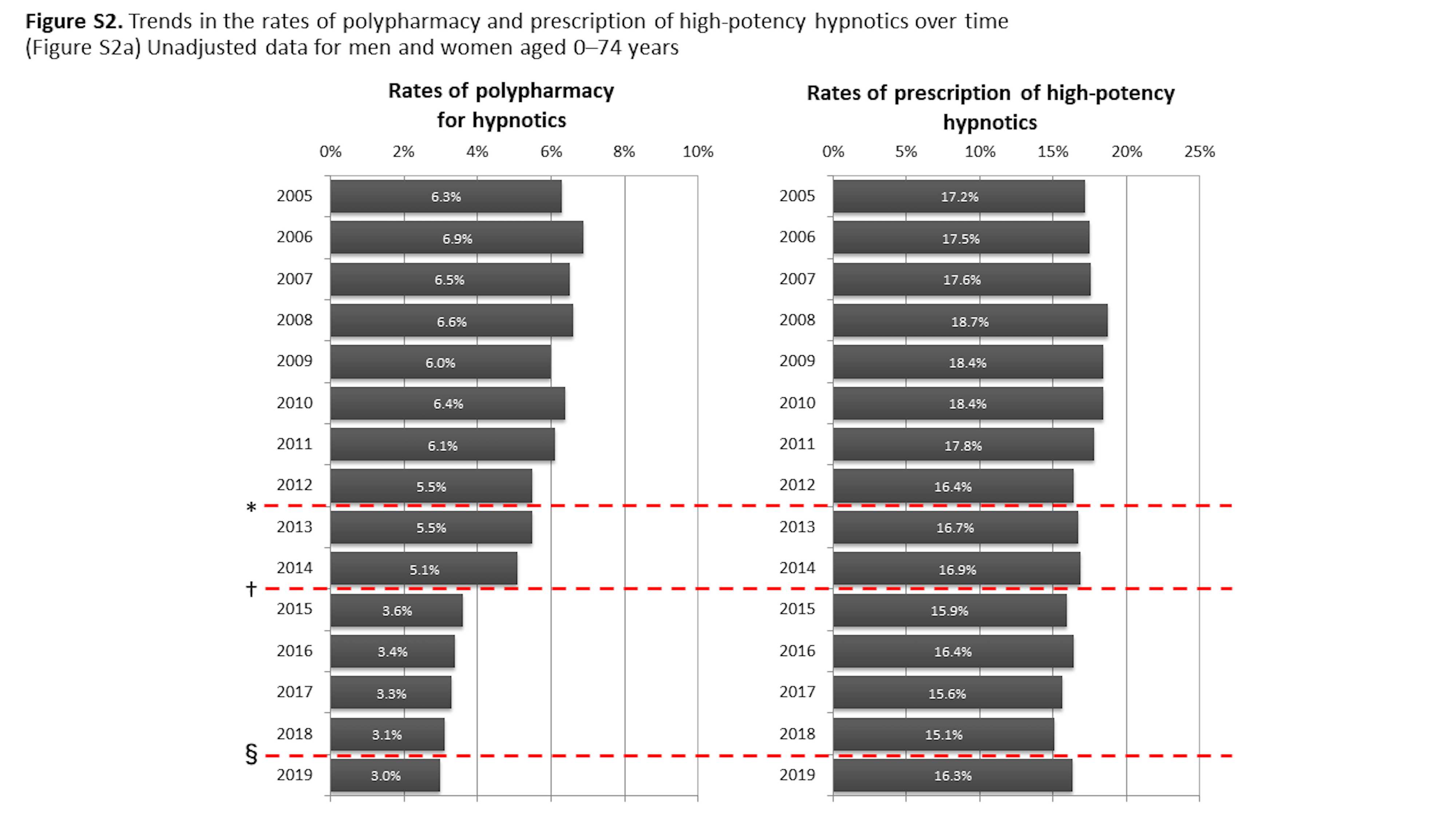

Supplement: Supplementary file 2 — Fig. S2 Trends in the rates of polypharmacy and prescription of high‐potency hypnotics over time. [file PCN-76-475-s002.zip › FigureS2a.TIF]

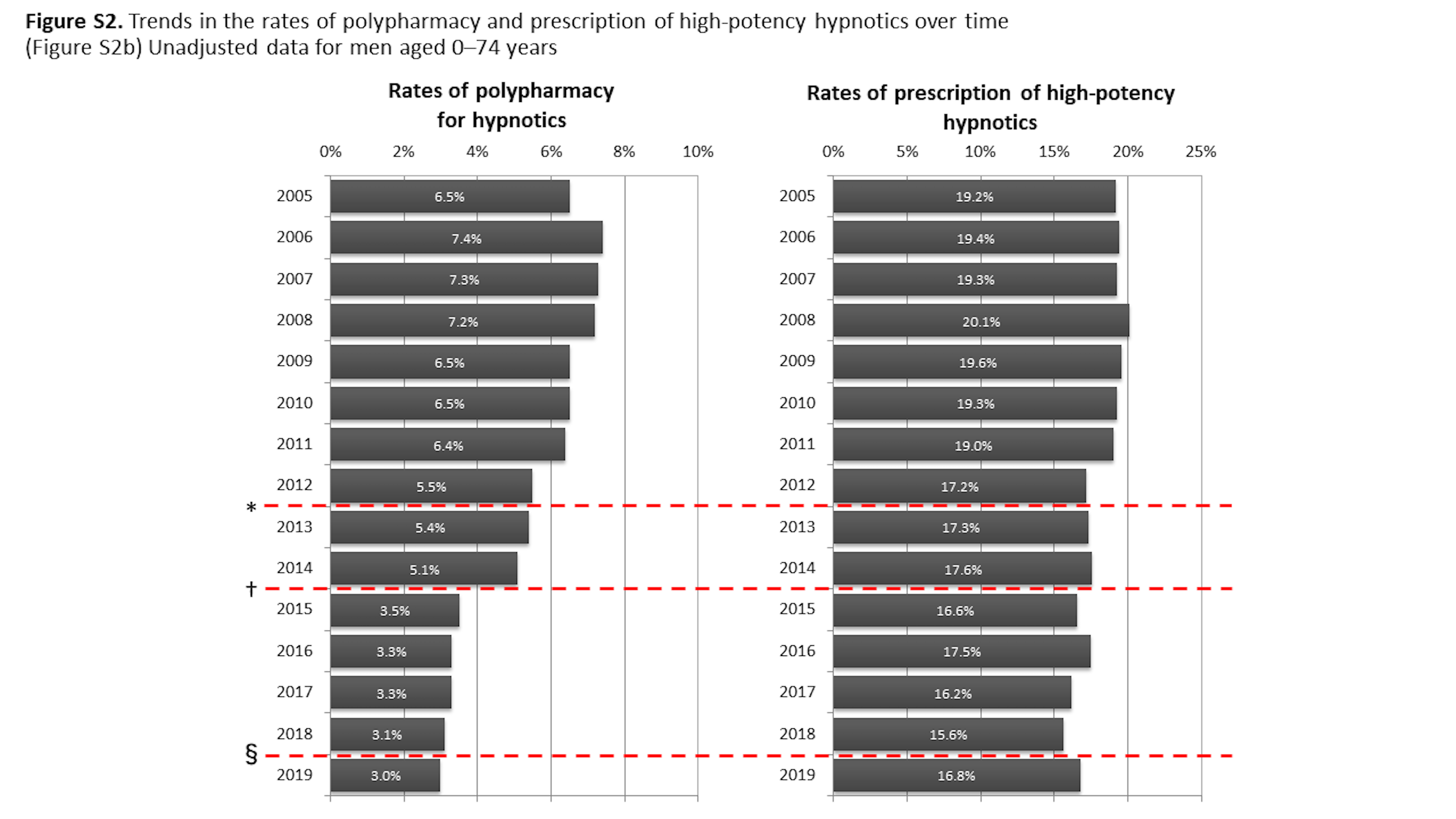

Supplement: Supplementary file 2 — Fig. S2 Trends in the rates of polypharmacy and prescription of high‐potency hypnotics over time. [file PCN-76-475-s002.zip › FigureS2b.TIF]

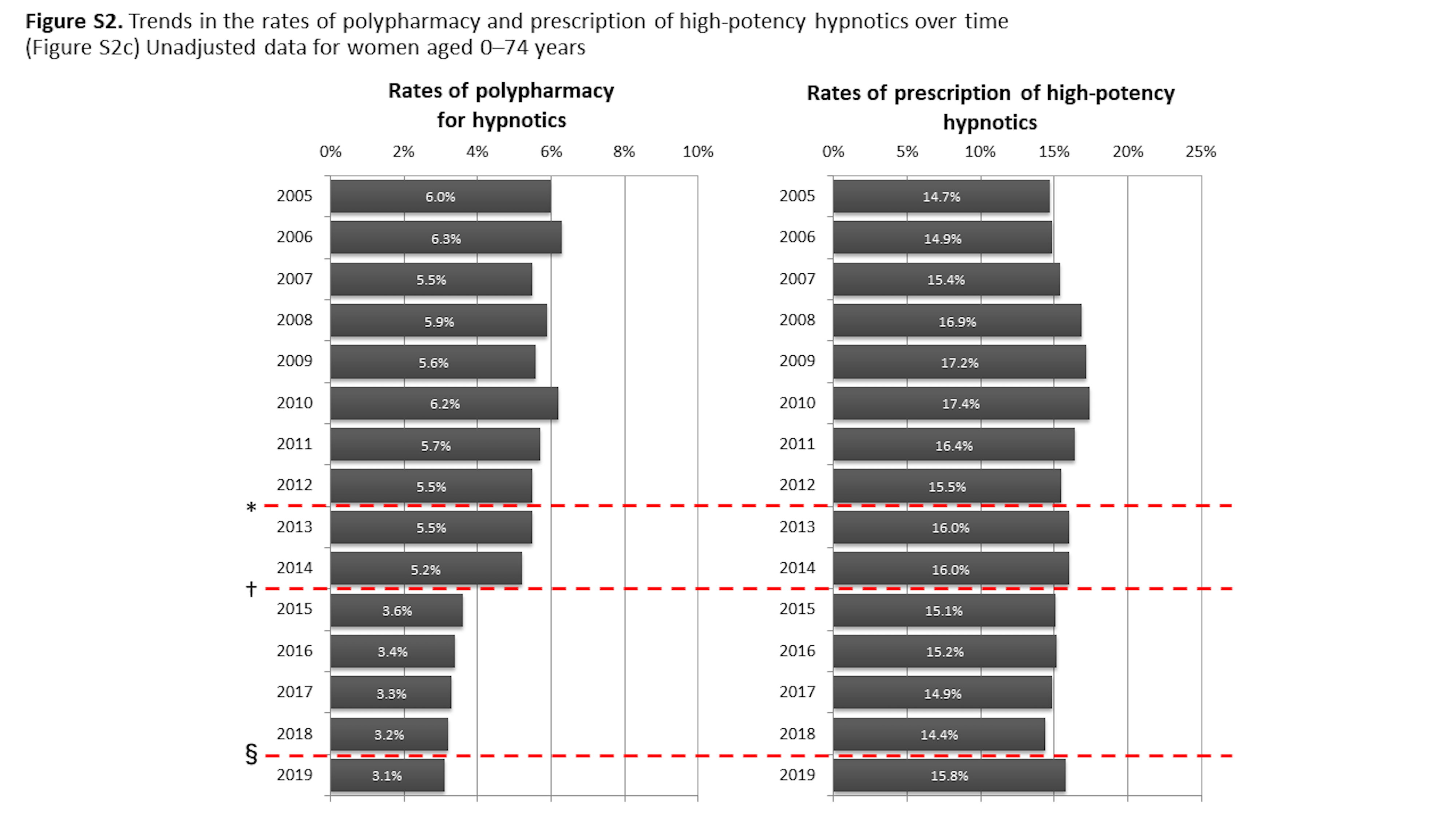

Supplement: Supplementary file 2 — Fig. S2 Trends in the rates of polypharmacy and prescription of high‐potency hypnotics over time. [file PCN-76-475-s002.zip › FigureS2c.TIF]

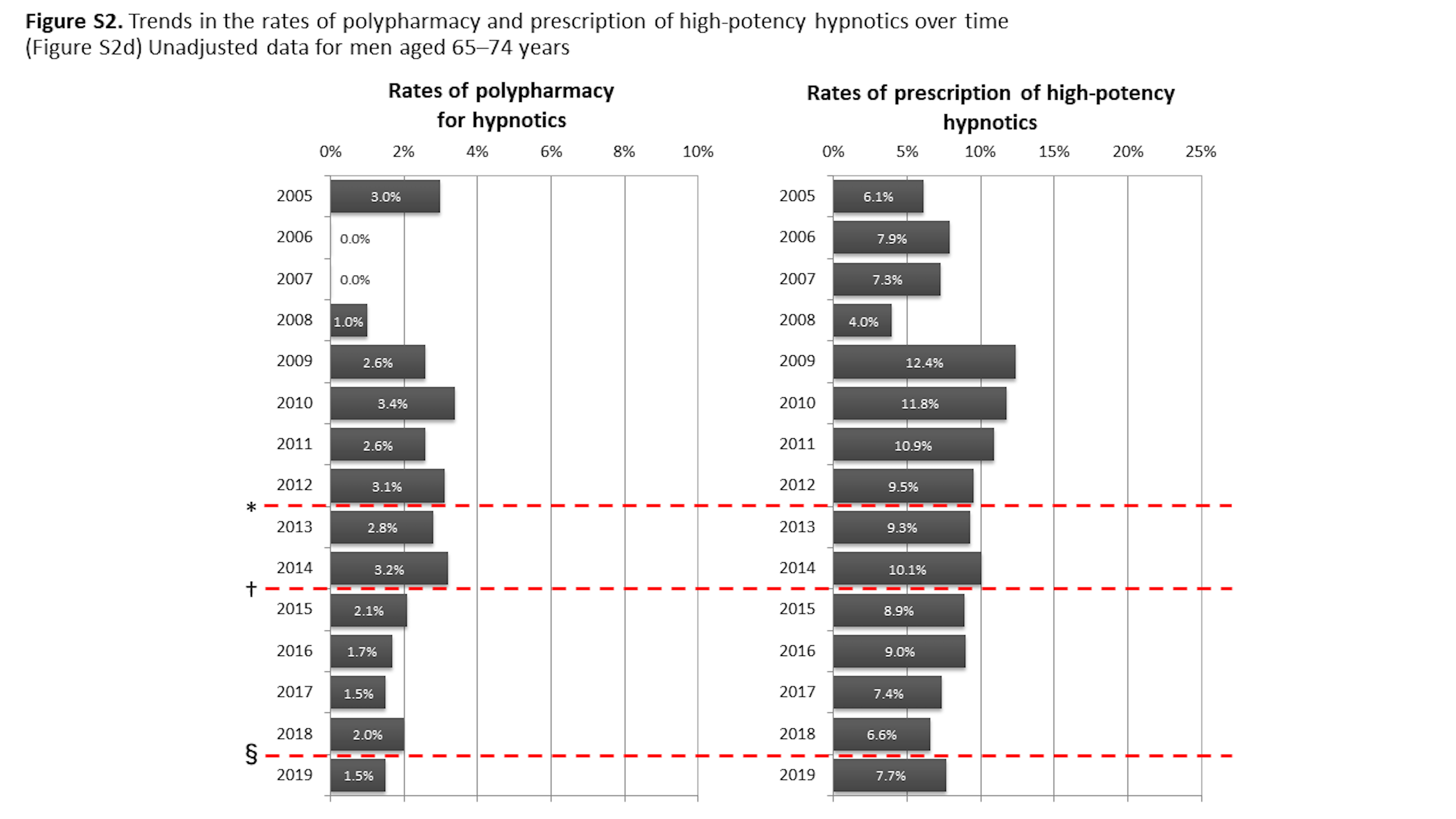

Supplement: Supplementary file 2 — Fig. S2 Trends in the rates of polypharmacy and prescription of high‐potency hypnotics over time. [file PCN-76-475-s002.zip › FigureS2d.TIF]

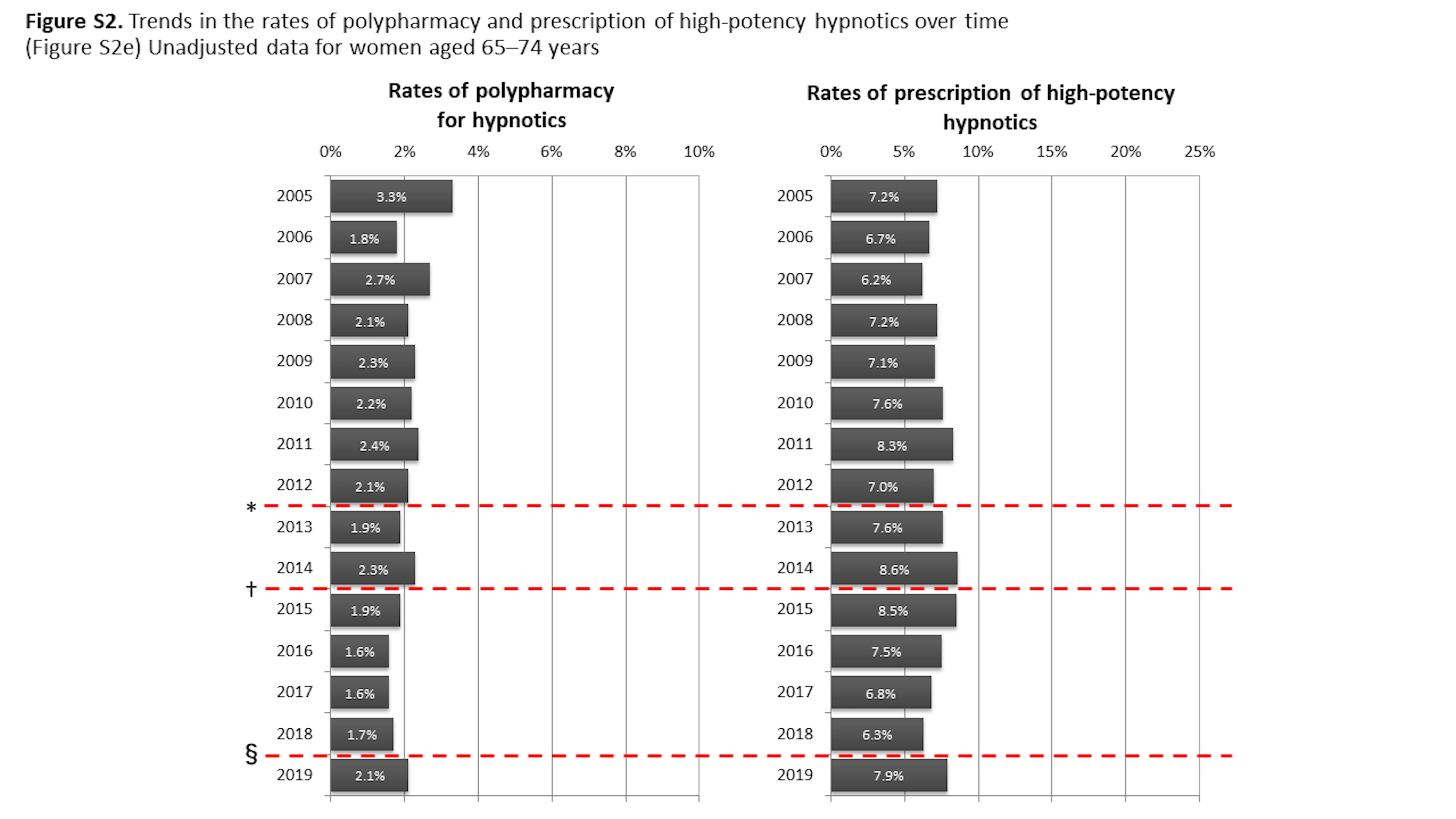

Supplement: Supplementary file 2 — Fig. S2 Trends in the rates of polypharmacy and prescription of high‐potency hypnotics over time. [file PCN-76-475-s002.zip › FigureS2e.TIF]

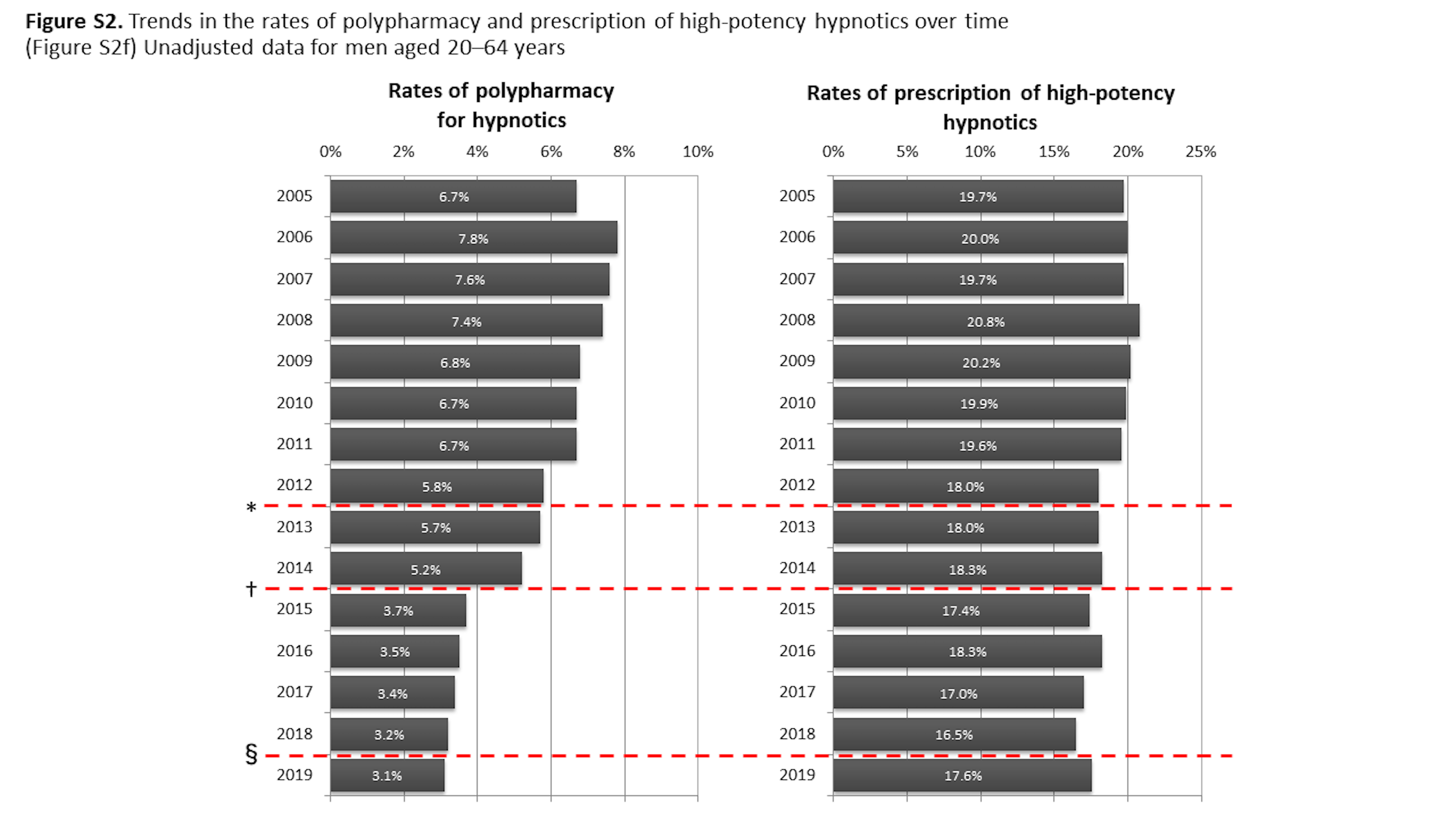

Supplement: Supplementary file 2 — Fig. S2 Trends in the rates of polypharmacy and prescription of high‐potency hypnotics over time. [file PCN-76-475-s002.zip › FigureS2f.TIF]

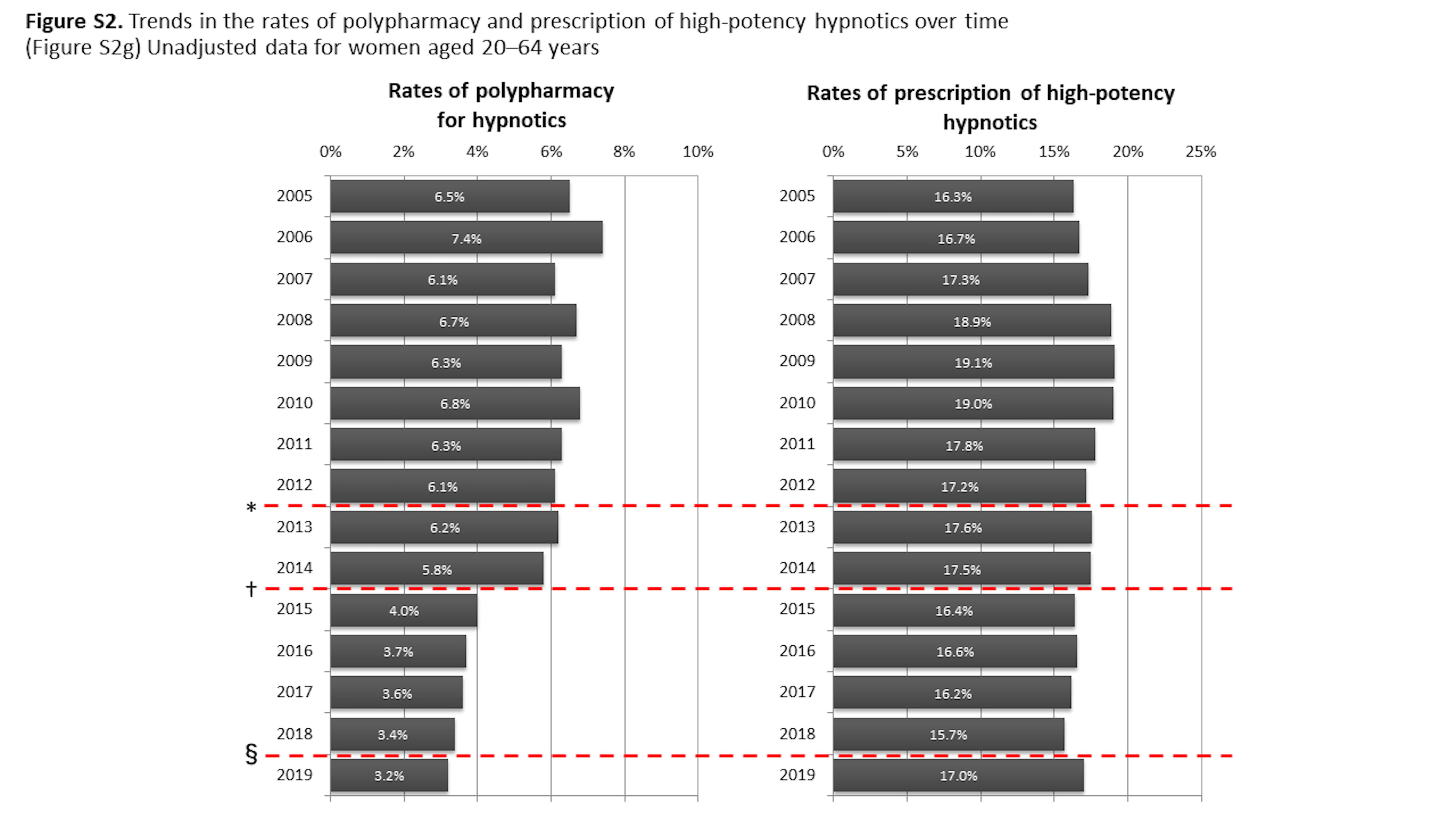

Supplement: Supplementary file 2 — Fig. S2 Trends in the rates of polypharmacy and prescription of high‐potency hypnotics over time. [file PCN-76-475-s002.zip › FigureS2g.TIF]

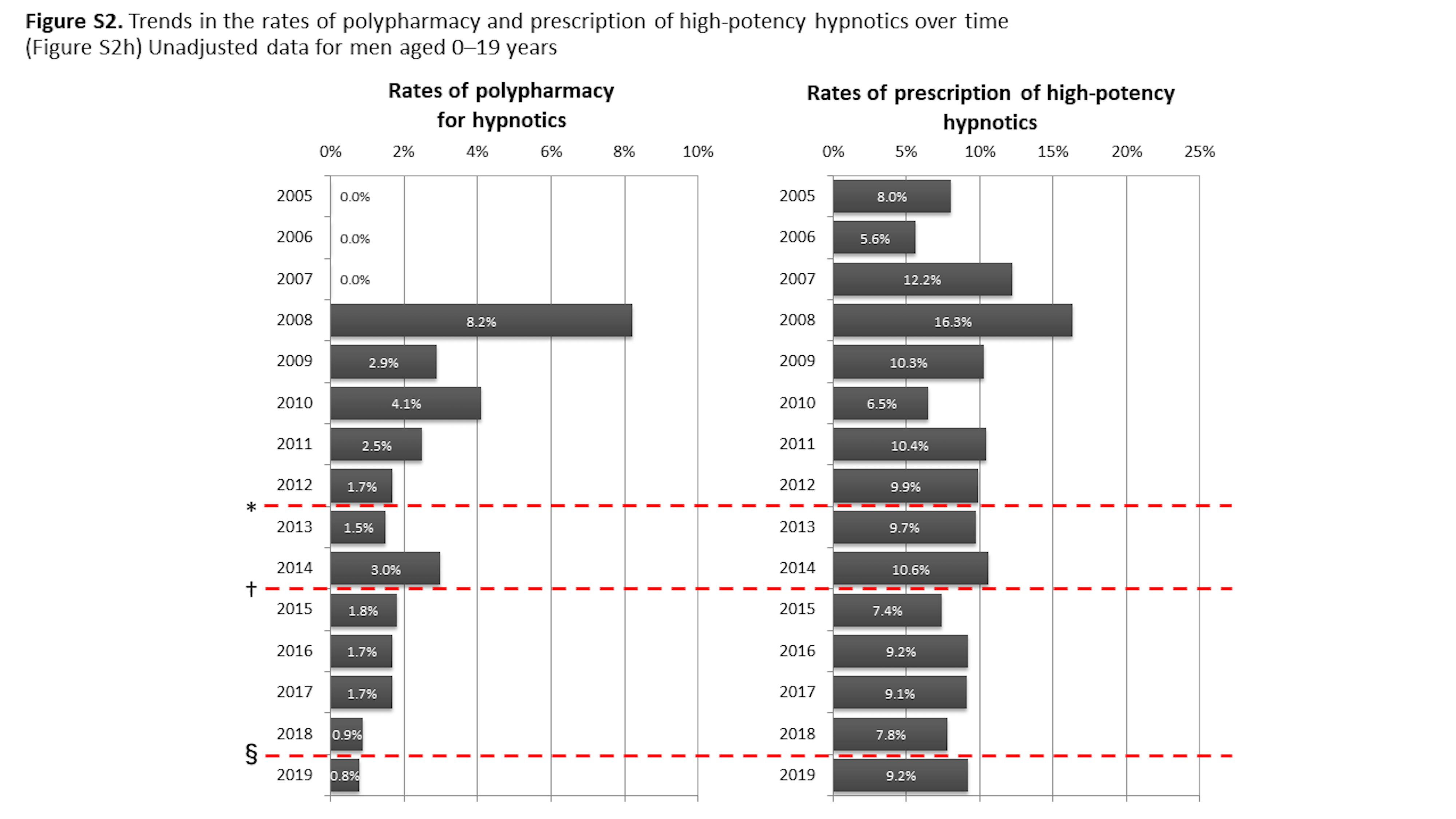

Supplement: Supplementary file 2 — Fig. S2 Trends in the rates of polypharmacy and prescription of high‐potency hypnotics over time. [file PCN-76-475-s002.zip › FigureS2h.TIF]

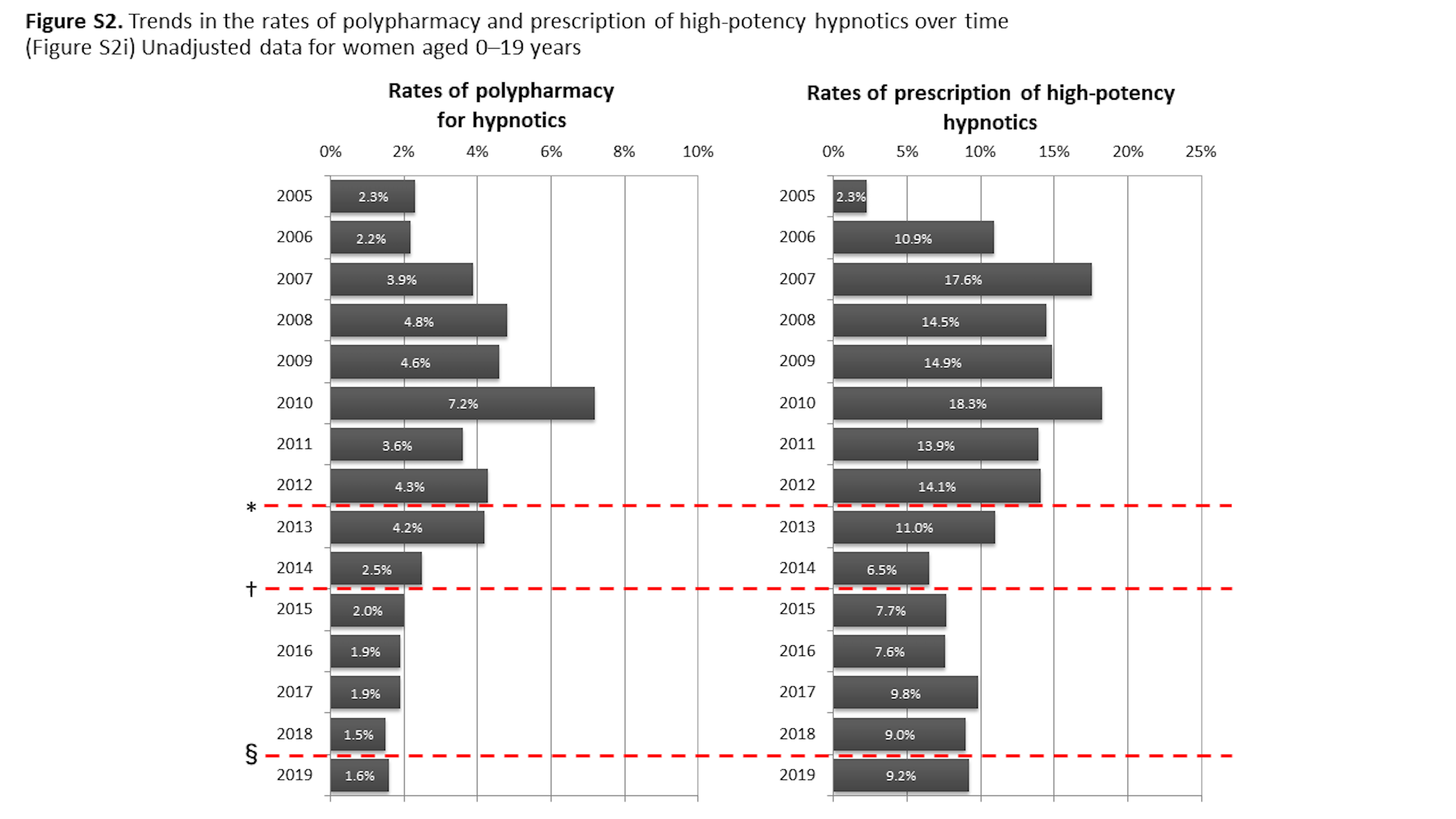

Supplement: Supplementary file 2 — Fig. S2 Trends in the rates of polypharmacy and prescription of high‐potency hypnotics over time. [file PCN-76-475-s002.zip › FigureS2i.TIF]

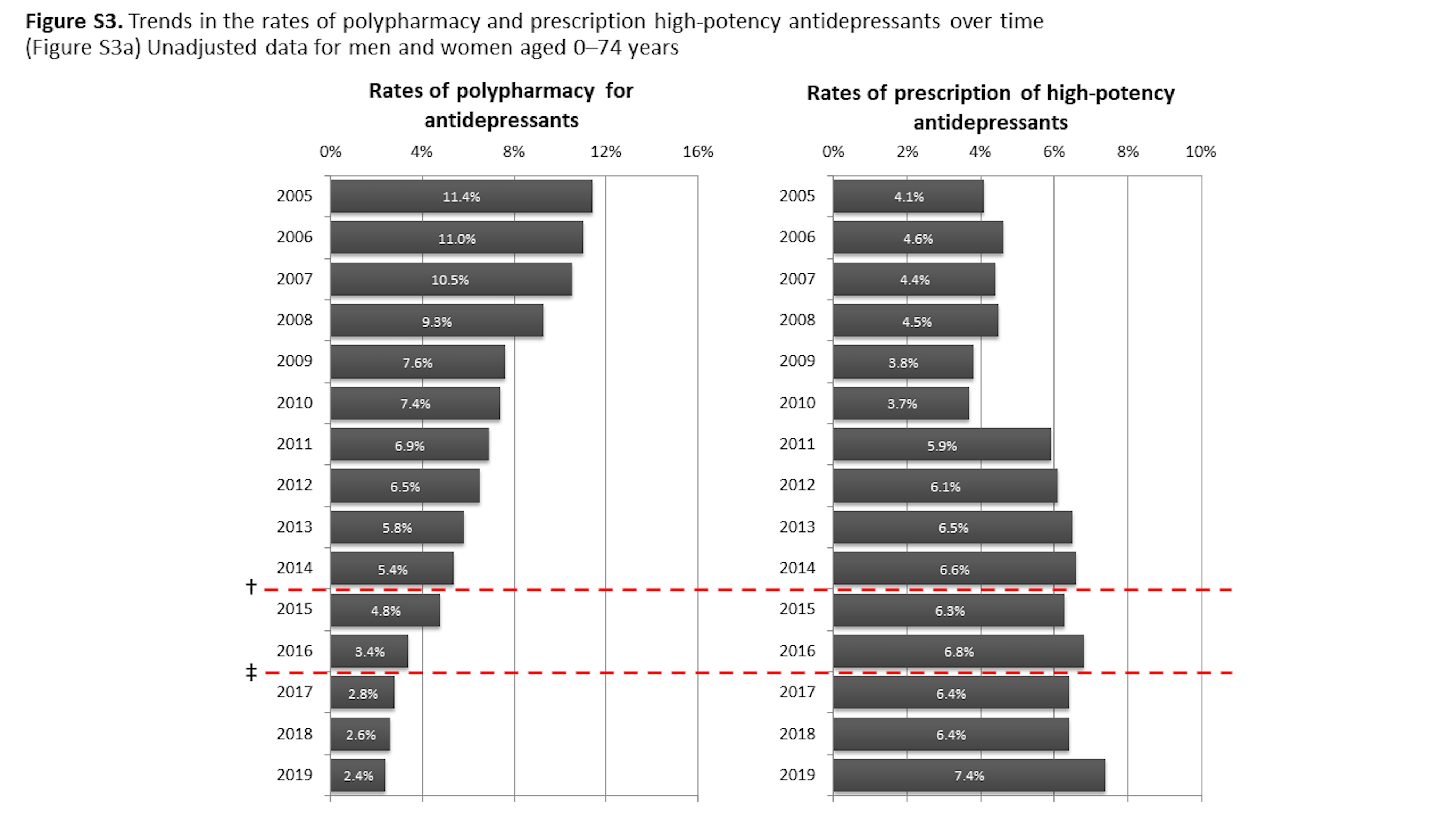

Supplement: Supplementary file 3 — Fig. S3 Trends in the rates of polypharmacy and prescription of high‐potency antidepressants over time. [file PCN-76-475-s008.zip › FigureS3a.TIF]

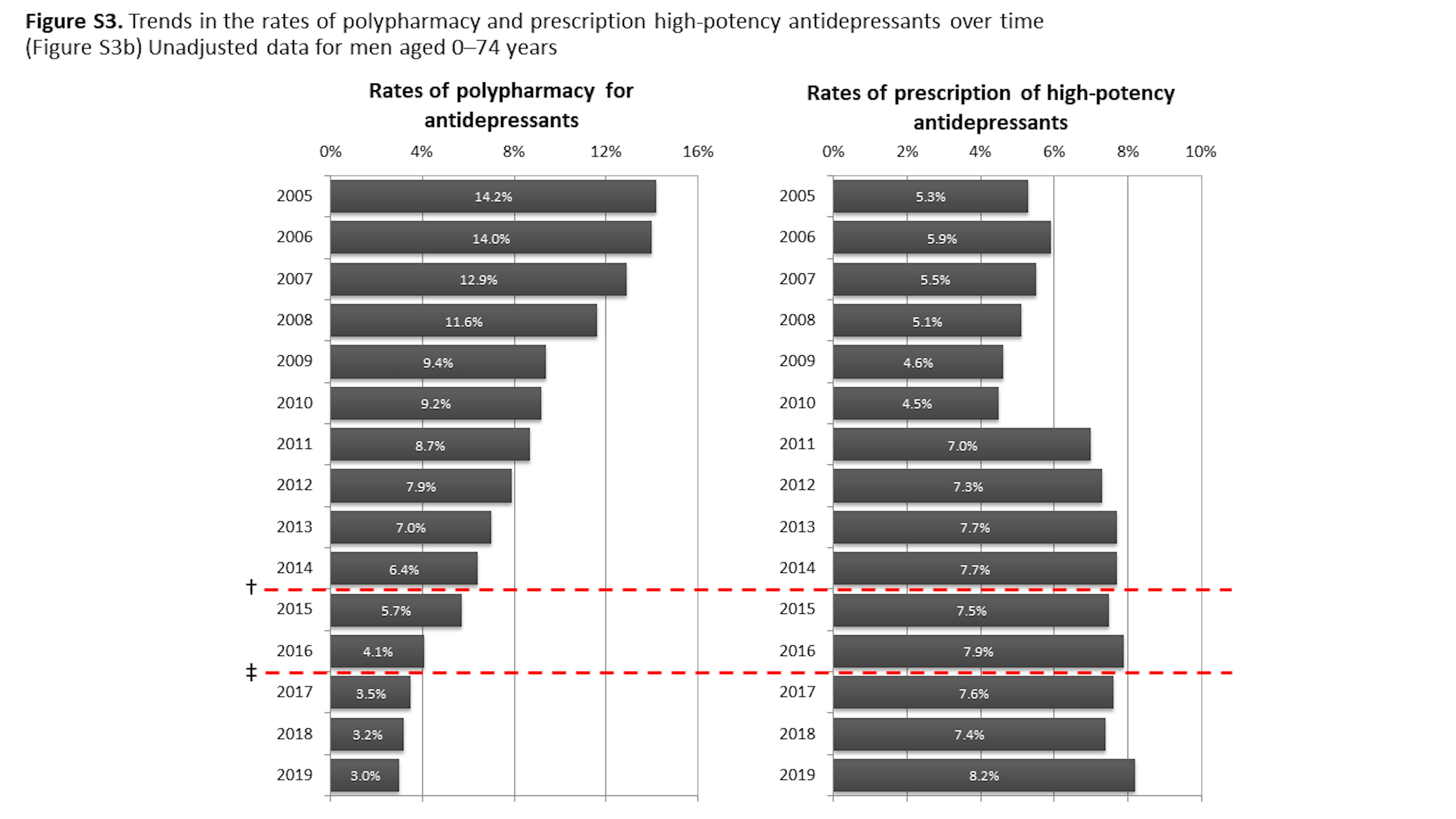

Supplement: Supplementary file 3 — Fig. S3 Trends in the rates of polypharmacy and prescription of high‐potency antidepressants over time. [file PCN-76-475-s008.zip › FigureS3b.TIF]

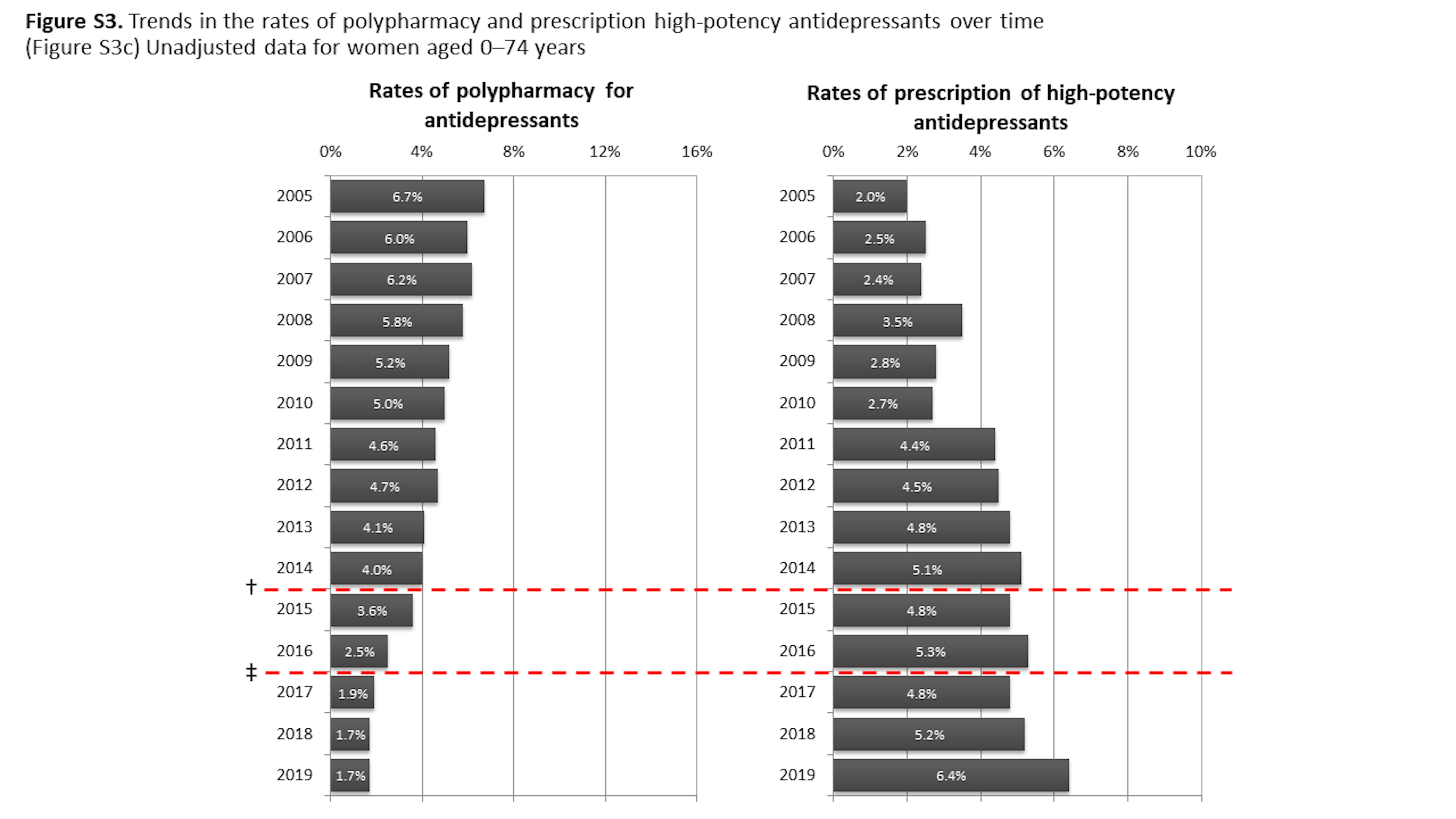

Supplement: Supplementary file 3 — Fig. S3 Trends in the rates of polypharmacy and prescription of high‐potency antidepressants over time. [file PCN-76-475-s008.zip › FigureS3c.TIF]

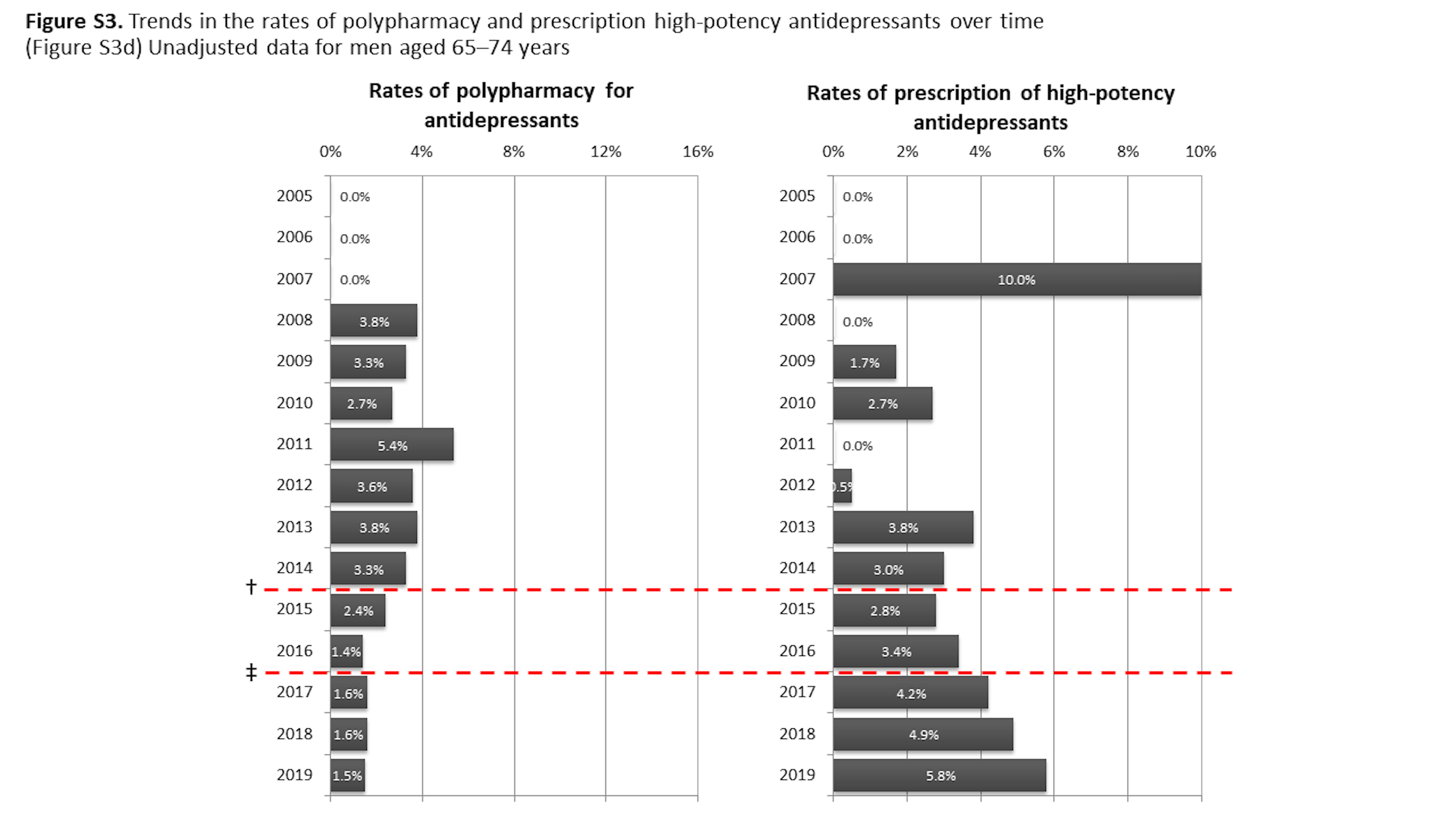

Supplement: Supplementary file 3 — Fig. S3 Trends in the rates of polypharmacy and prescription of high‐potency antidepressants over time. [file PCN-76-475-s008.zip › FigureS3d.TIF]

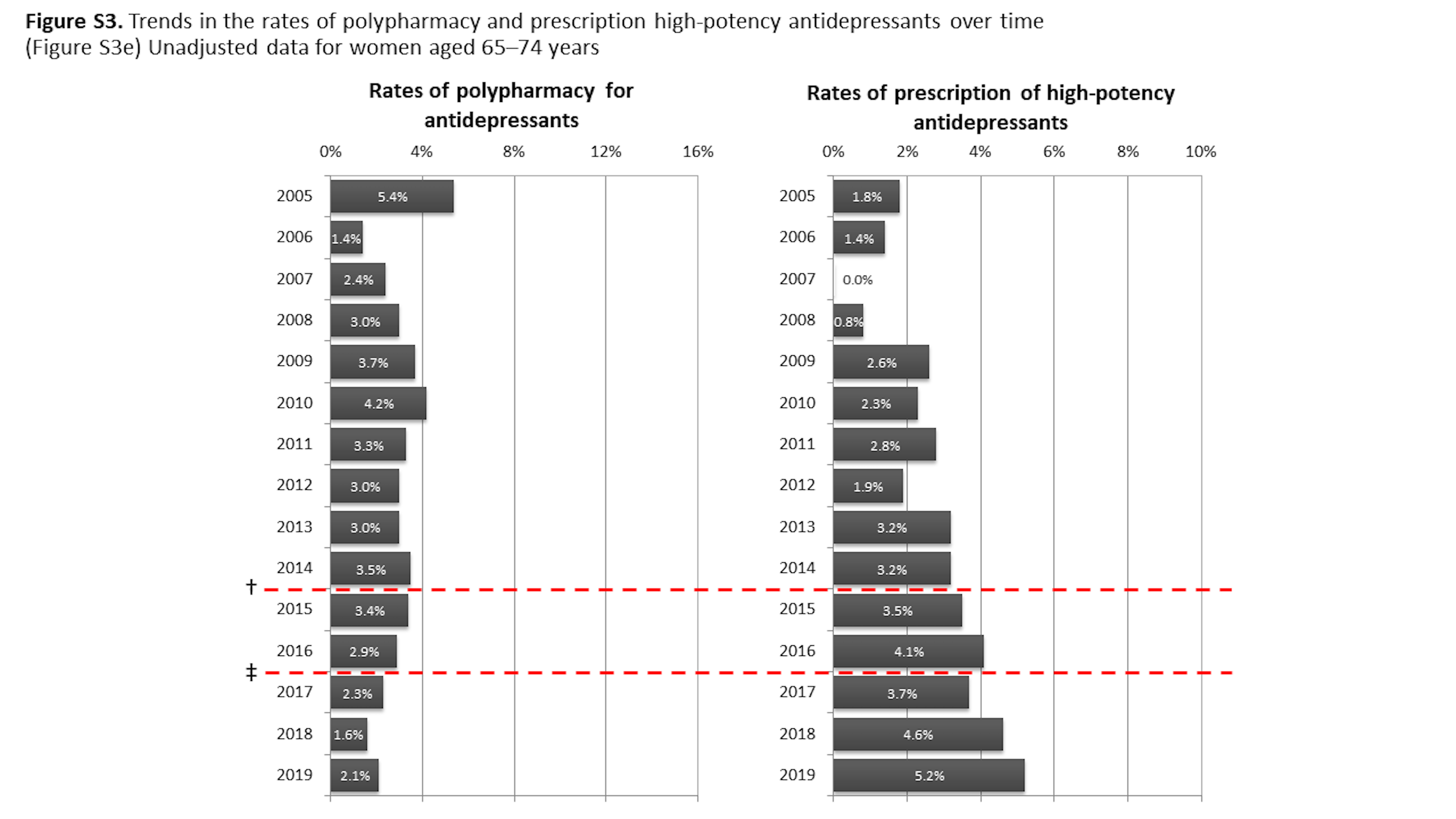

Supplement: Supplementary file 3 — Fig. S3 Trends in the rates of polypharmacy and prescription of high‐potency antidepressants over time. [file PCN-76-475-s008.zip › FigureS3e.TIF]

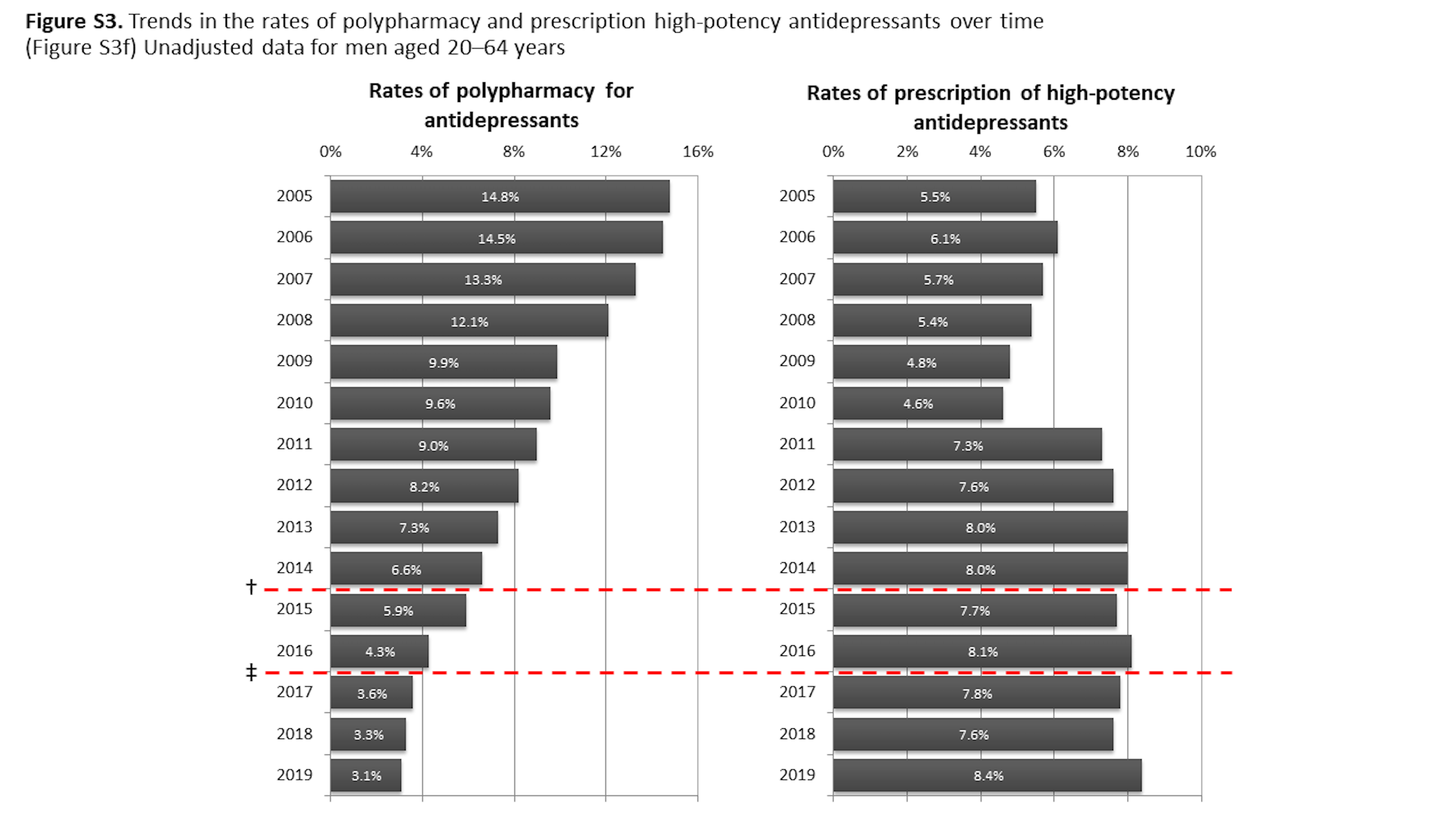

Supplement: Supplementary file 3 — Fig. S3 Trends in the rates of polypharmacy and prescription of high‐potency antidepressants over time. [file PCN-76-475-s008.zip › FigureS3f.TIF]

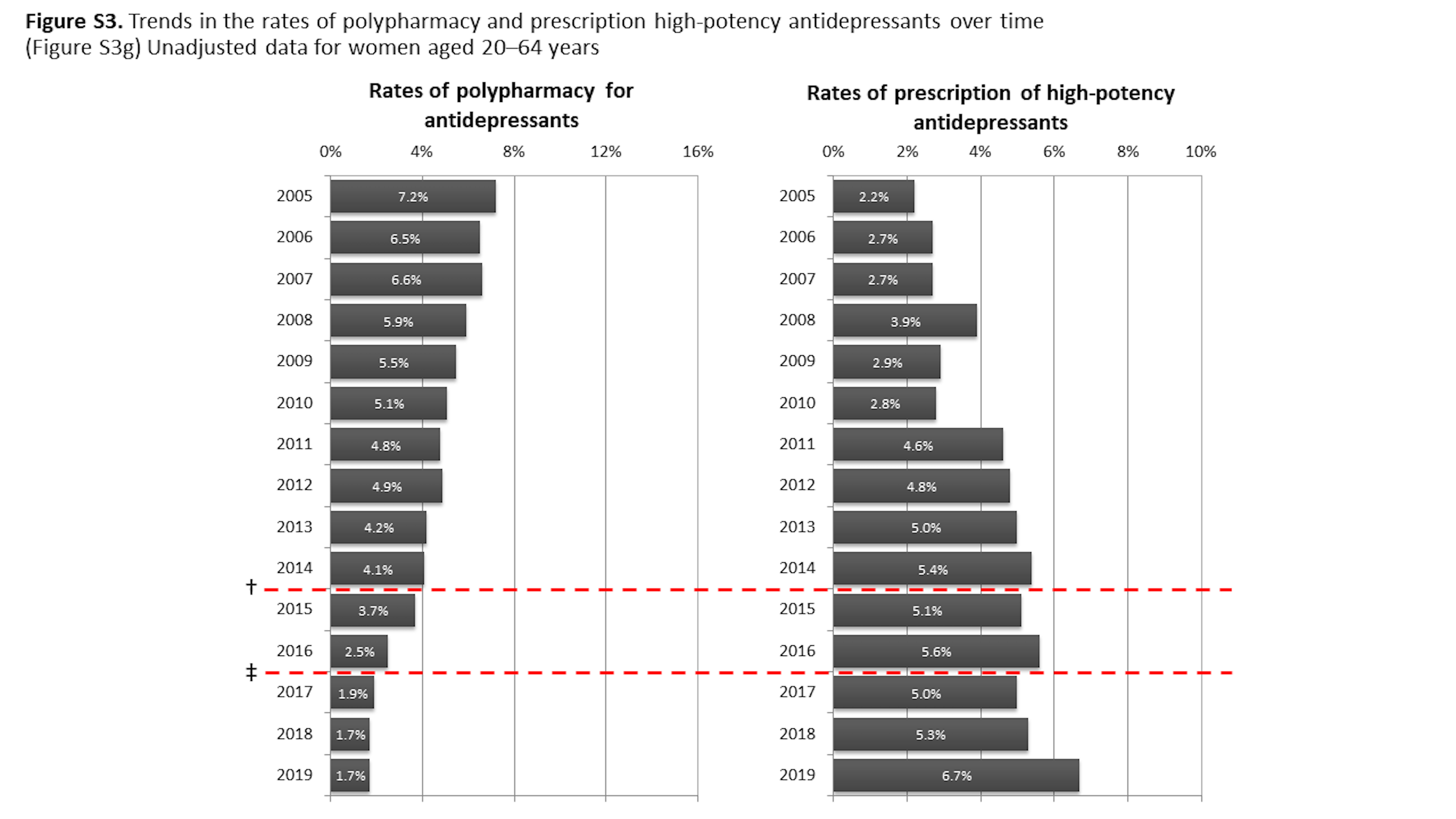

Supplement: Supplementary file 3 — Fig. S3 Trends in the rates of polypharmacy and prescription of high‐potency antidepressants over time. [file PCN-76-475-s008.zip › FigureS3g.TIF]

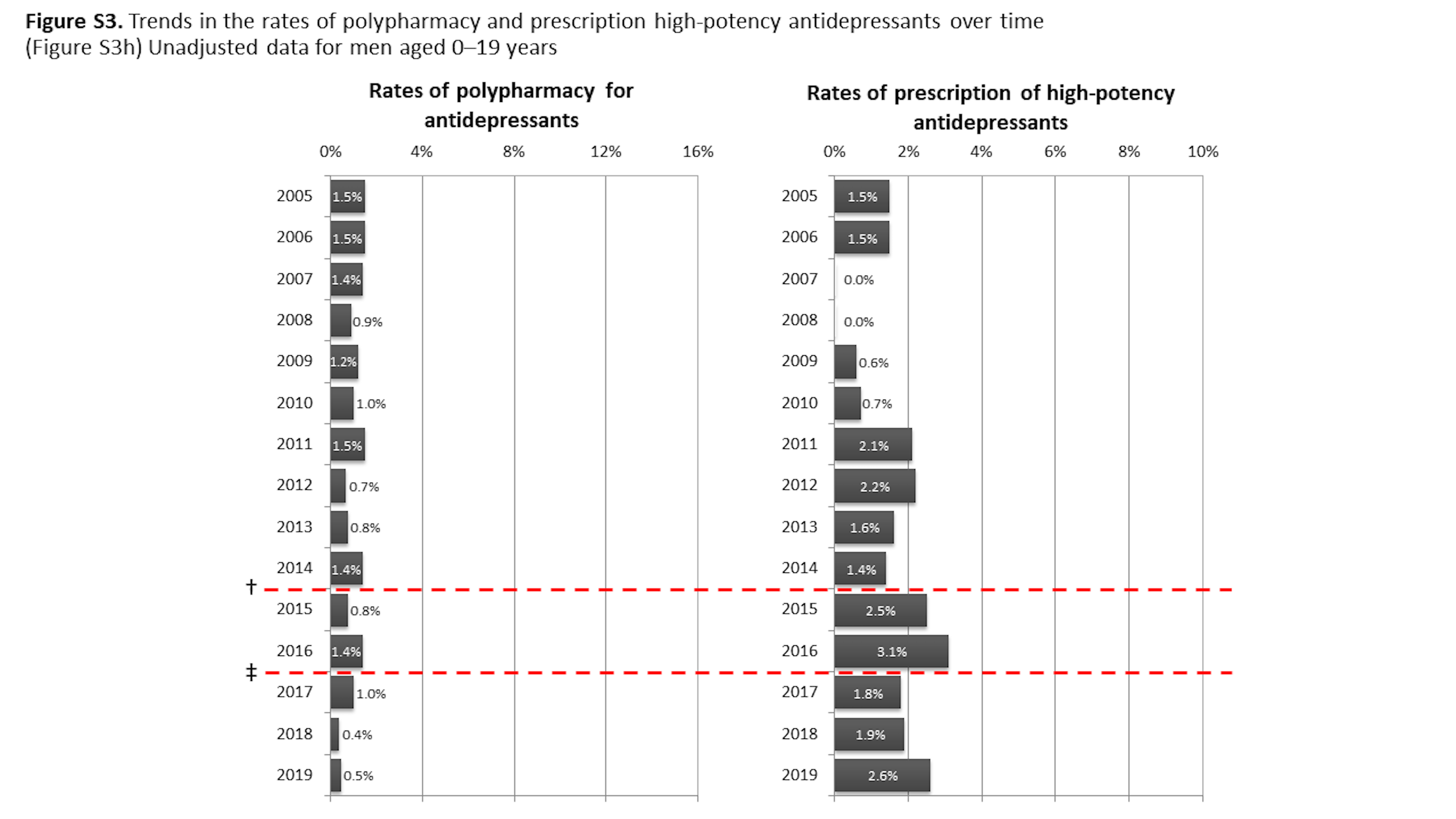

Supplement: Supplementary file 3 — Fig. S3 Trends in the rates of polypharmacy and prescription of high‐potency antidepressants over time. [file PCN-76-475-s008.zip › FigureS3h.TIF]

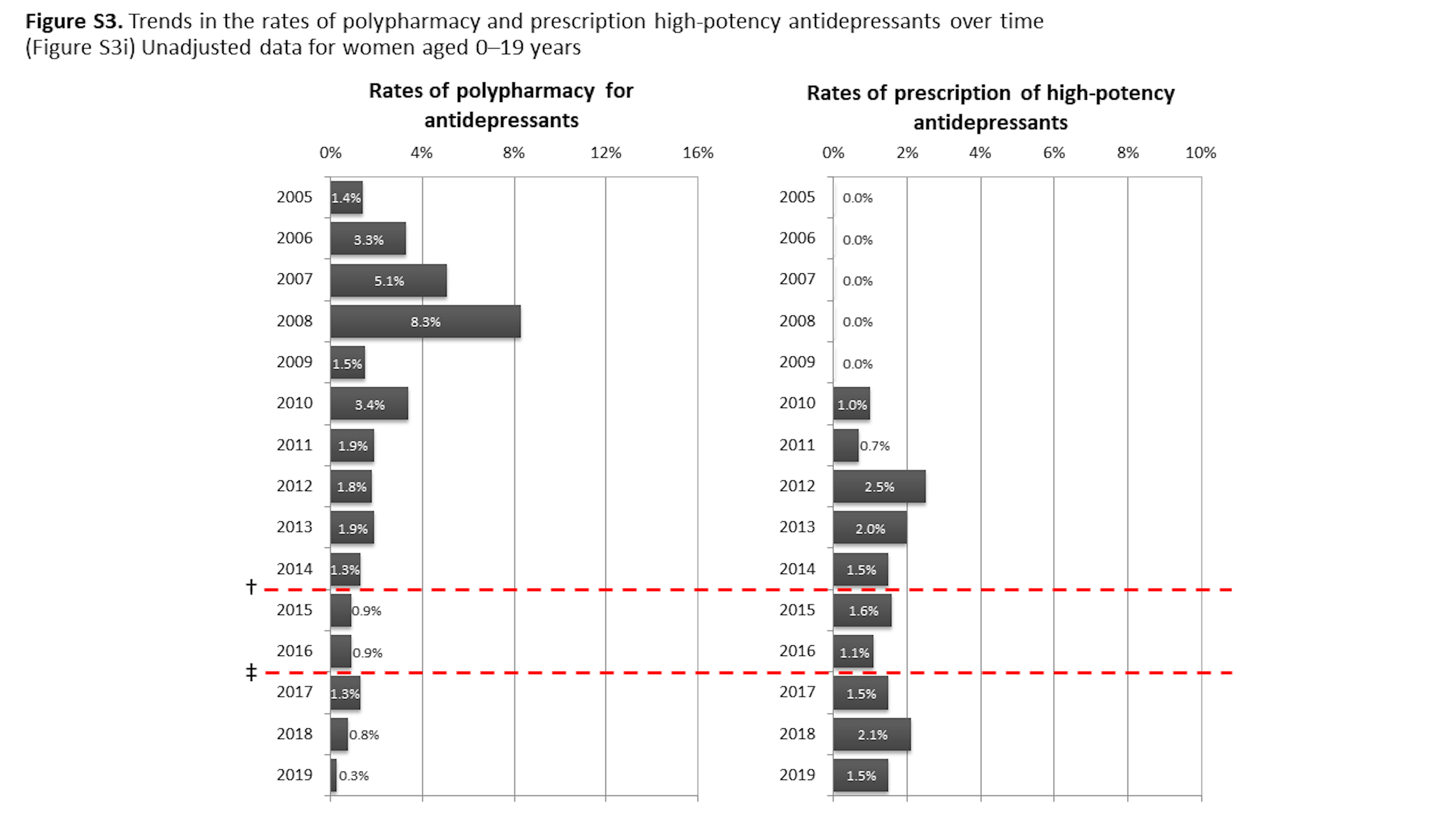

Supplement: Supplementary file 3 — Fig. S3 Trends in the rates of polypharmacy and prescription of high‐potency antidepressants over time. [file PCN-76-475-s008.zip › FigureS3i.TIF]

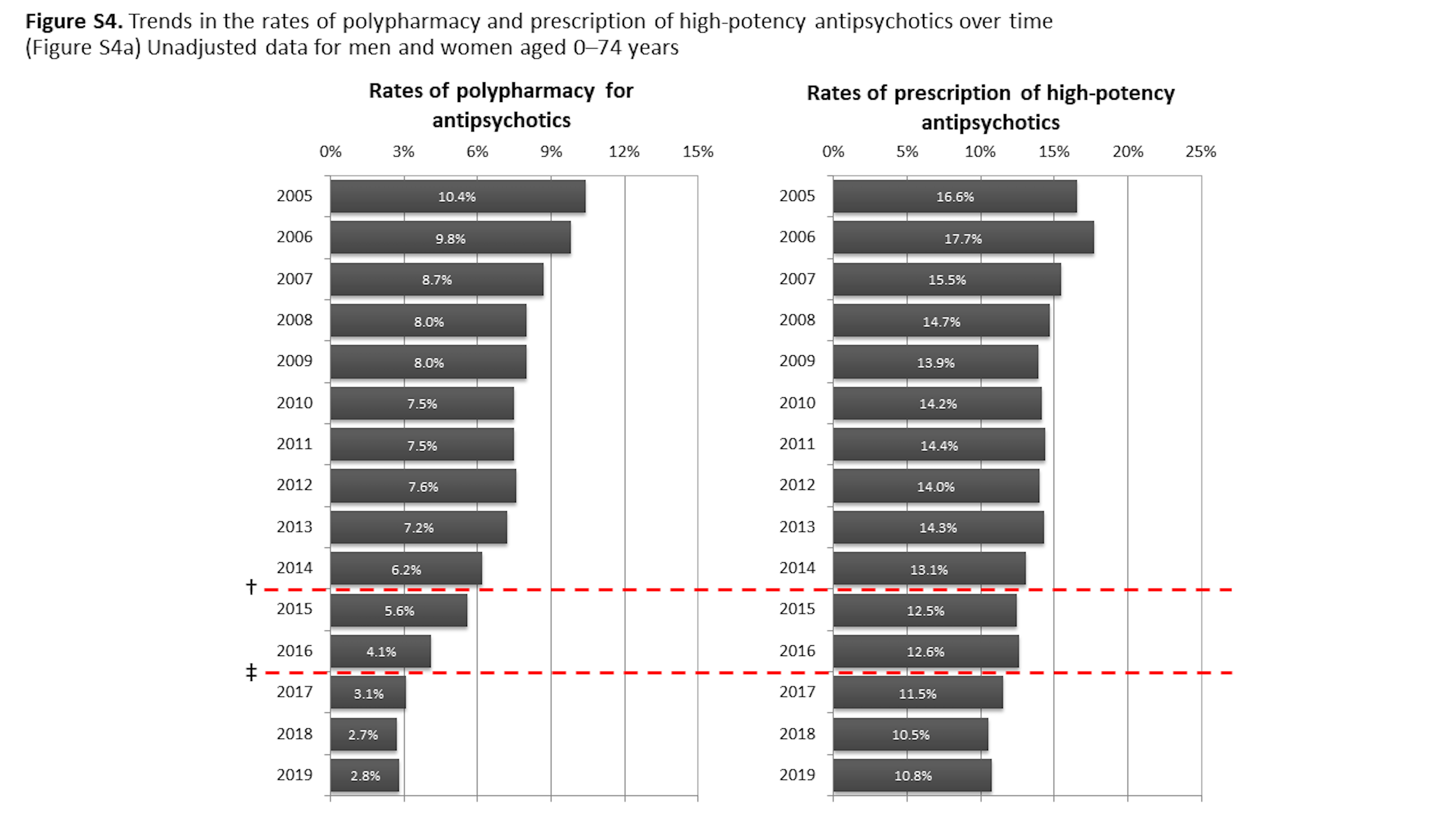

Supplement: Supplementary file 4 — Fig. S4 Trends in the rates of polypharmacy and prescription of high‐potency antipsychotics over time. [file PCN-76-475-s005.zip › FigureS4a.TIF]

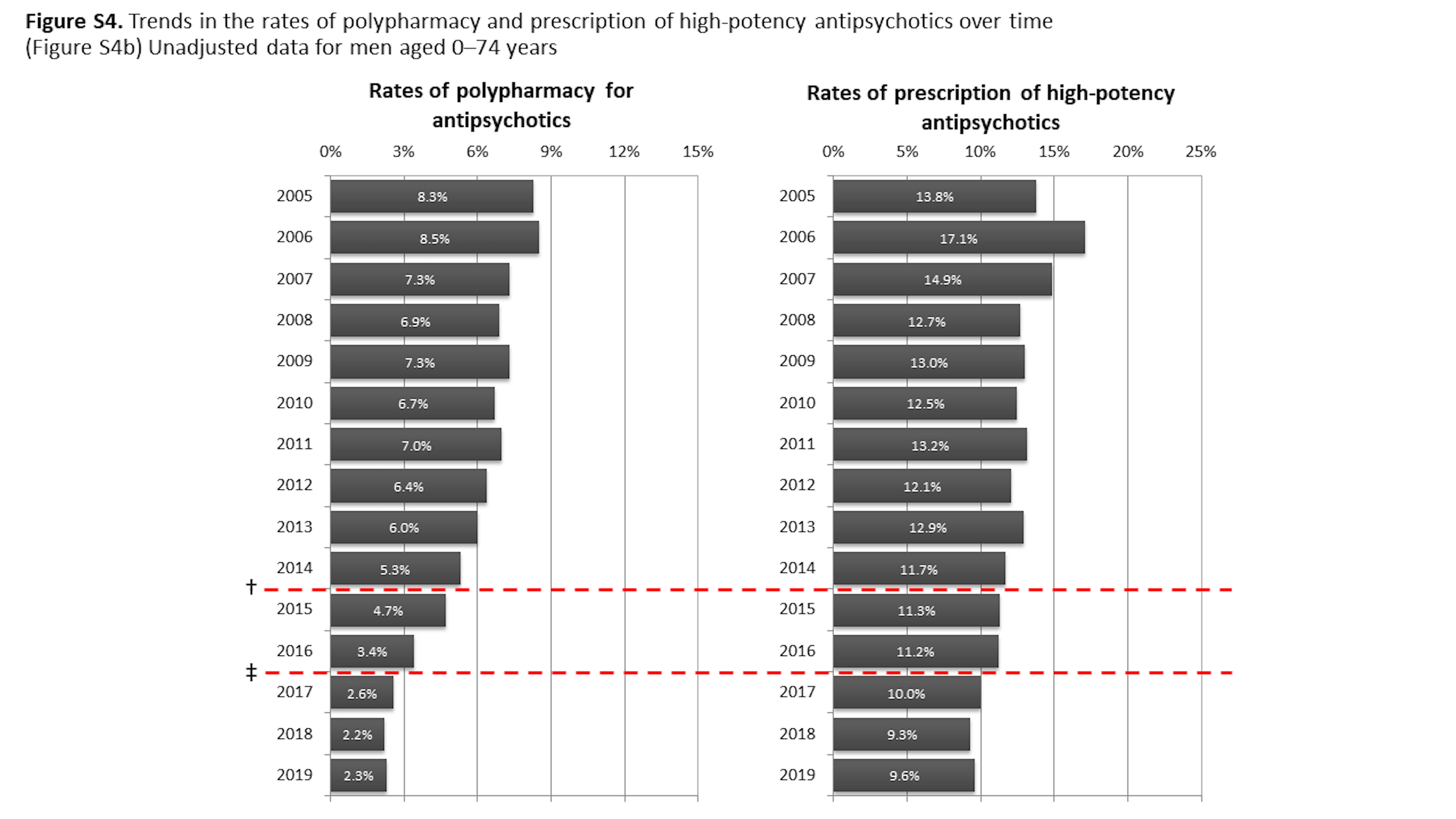

Supplement: Supplementary file 4 — Fig. S4 Trends in the rates of polypharmacy and prescription of high‐potency antipsychotics over time. [file PCN-76-475-s005.zip › FigureS4b.TIF]

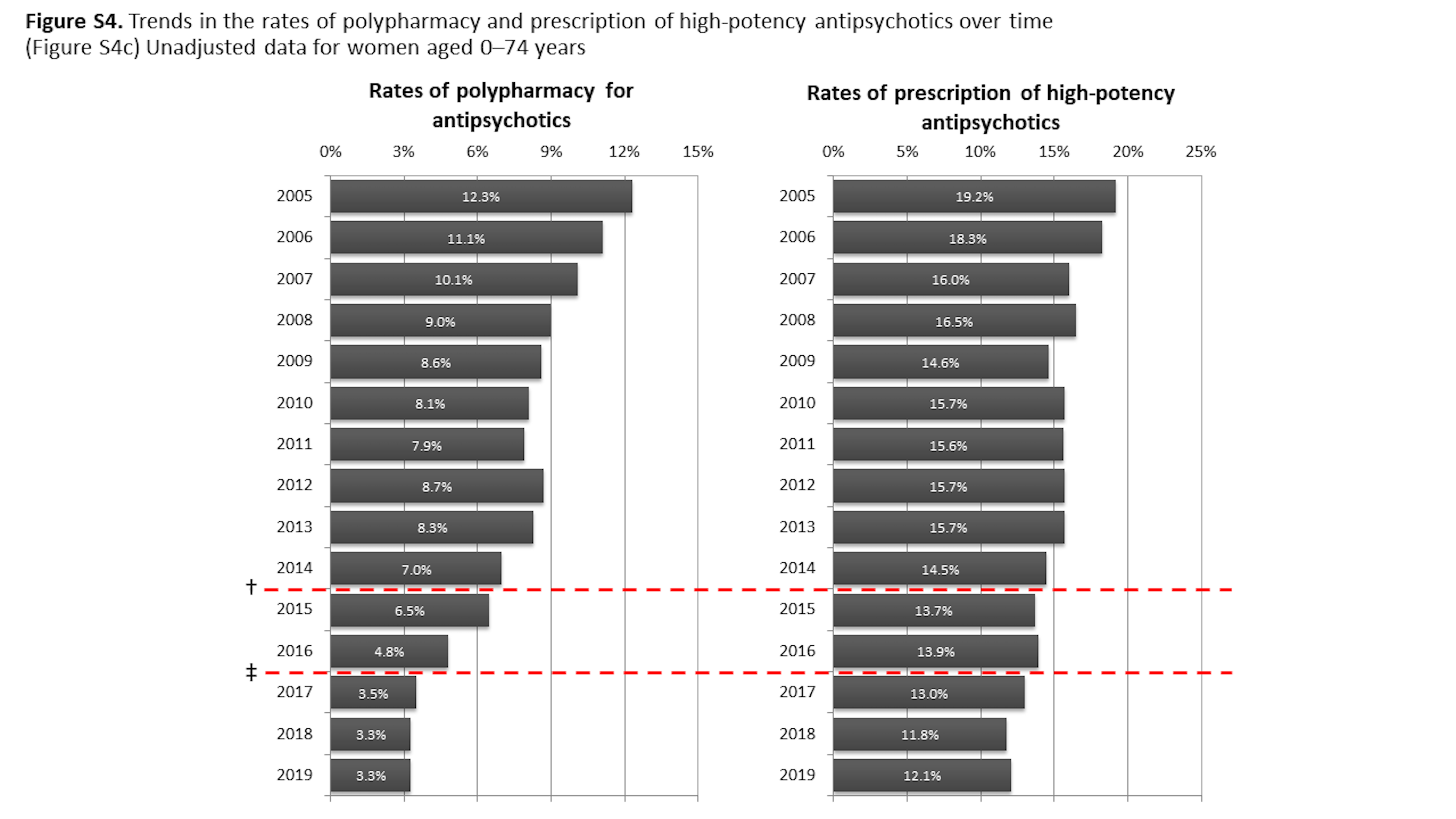

Supplement: Supplementary file 4 — Fig. S4 Trends in the rates of polypharmacy and prescription of high‐potency antipsychotics over time. [file PCN-76-475-s005.zip › FigureS4c.TIF]

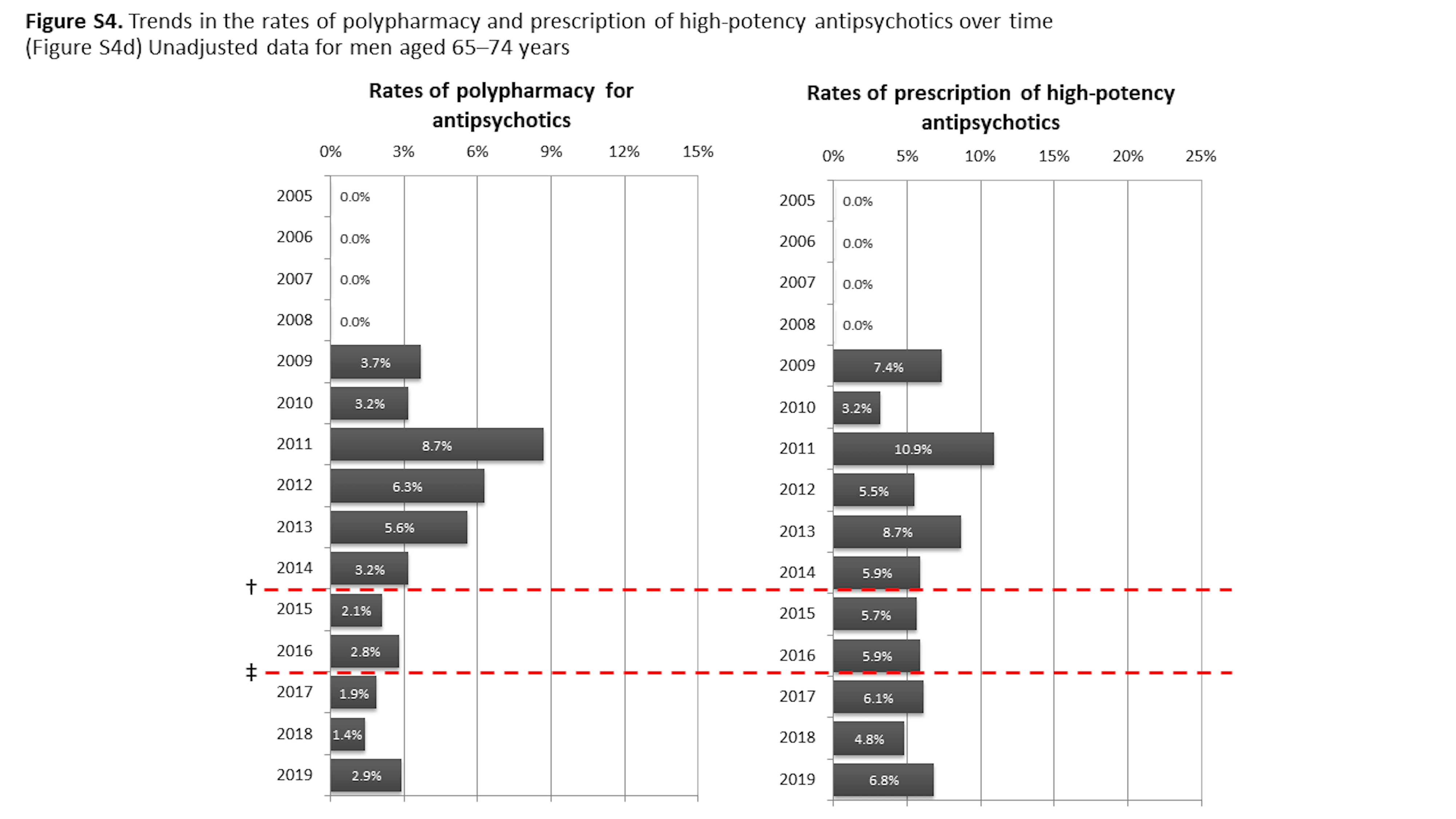

Supplement: Supplementary file 4 — Fig. S4 Trends in the rates of polypharmacy and prescription of high‐potency antipsychotics over time. [file PCN-76-475-s005.zip › FigureS4d.TIF]

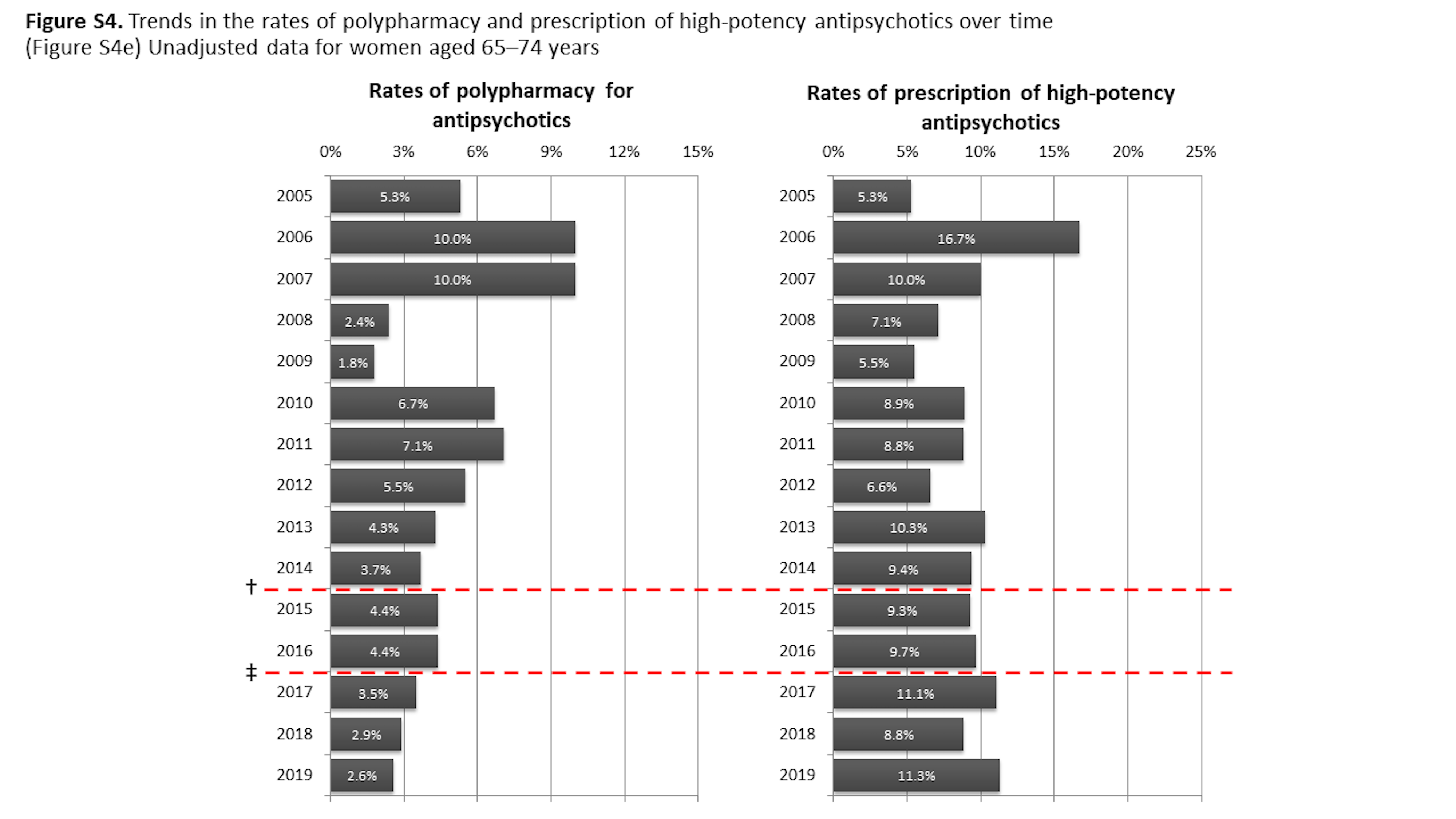

Supplement: Supplementary file 4 — Fig. S4 Trends in the rates of polypharmacy and prescription of high‐potency antipsychotics over time. [file PCN-76-475-s005.zip › FigureS4e.TIF]

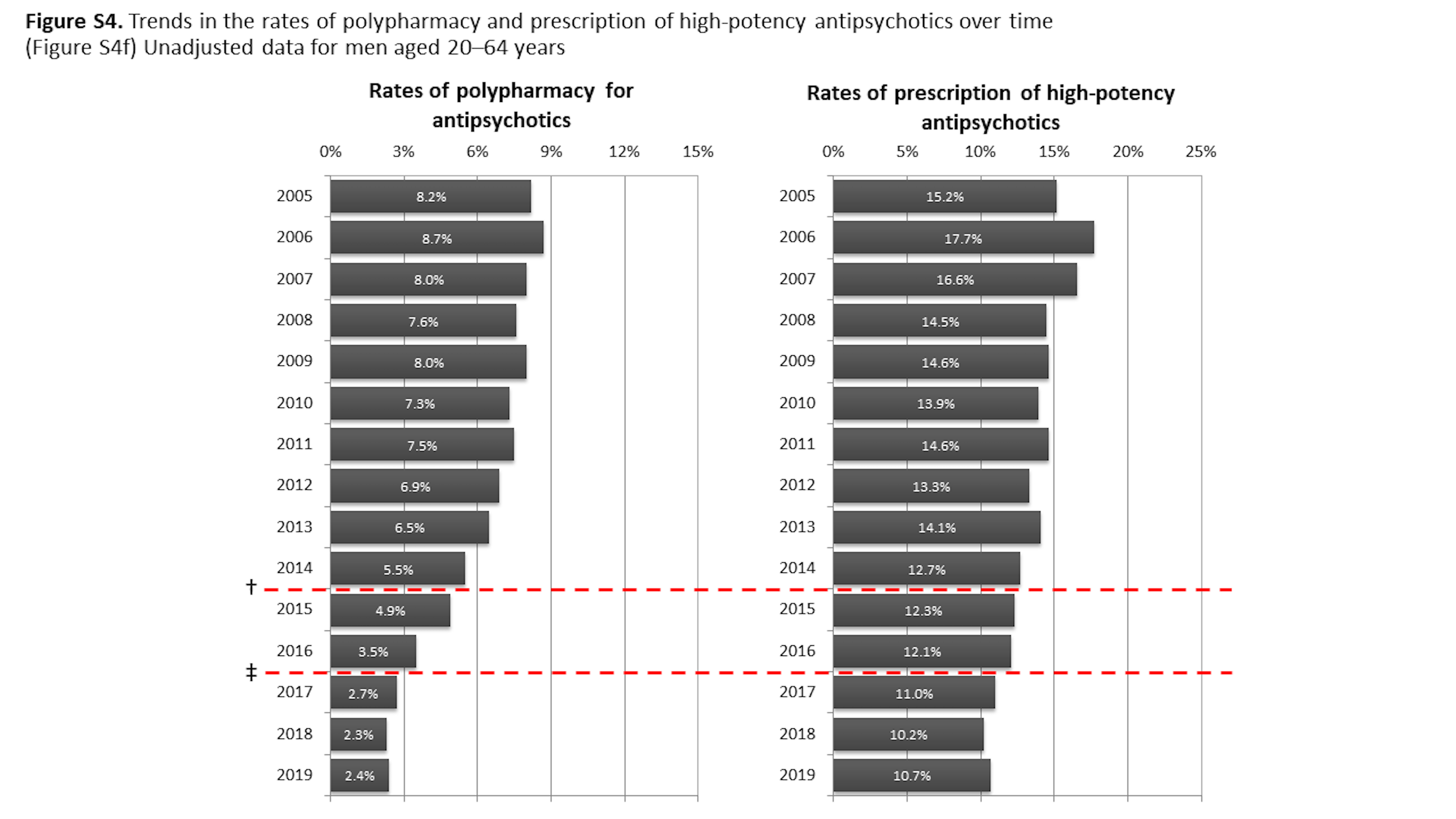

Supplement: Supplementary file 4 — Fig. S4 Trends in the rates of polypharmacy and prescription of high‐potency antipsychotics over time. [file PCN-76-475-s005.zip › FigureS4f.TIF]

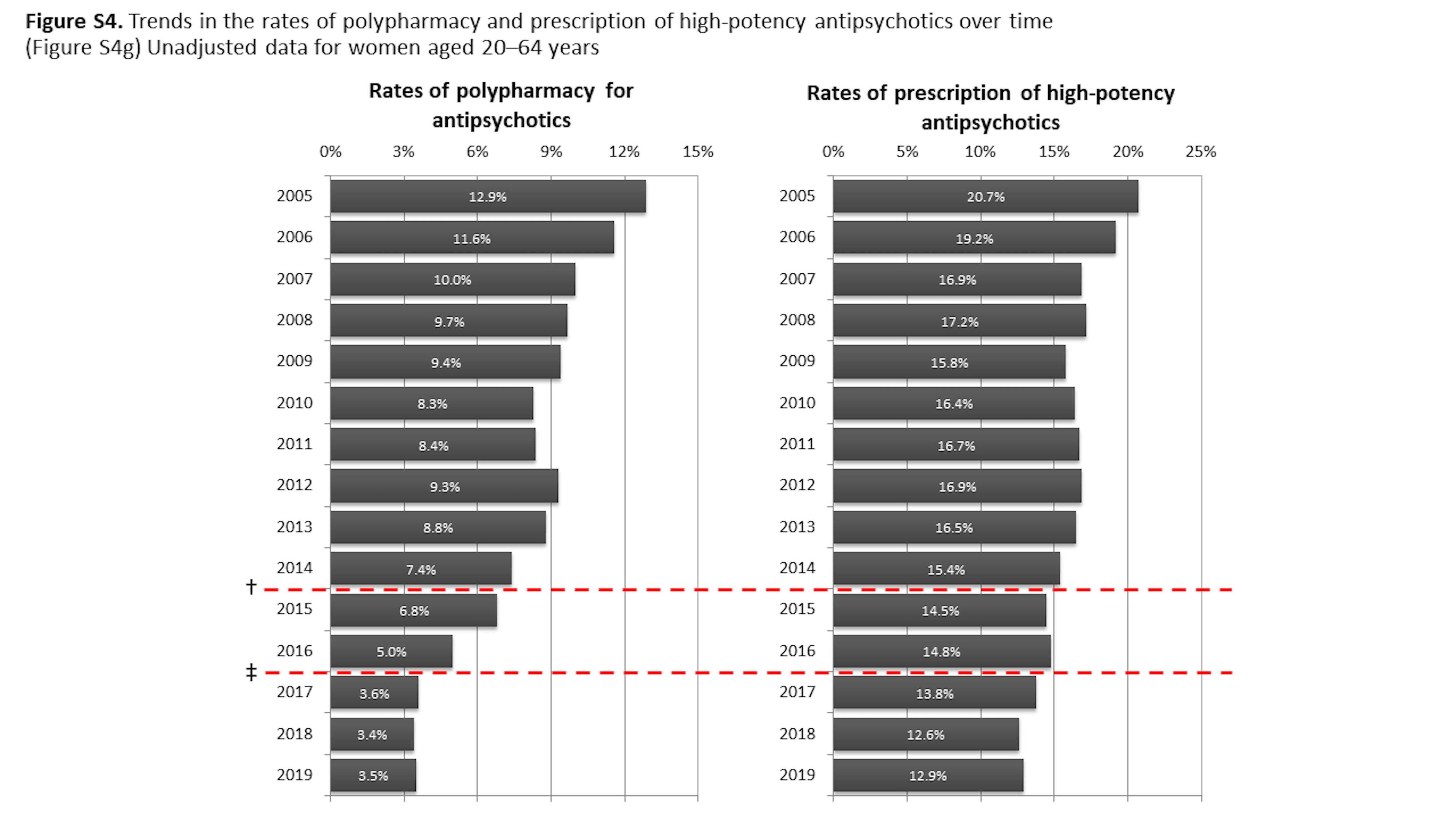

Supplement: Supplementary file 4 — Fig. S4 Trends in the rates of polypharmacy and prescription of high‐potency antipsychotics over time. [file PCN-76-475-s005.zip › FigureS4g.TIF]

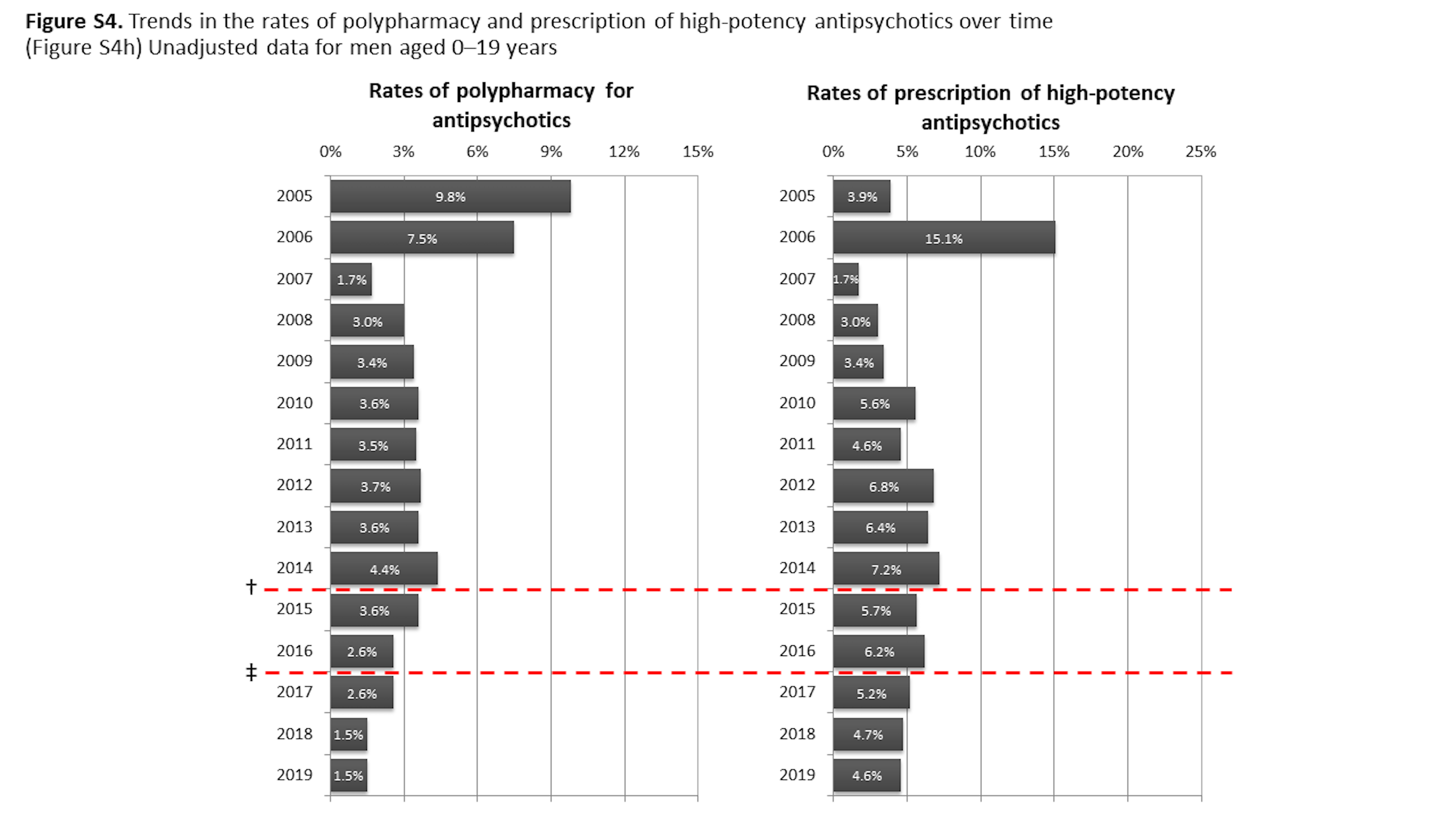

Supplement: Supplementary file 4 — Fig. S4 Trends in the rates of polypharmacy and prescription of high‐potency antipsychotics over time. [file PCN-76-475-s005.zip › FigureS4h.TIF]

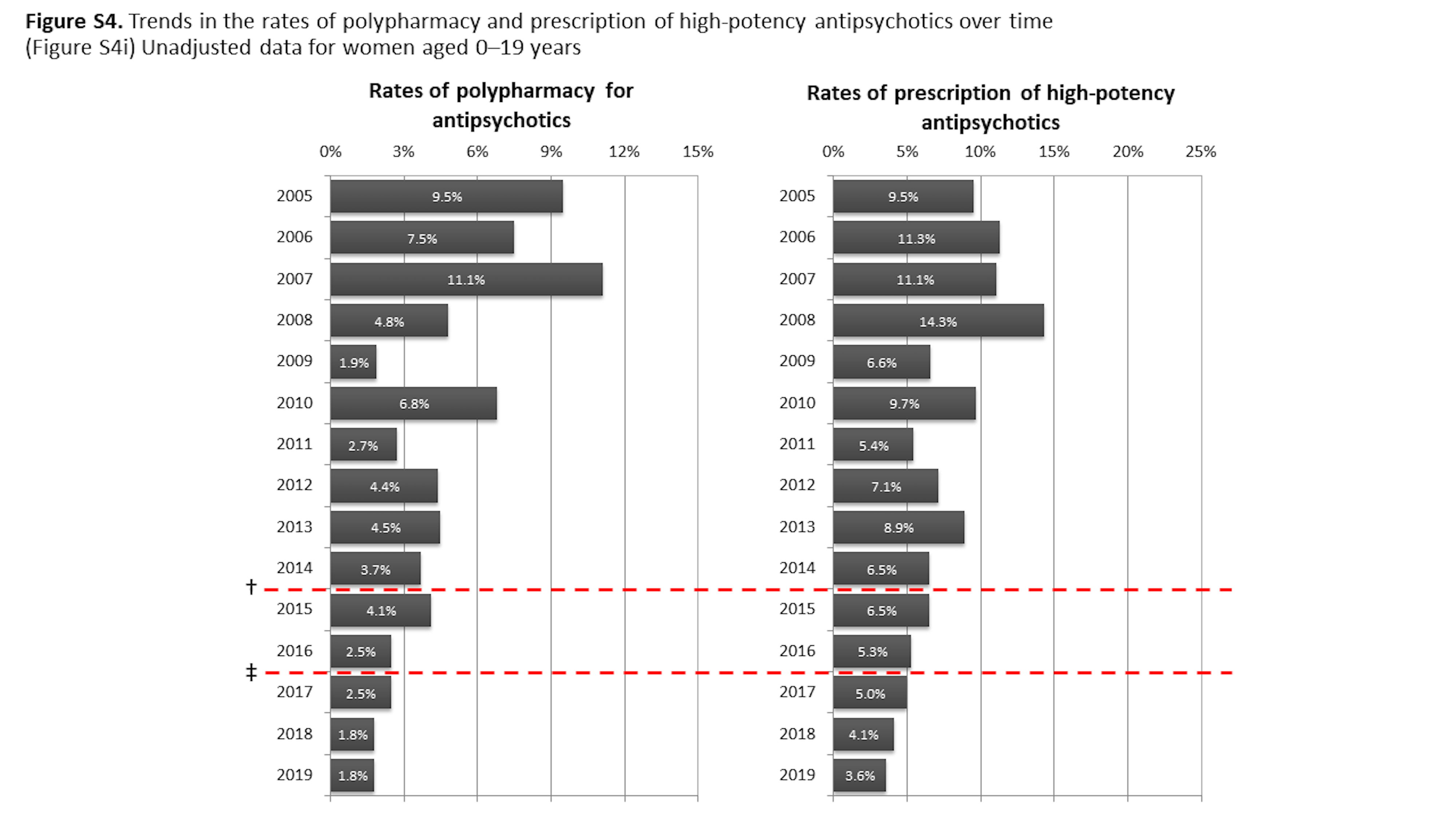

Supplement: Supplementary file 4 — Fig. S4 Trends in the rates of polypharmacy and prescription of high‐potency antipsychotics over time. [file PCN-76-475-s005.zip › FigureS4i.TIF]

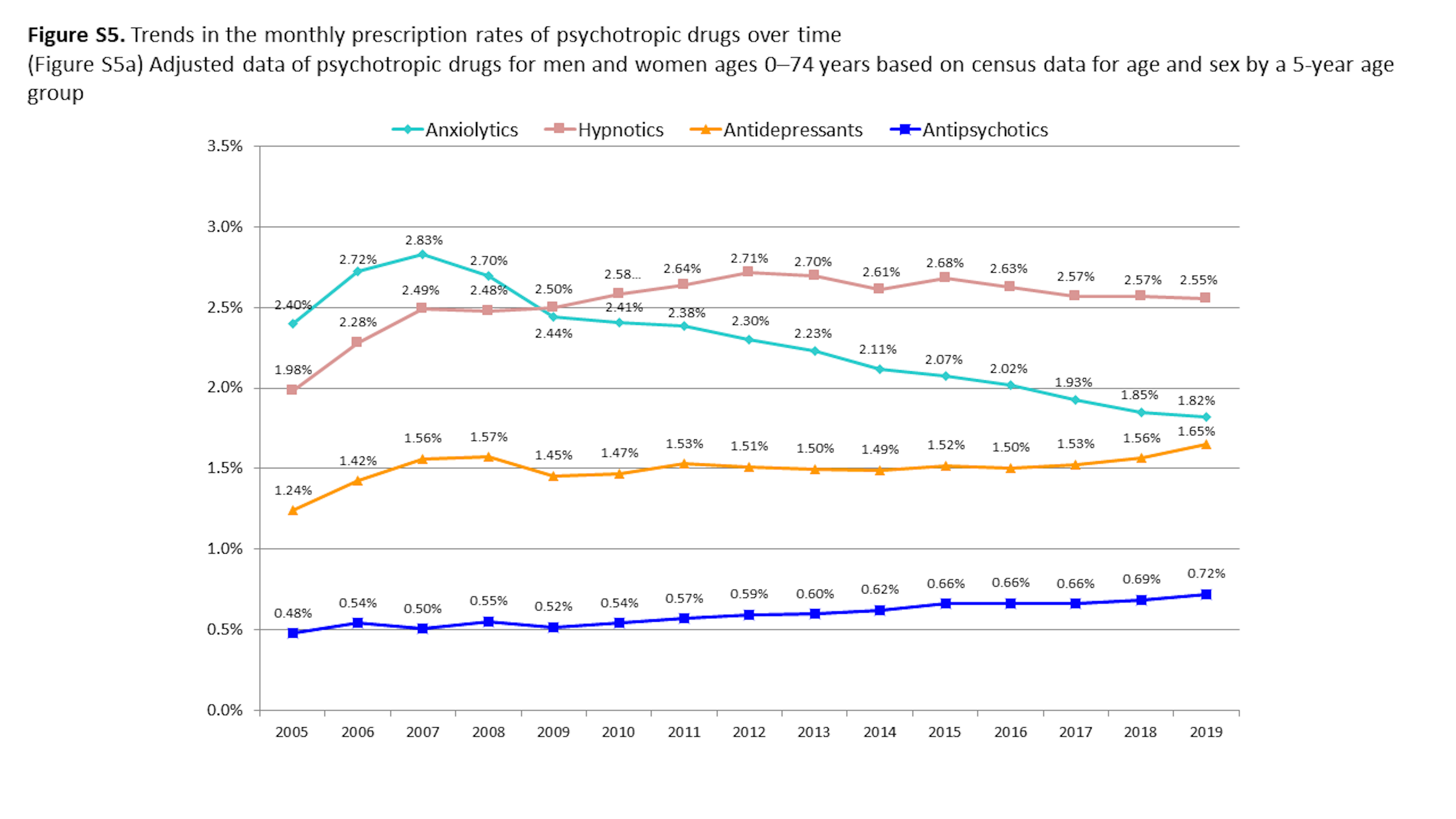

Supplement: Supplementary file 5 — Fig. S5 Trends in the monthly prescription rates of psychotropic drugs over time. [file PCN-76-475-s009.zip › FigureS5a.TIF]

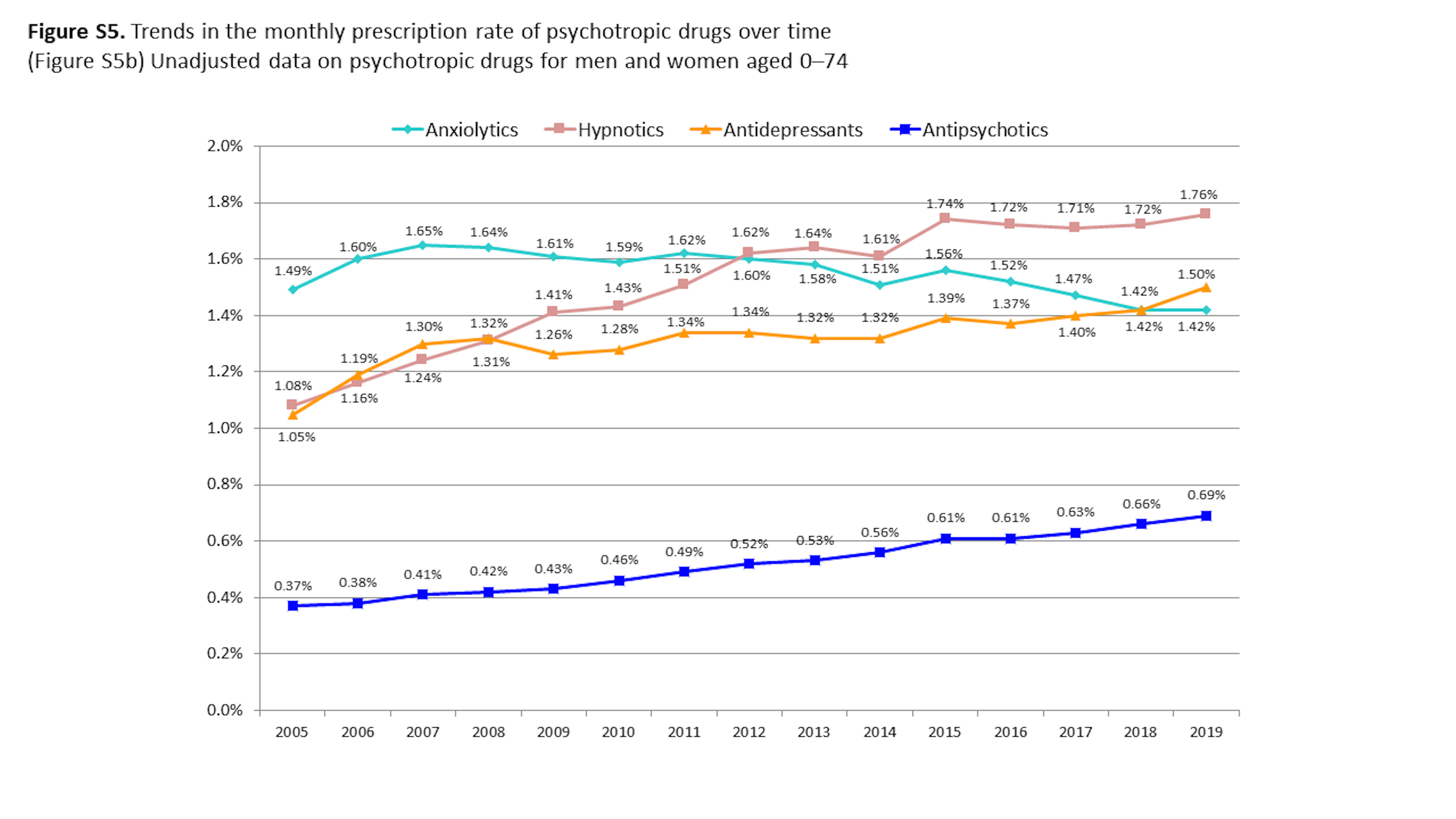

Supplement: Supplementary file 5 — Fig. S5 Trends in the monthly prescription rates of psychotropic drugs over time. [file PCN-76-475-s009.zip › FigureS5b.TIF]

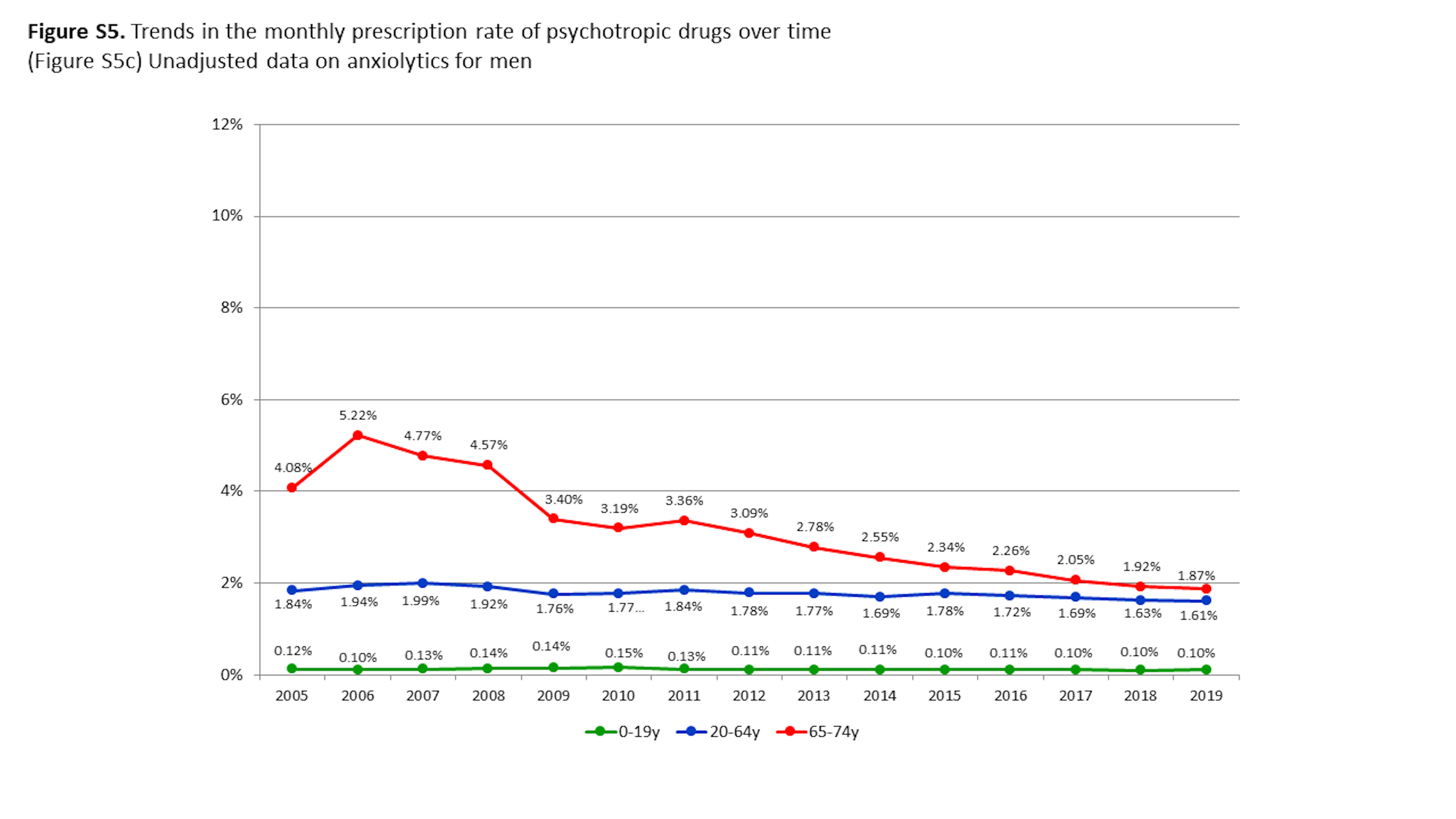

Supplement: Supplementary file 5 — Fig. S5 Trends in the monthly prescription rates of psychotropic drugs over time. [file PCN-76-475-s009.zip › FigureS5c.TIF]

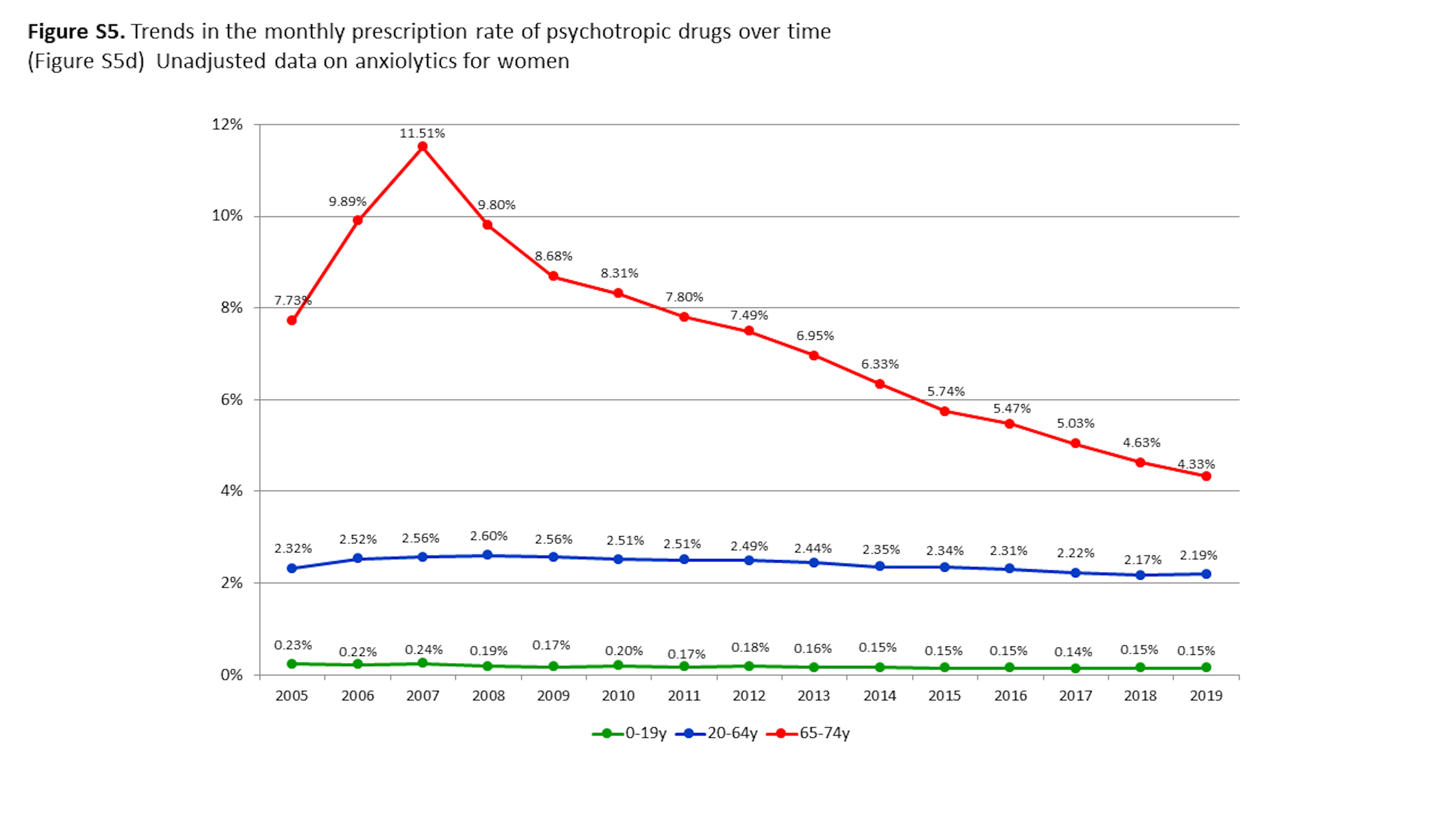

Supplement: Supplementary file 5 — Fig. S5 Trends in the monthly prescription rates of psychotropic drugs over time. [file PCN-76-475-s009.zip › FigureS5d.TIF]

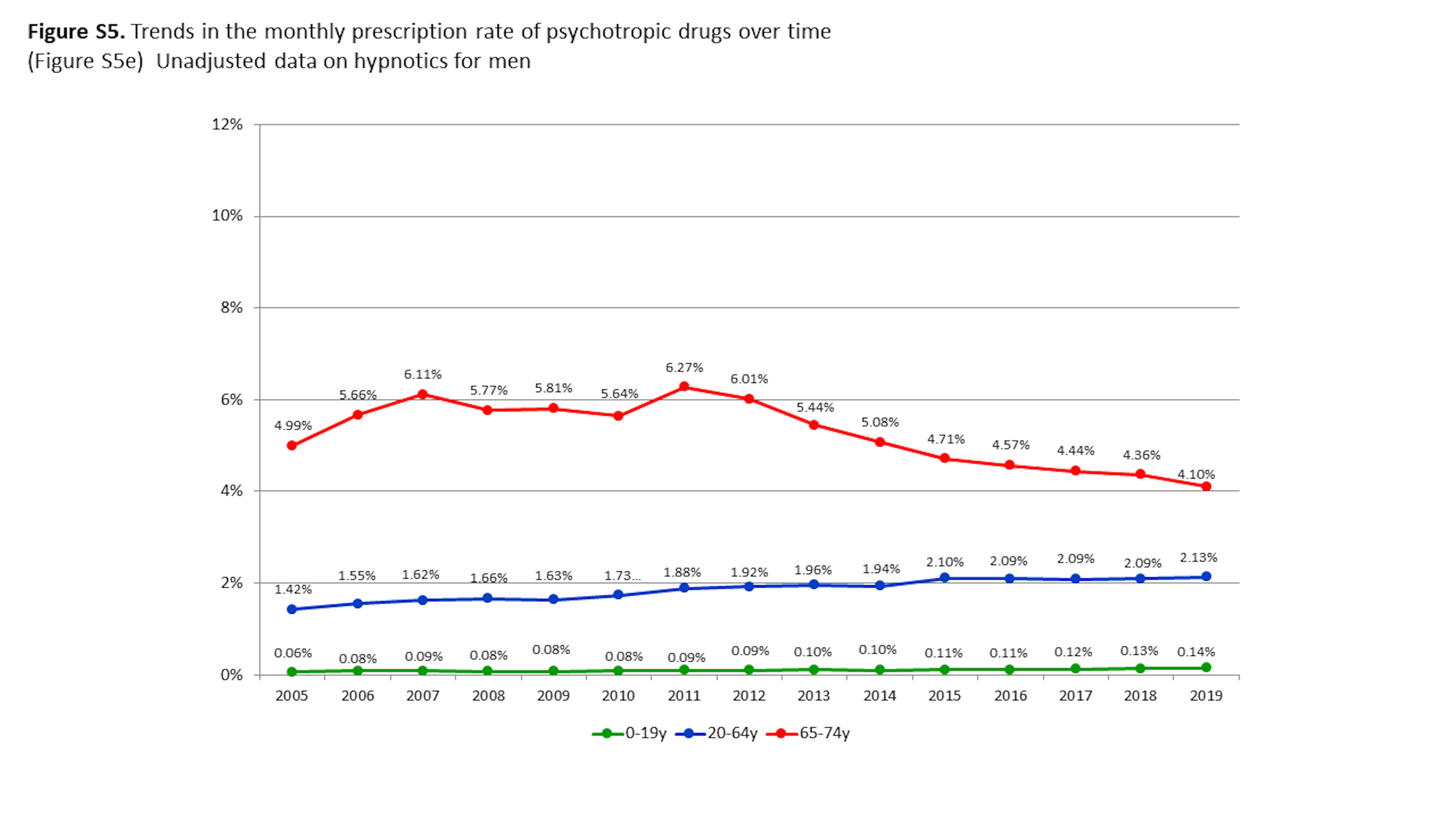

Supplement: Supplementary file 5 — Fig. S5 Trends in the monthly prescription rates of psychotropic drugs over time. [file PCN-76-475-s009.zip › FigureS5e.TIF]

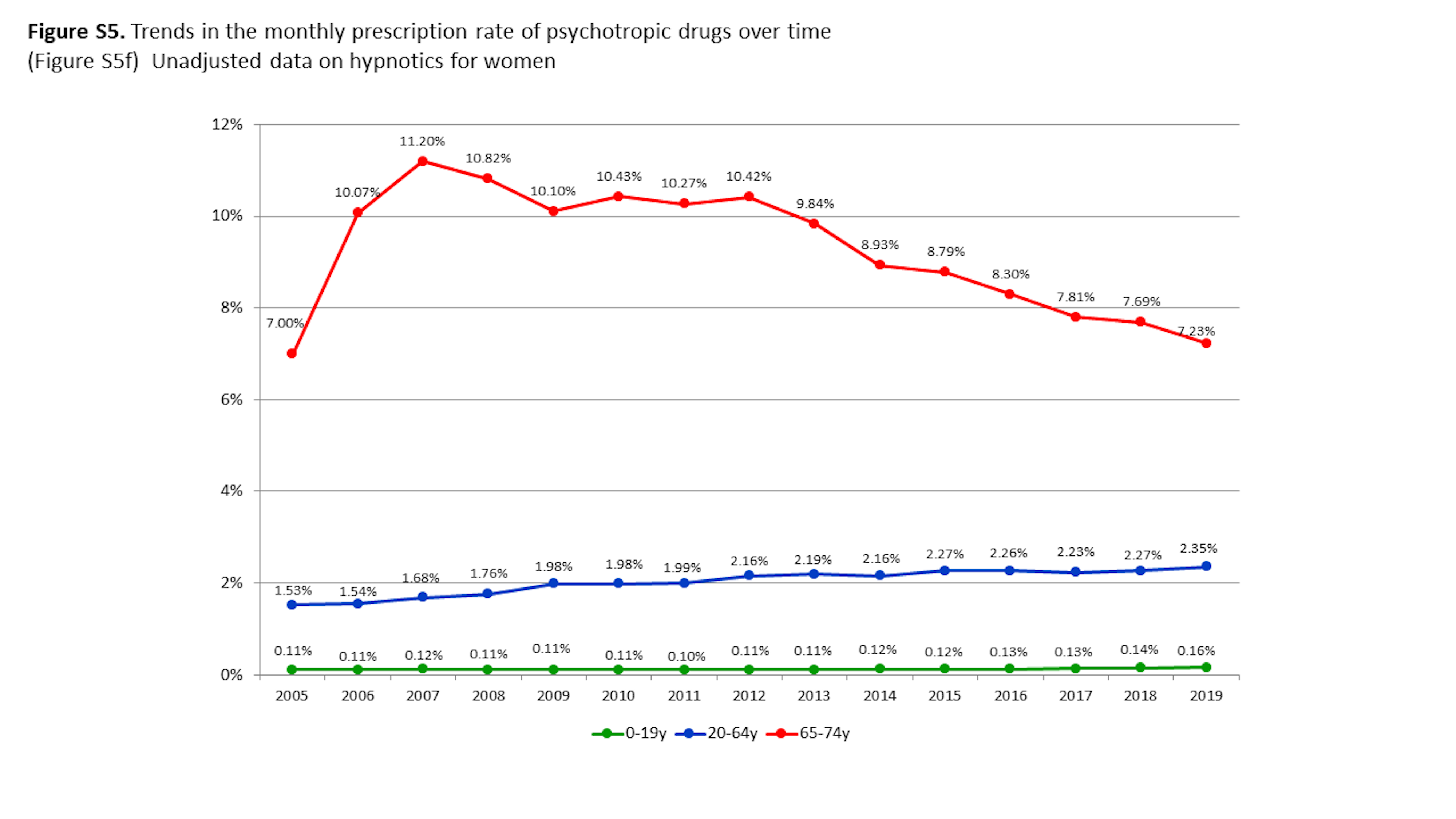

Supplement: Supplementary file 5 — Fig. S5 Trends in the monthly prescription rates of psychotropic drugs over time. [file PCN-76-475-s009.zip › FigureS5f.TIF]

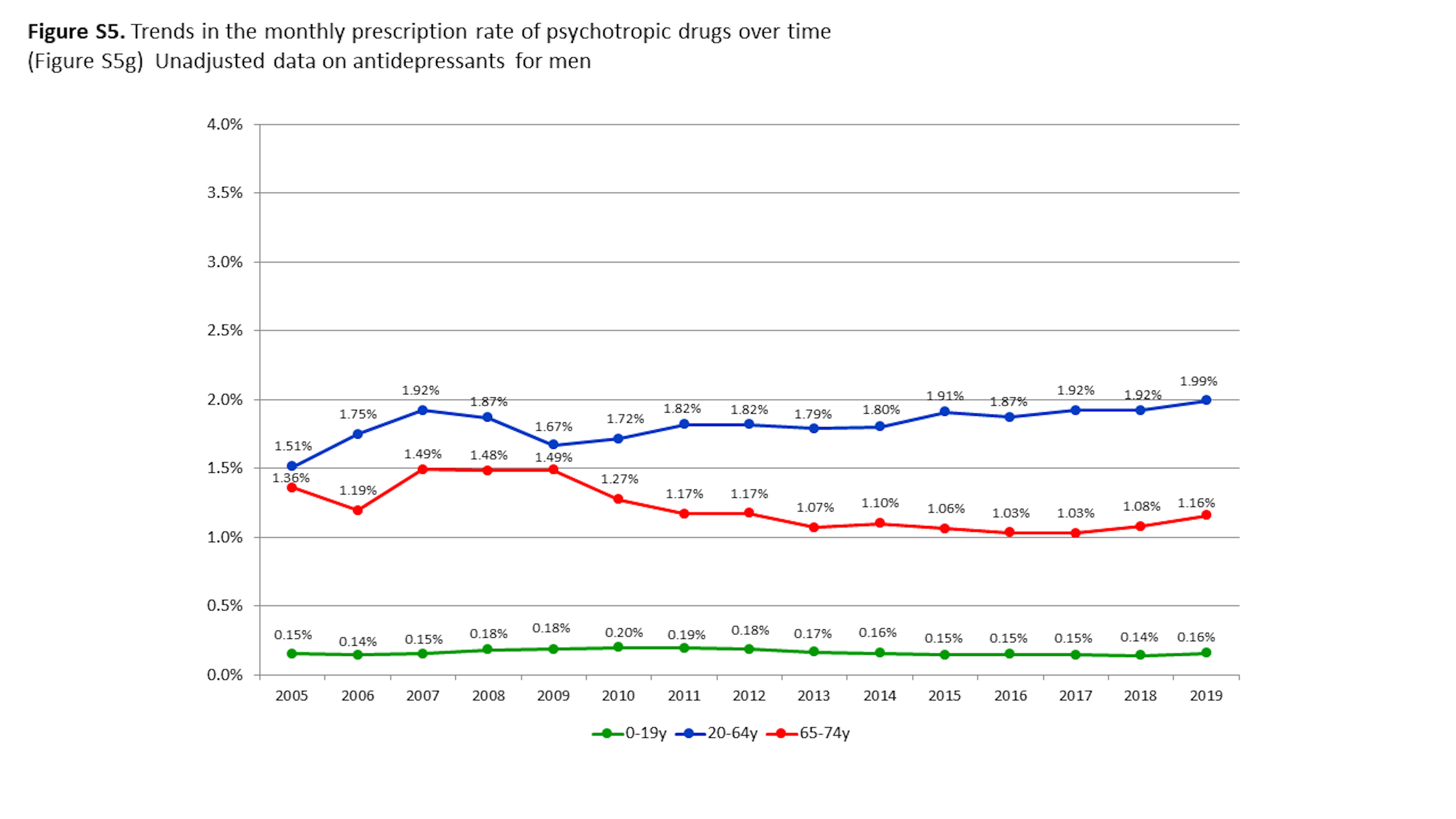

Supplement: Supplementary file 5 — Fig. S5 Trends in the monthly prescription rates of psychotropic drugs over time. [file PCN-76-475-s009.zip › FigureS5g.TIF]

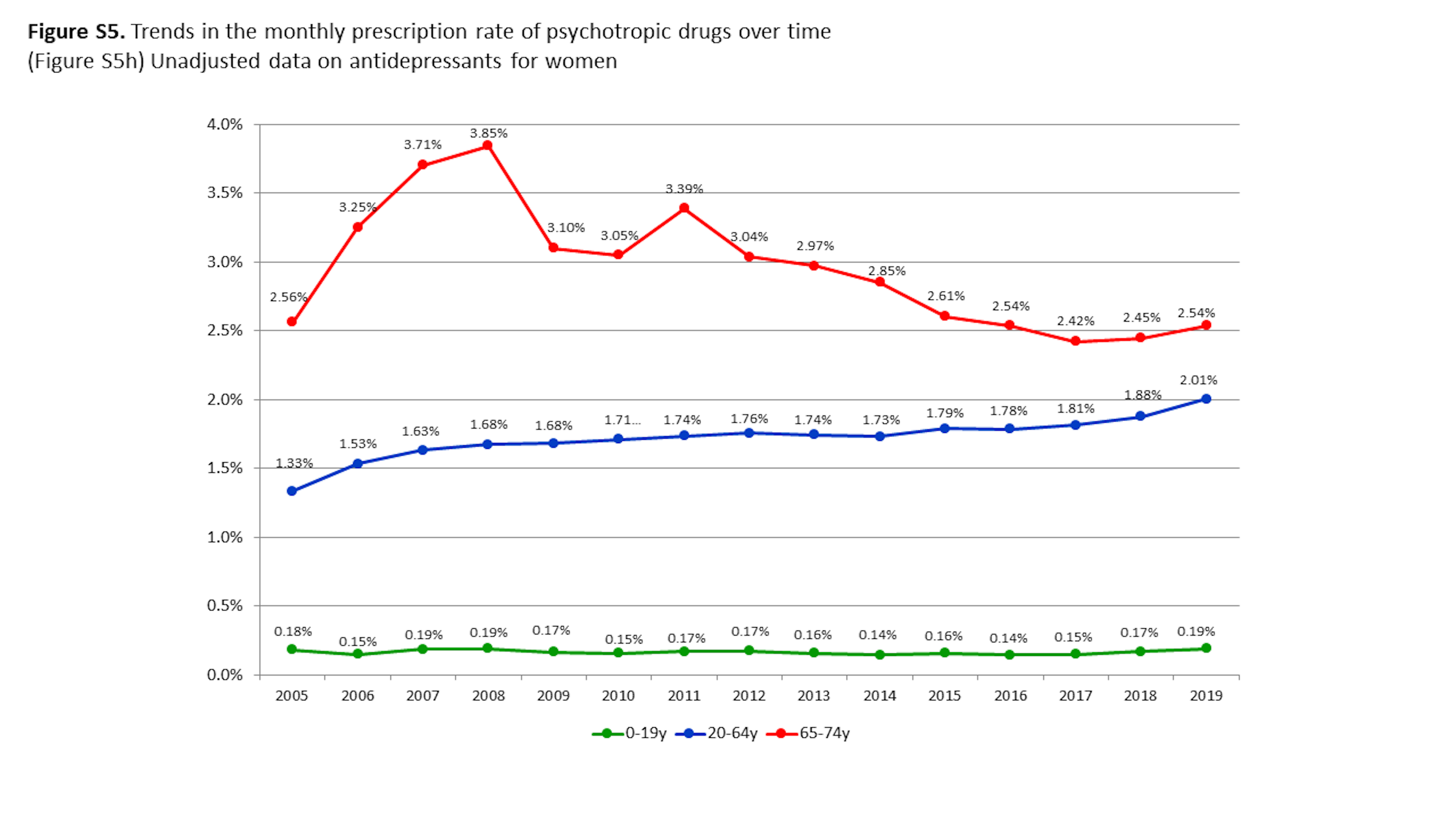

Supplement: Supplementary file 5 — Fig. S5 Trends in the monthly prescription rates of psychotropic drugs over time. [file PCN-76-475-s009.zip › FigureS5h.TIF]

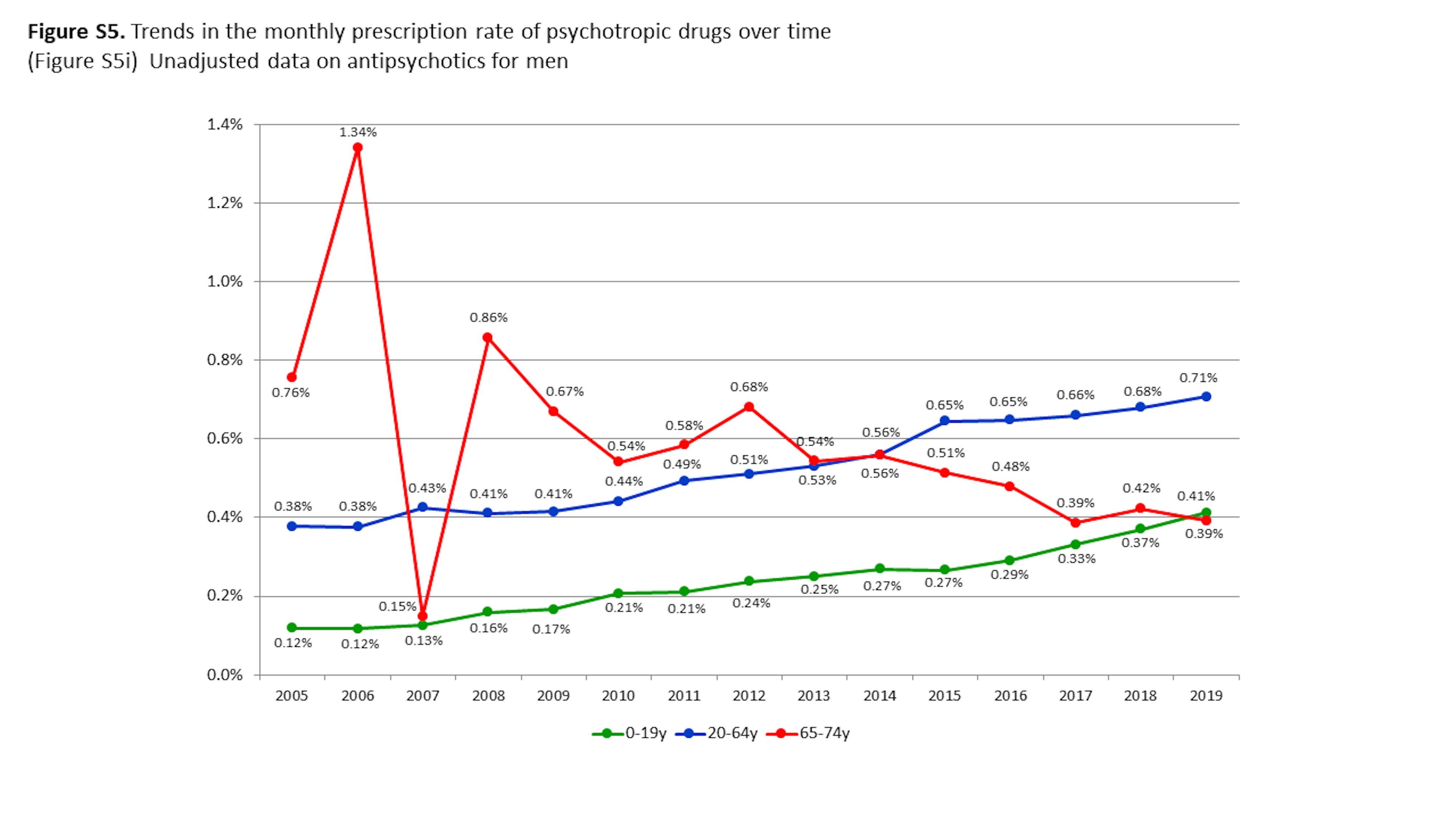

Supplement: Supplementary file 5 — Fig. S5 Trends in the monthly prescription rates of psychotropic drugs over time. [file PCN-76-475-s009.zip › FigureS5i.TIF]

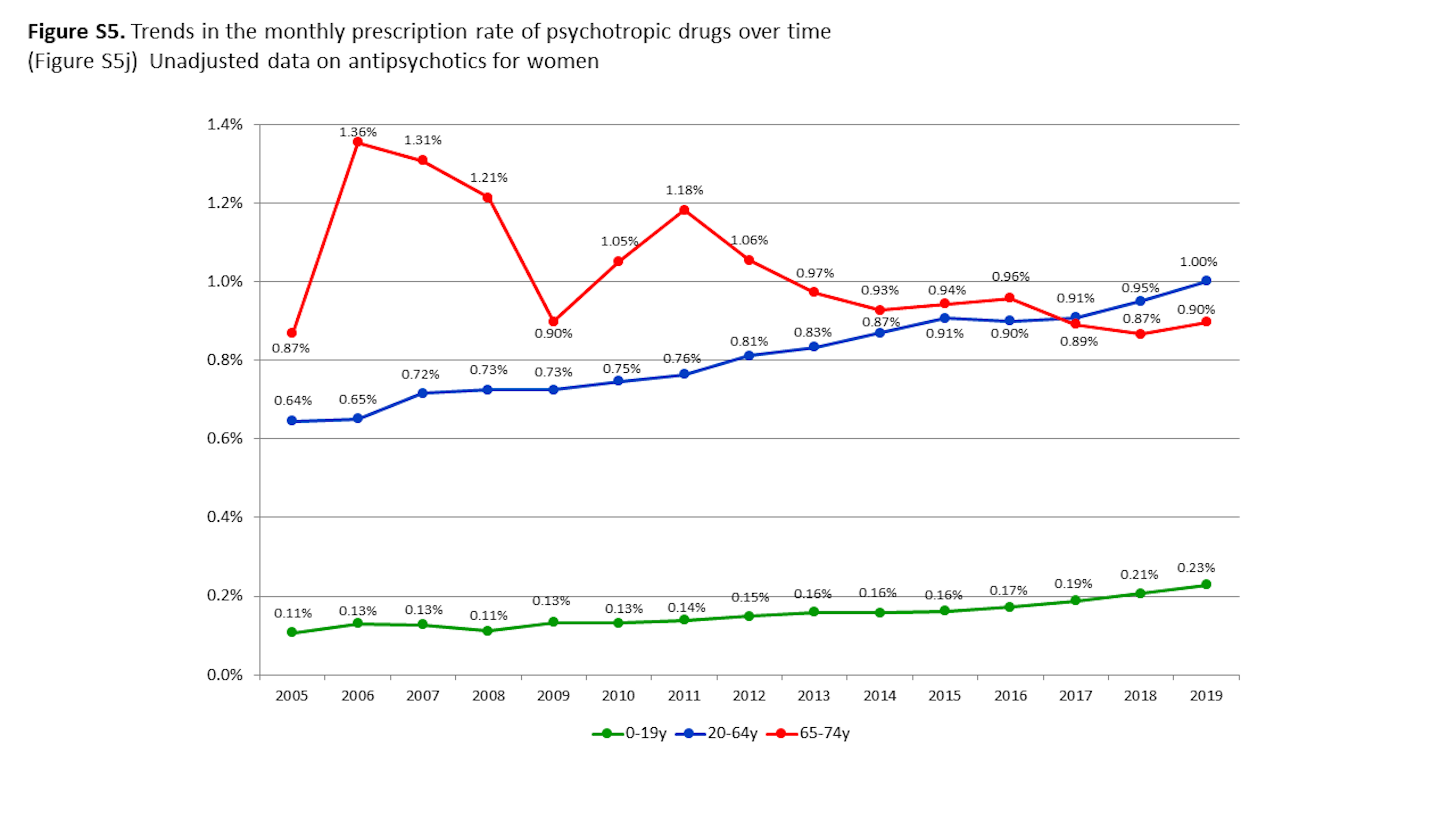

Supplement: Supplementary file 5 — Fig. S5 Trends in the monthly prescription rates of psychotropic drugs over time. [file PCN-76-475-s009.zip › FigureS5j.TIF]
