# Supplementary material for: Changes in psychotropic polypharmacy and high‐potency prescription following policy change: Findings from a large scale Japanese claims database
Source: Psychiatry Clin Neurosci. 2022 Jul 2;76(9):475–7. doi: 10.1111/pcn.13432 (PMC9546399; doi:10.1111/pcn.13432)
Supplement: Supplementary file 6 — Table S1 Details of the medical fee revisions to reduce psychotropic polypharmacy and long‐term use of benzodiazepine receptor agonists in Japan. [file PCN-76-475-s013.docx]

Table S1. Details of the revision of medical fees to reduce psychotropic polypharmacy and long-term use of benzodiazepine receptor agonists in Japan

| Year of revision | Requirements | Extent of the revision |
| --- | --- | --- |
| 2012 | Administration of three or more anxiolytics or three or more hypnotics in one prescription | Subsidy for continuous psychiatric outpatient services/ consultation fee (55 points/day)➡ Calculated as 80 out of 100 points |
| 2014 | Administration of three or more anxiolytics, three or more hypnotics, four or more antidepressants, or four or more antipsychotics | Subsidy for transportation costs and consultation fee for psychiatric treatments ➡ Incalculable Outpatient prescription charges: 68 points ➡ 30 points  Inpatient prescription charges: 42 points ➡ 20 points Medical fees: Calculated as 80 out of 100 points |
| 2016 | Administration of three or more anxiolytics, three or more hypnotics, three or more antidepressants, or three or more antipsychotics | Inpatient and outpatient prescription charges and medical fees did not change |
| 2018 | Administration of three or more anxiolytics, three or more hypnotics, three or more antidepressants, three or more antipsychotics, or four or more anxiolytics and hypnotics | Outpatient prescription charges: 68 points ➡ 28 points Inpatient prescription charges: 42 points ➡ 18 points Medical fees: Calculated as 80 out of 100 points |
|  | Prescription of benzodiazepines at the same dose and administration for 12 or more consecutive months or more for symptoms of anxiety or insomnia | Outpatient prescription charges: 68 points ➡ 40 points Inpatient prescription charges: 42 points ➡ 29 points |
|  | Patients with psychotropic polypharmacy, anxiety, or insomnia symptoms after the latest prescription of psychotropics or those prescribed benzodiazepines at the same dose and administration for 12 or more consecutive months for symptoms of anxiety or insomnia for whom pharmacists (pharmacists or nurses regarding inpatient prescription charges) were requested to examine changes in symptoms after dose reduction | Outpatient prescription charges: 68 points ➡ 80 points Inpatient prescription charges: 42 points ➡ 54 points |

Note: 1 point=10 Japanese yen
