# Supplementary material for: Changes in psychotropic polypharmacy and high‐potency prescription following policy change: Findings from a large scale Japanese claims database
Source: Psychiatry Clin Neurosci. 2022 Jul 2;76(9):475–7. doi: 10.1111/pcn.13432 (PMC9546399; doi:10.1111/pcn.13432)
Supplement: Supplementary file 7 — Table S2 Demographic data of the subscribers to the health insurance service. [file PCN-76-475-s011.docx]

Table S2. Demographic data of subscribers to the health insurance service

|  | 2005  (N=259053) | | 2007  (N=283715) | | 2009  (N=584444) | | 2011  (N=1177635) | | 2013  (N=2568626) | | 2015  (N=3721405) | | 2017  (N=4813533) | | 2019  (N=4768608) | |
| --- | --- | --- | --- | --- | --- | --- | --- | --- | --- | --- | --- | --- | --- | --- | --- | --- |
|  | Male | Female | Male | Female | Male | Female | Male | Female | Male | Female | Male | Female | Male | Female | Male | Female |
| Total | 58.0% | 42.0% | 58.6% | 41.4% | 56.5% | 43.5% | 55.9% | 44.1% | 55.8% | 44.2% | 55.5% | 44.5% | 55.3% | 44.7% | 55.8% | 44.2% |
| 0–4 y | 4.5% | 4.2% | 4.3% | 4.1% | 3.7% | 3.5% | 3.8% | 3.6% | 3.7% | 3.5% | 3.4% | 3.3% | 3.3% | 3.1% | 3.3% | 3.1% |
| 5–9 y | 4.5% | 4.2% | 4.3% | 4.0% | 3.8% | 3.6% | 3.7% | 3.5% | 3.5% | 3.4% | 3.4% | 3.2% | 3.4% | 3.2% | 3.4% | 3.2% |
| 10–14 y | 3.9% | 3.6% | 3.9% | 3.6% | 3.7% | 3.5% | 3.7% | 3.5% | 3.7% | 3.5% | 3.5% | 3.3% | 3.4% | 3.2% | 3.4% | 3.2% |
| 15–19 y | 3.9% | 3.1% | 4.4% | 3.2% | 3.8% | 3.1% | 3.6% | 3.1% | 3.7% | 3.2% | 3.8% | 3.3% | 3.8% | 3.4% | 3.9% | 3.4% |
| 20–24 y | 4.4% | 2.4% | 5.2% | 2.7% | 4.6% | 2.9% | 4.4% | 2.8% | 4.3% | 2.6% | 4.0% | 2.5% | 4.1% | 2.8% | 4.3% | 2.9% |
| 25–29 y | 5.3% | 2.9% | 5.5% | 2.8% | 5.0% | 3.1% | 5.0% | 3.2% | 4.7% | 2.8% | 4.4% | 2.6% | 4.2% | 2.6% | 4.2% | 2.5% |
| 30–34 y | 7.0% | 5.0% | 6.5% | 4.3% | 5.4% | 4.0% | 5.2% | 3.9% | 4.9% | 3.5% | 4.5% | 3.3% | 4.5% | 3.3% | 4.4% | 3.0% |
| 35–39 y | 6.9% | 4.8% | 6.5% | 4.9% | 6.1% | 5.1% | 5.9% | 4.9% | 5.3% | 4.4% | 4.9% | 4.0% | 4.6% | 3.8% | 4.6% | 3.5% |
| 40–44 y | 6.0% | 3.7% | 6.1% | 3.8% | 5.8% | 4.3% | 5.7% | 4.6% | 5.6% | 4.8% | 5.6% | 4.9% | 5.4% | 4.7% | 5.0% | 4.2% |
| 45–49 y | 4.0% | 2.5% | 4.3% | 2.6% | 4.8% | 3.2% | 4.9% | 3.6% | 5.1% | 3.9% | 5.1% | 4.3% | 5.4% | 4.7% | 5.5% | 4.7% |
| 50–54 y | 3.4% | 2.4% | 3.2% | 2.1% | 3.5% | 2.5% | 3.7% | 2.7% | 4.3% | 3.2% | 4.8% | 3.6% | 5.0% | 3.8% | 5.1% | 4.0% |
| 55–59 y | 3.1% | 1.9% | 3.1% | 1.9% | 3.4% | 2.4% | 3.1% | 2.4% | 3.3% | 2.5% | 3.8% | 2.9% | 3.9% | 2.9% | 4.3% | 3.1% |
| 60–64 y | 1.0% | 0.6% | 1.1% | 0.6% | 2.1% | 1.3% | 2.5% | 1.6% | 2.6% | 1.8% | 3.0% | 2.0% | 2.8% | 1.9% | 2.9% | 1.9% |
| 65–69 y | 0.1% | 0.4% | 0.1% | 0.4% | 0.5% | 0.6% | 0.5% | 0.5% | 0.8% | 0.7% | 1.1% | 0.9% | 1.1% | 0.9% | 1.1% | 0.8% |
| 70–74 y | 0.1% | 0.4% | 0.1% | 0.4% | 0.2% | 0.5% | 0.2% | 0.4% | 0.4% | 0.4% | 0.4% | 0.4% | 0.3% | 0.4% | 0.4% | 0.4% |
