# Supplementary material for: Changes in psychotropic polypharmacy and high‐potency prescription following policy change: Findings from a large scale Japanese claims database
Source: Psychiatry Clin Neurosci. 2022 Jul 2;76(9):475–7. doi: 10.1111/pcn.13432 (PMC9546399; doi:10.1111/pcn.13432)
Supplement: Supplementary file 8 — Table S3 List of the psychotropic drugs that can be prescribed in Japan and their potencies. [file PCN-76-475-s006.docx]

Table S3. List of psychotropic drugs that can be prescribed in Japan and their potencies

**Hypnotics**

| Generic name | Dose equivalence (mg/day) |
| --- | --- |
| Amobarbital | 50 |
| Barbital | 75 |
| Bromovalerylurea^†^ | 500 |
| Brotizolam | 0.25 |
| Butoctamide^†^ | 500 |
| Chloral hydrate | 250 |
| Estazolam | 2 |
| Eszopiclone | 2.5 |
| Etizolam | 1.5* |
| Flunitrazepam | 1 |
| Flurazepam | 15 |
| Haloxazolam^†^ | 5 |
| Lormetazepam | 1 |
| Nimetazepam | 5 |
| Nitrazepam | 5 |
| Passiflora extract^†^ | 100 |
| Pentobarbital calcium | 50 |
| Quazepam | 15 |
| Ramelteon | 8 |
| Rilmazafone^†^ | 2 |
| Suvorexant | 20 |
| Triazolam | 0.25 |
| Zolpidem | 10 |
| Zopiclone | 7.5 |

^†^Hynotics not indexed in the Anatomical Therapeutic Chemical classification 2021

**Anxiolytics**

| Generic name | Dose equivalence (mg/day) |
| --- | --- |
| Alprazolam | 0.8 |
| Bromazepam | 2.5 |
| Chlordiazepoxide | 10 |
| Clorazepate | 7.5 |
| Clotiazepam | 10 |
| Cloxazolam | 1.5 |
| Diazepam | 5 |
| Etizolam | 1.5 |
| Fludiazepam | 0.5 |
| Flutazolam^†^ | 15 |
| Flutoprazepam^†^ | 1.67 |
| Hydroxyzine | － |
| Loflazepate | 1.67 |
| Lorazepam | 1.2 |
| Medazepam | 10 |
| Mexazolam^†^ | 1.67 |
| Oxazepam | 15 |
| Oxazolam^†^ | 20 |
| Prazepam | 12.5 |
| Tandospirone^†^ | 25 |
| Tofisopam | 125 |

^†^Anxiolytics not indexed in the Anatomical Therapeutic Chemical classification 2021

**Antidepressants**

| Generic name | Dose equivalence (mg/day) |
| --- | --- |
| Amitriptyline | 150 |
| Amoxapine | 150 |
| Clomipramine | 120 |
| Desipramine | 150 |
| Dosulepin | 150 |
| Duloxetine | 30 |
| Escitalopram | 20 |
| Fluvoxamine | 150 |
| Imipramine | 150 |
| Lofepramine | 150 |
| Maprotiline | 150 |
| Mianserin | 60 |
| Milnacipran | 100 |
| Mirtazapine | 30 |
| Nortriptyline | 75 |
| Paroxetine | 40 |
| Safrazine^†^ | 30 |
| Sertraline | 100 |
| Setiptiline^†^ | 6 |
| Sulpiride | 300 |
| Trazodone | 300 |
| Trimipramine | 150 |
| Venlafaxine | 150 |

^†^Antidepressants not indexed in the Anatomical Therapeutic Chemical classification 2021

**Antipsychotics**

| Generic name | Dose equivalence (mg/day) |
| --- | --- |
| Aripiprazole | 4 |
| Asenapine | 2.5 |
| Blonanserin^†^ | 4 |
| Blonanserin^†^ (tape) | 20 |
| Brexpiprazole | 0.5 |
| Bromperidol | 2 |
| Carpipramine^†^ | 100 |
| Chlorpromazine | 100 |
| Clocapramine^†^ | 40 |
| Clozapine | 50 |
| Floropipamide^†^ | － |
| Fluphenazine | 2 |
| Haloperidol | 2 |
| Levomepromazine | 100 |
| Moperone | 12.5 |
| Mosapramine | 33 |
| Nemonapride^†^ | 4.5 |
| Olanzapine | 2.5 |
| Oxypertine | 80 |
| Paliperidone | 1.5 |
| Perospirone^†^ | 8 |
| Perphenazine | 10 |
| Pimozide | 4 |
| Prochlorperazine | 15 |
| Propericyazine | 20 |
| Quetiapine | 66 |
| Risperidone | 1 |
| Spiperone^†^ | 1 |
| Sulpiride | 200 |
| Sultopride | 200 |
| Tiapride | 100 |
| Thioridazine | 100 |
| Timiperone^†^ | 1.3 |
| Trifluoperazine | 5 |
| Zotepine | 66 |

^†^Antipsychotics not indexed in the Anatomical Therapeutic Chemical classification 2021
