# Supplementary material for: Changes in psychotropic polypharmacy and high‐potency prescription following policy change: Findings from a large scale Japanese claims database
Source: Psychiatry Clin Neurosci. 2022 Jul 2;76(9):475–7. doi: 10.1111/pcn.13432 (PMC9546399; doi:10.1111/pcn.13432)
Supplement: Supplementary file 9 — Table S4 Prescription of concomitant psychotropic drugs. [file PCN-76-475-s012.docx]

Table S4. Prescription of concomitant psychotropic drugs

|  | 2005 | 2006 | 2007 | 2008 | 2009 | 2010 | 2011 | 2012 | 2013 | 2014 | 2015 | 2016 | 2017 | 2018 | 2019 |  |
| --- | --- | --- | --- | --- | --- | --- | --- | --- | --- | --- | --- | --- | --- | --- | --- | --- |
| Anxiolytics | | | | | | | | | | | | | | | | |
| 1 | 85.2% | 86.5% | 86.9% | 87.3% | 87.3% | 86.4% | 86.8% | 87.1% | 86.9% | 87.0% | 87.1% | 87.4% | 87.8% | 88.1% | 88.4% |  |
| 2 | 13.1% | 11.5% | 11.7% | 11.1% | 11.1% | 12.1% | 11.7% | 11.4% | 11.7% | 11.7% | 12.1% | 11.8% | 11.6% | 11.3% | 11.1% |  |
| 3 ≤ | 1.7% | 2.0% | 1.4% | 1.5% | 1.6% | 1.5% | 1.5% | 1.5% | 1.4% | 1.3% | 0.8% | 0.7% | 0.6% | 0.5% | 0.6% |  |
| Hypnotics | | | | | | | | | | | | | | | | |
| 1 | 82.0% | 81.1% | 81.3% | 79.7% | 80.1% | 80.0% | 80.2% | 80.1% | 80.1% | 79.6% | 79.8% | 79.2% | 78.9% | 78.5% | 77.7% |  |
| 2 | 13.1% | 14.9% | 14.6% | 16.1% | 15.6% | 15.5% | 15.5% | 15.8% | 15.9% | 16.4% | 17.3% | 18.1% | 18.5% | 18.9% | 19.7% |  |
| 3 ≤ | 4.8% | 4.0% | 4.1% | 4.2% | 4.3% | 4.6% | 4.3% | 4.1% | 4.0% | 4.0% | 2.9% | 2.6% | 2.6% | 2.6% | 2.6% |  |
| Antidepressants | | | | | | | | | | | | | | | | |
| 1 | 69.3% | 69.5% | 69.7% | 70.0% | 70.7% | 71.8% | 72.0% | 72.8% | 74.0% | 74.4% | 75.1% | 76.2% | 77.5% | 78.2% | 78.6% |  |
| 2 | 21.4% | 22.0% | 22.6% | 22.9% | 23.0% | 21.9% | 21.9% | 21.5% | 20.8% | 20.7% | 20.6% | 20.6% | 19.9% | 19.5% | 19.1% |  |
| 3 ≤ | 8.9% | 8.5% | 7.8% | 7.1% | 6.3% | 6.4% | 6.1% | 5.7% | 5.1% | 4.9% | 4.3% | 3.1% | 2.6% | 2.3% | 2.3% |  |
| Antipsychotics | | | | | | | | | | | | | | | | |
| 1 | 70.7% | 67.7% | 70.6% | 74.8% | 72.7% | 73.6% | 74.9% | 74.0% | 74.7% | 76.2% | 76.7% | 77.6% | 78.1% | 78.4% | 78.8% |  |
| 2 | 20.1% | 23.1% | 20.5% | 19.2% | 20.6% | 19.5% | 17.8% | 18.8% | 18.4% | 18.0% | 18.0% | 18.2% | 18.8% | 18.9% | 18.3% |  |
| 3 ≤ | 9.2% | 9.1% | 8.9% | 6.0% | 6.7% | 6.8% | 7.3% | 7.3% | 7.0% | 5.9% | 5.3% | 4.2% | 3.1% | 2.7% | 2.9% |  |
| Anxiolytics and hypnotics | | | | | | | | | | | | | | | | |
| 1 | 69.4% | 70.0% | 69.2% | 70.0% | 69.2% | 69.0% | 69.2% | 69.1% | 69.0% | 69.2% | 68.7% | 68.2% | 68.2% | 67.9% | 67.4% |  |
| 2 | 20.3% | 19.7% | 21.8% | 20.4% | 21.1% | 21.2% | 20.9% | 21.3% | 21.6% | 21.3% | 22.3% | 22.9% | 23.0% | 23.2% | 23.6% |  |
| 3 | 6.9% | 7.3% | 5.8% | 6.5% | 6.6% | 6.8% | 6.9% | 6.7% | 6.5% | 6.8% | 6.7% | 6.8% | 6.8% | 7.1% | 7.4% |  |
| 4 ≤ | 3.4% | 3.0% | 3.2% | 3.1% | 3.1% | 3.1% | 3.0% | 2.9% | 2.9% | 2.8% | 2.3% | 2.1% | 2.0% | 1.7% | 1.6% |  |
