# Supplementary material for: Changes in psychotropic polypharmacy and high‐potency prescription following policy change: Findings from a large scale Japanese claims database
Source: Psychiatry Clin Neurosci. 2022 Jul 2;76(9):475–7. doi: 10.1111/pcn.13432 (PMC9546399; doi:10.1111/pcn.13432)
Supplement: Supplementary file 10 — Table S5 The proportion of those prescribed three or more psychotropic drugs among subscribers to the health insurance service who were prescribed psychotropic drugs (by 5‐year age group and sex). [file PCN-76-475-s001.docx]

Table S5. Proportion of those prescribed three or more psychotropic drugs among subscribers to the health insurance service who were prescribed psychotropic drugs (by 5-year age group and sex)

Anxiolytics (male)

|  | 2005 | 2006 | 2007 | 2008 | 2009 | 2010 | 2011 | 2012 | 2013 | 2014 | 2015 | 2016 | 2017 | 2018 | 2019 |
| --- | --- | --- | --- | --- | --- | --- | --- | --- | --- | --- | --- | --- | --- | --- | --- |
| 0–4 y | 0.00% | 0.00% | 0.00% | 0.00% | 0.00% | 0.00% | 0.00% | 0.00% | 0.00% | 0.00% | 0.00% | 0.00% | 0.00% | 0.00% | 0.00% |
| 5–9 y | 0.00% | 0.00% | 0.00% | 0.00% | 0.00% | 0.00% | 0.00% | 0.00% | 0.00% | 0.00% | 0.00% | 0.00% | 0.00% | 0.00% | 0.00% |
| 10–14 y | 0.00% | 0.00% | 0.00% | 0.00% | 0.00% | 0.00% | 0.00% | 0.00% | 0.00% | 0.00% | 0.00% | 0.00% | 0.58% | 0.00% | 0.00% |
| 15–19 y | 0.00% | 0.00% | 3.03% | 3.33% | 2.67% | 0.00% | 1.56% | 0.00% | 0.78% | 1.09% | 0.86% | 0.00% | 0.45% | 0.00% | 0.23% |
| 20–24 y | 1.37% | 4.60% | 0.00% | 2.92% | 2.50% | 1.42% | 1.64% | 1.68% | 0.82% | 0.96% | 0.37% | 0.24% | 0.21% | 0.61% | 0.40% |
| 25–29 y | 3.62% | 1.74% | 1.37% | 2.14% | 2.04% | 1.74% | 1.66% | 0.91% | 1.54% | 1.06% | 1.00% | 0.43% | 0.46% | 0.24% | 0.30% |
| 30–34 y | 1.91% | 2.22% | 1.26% | 3.32% | 2.44% | 1.93% | 2.39% | 1.41% | 1.54% | 1.83% | 0.95% | 0.96% | 0.80% | 0.62% | 0.22% |
| 35–39 y | 3.83% | 2.38% | 2.36% | 1.88% | 1.94% | 2.55% | 2.49% | 1.98% | 1.84% | 1.15% | 0.78% | 0.76% | 0.69% | 0.56% | 0.43% |
| 40–44 y | 2.01% | 1.28% | 2.55% | 1.84% | 1.91% | 2.57% | 2.00% | 1.99% | 2.20% | 1.57% | 0.75% | 0.55% | 0.55% | 0.62% | 0.69% |
| 45–49 y | 1.27% | 2.78% | 2.39% | 1.37% | 1.80% | 2.12% | 1.75% | 1.78% | 1.72% | 1.59% | 0.72% | 0.79% | 0.61% | 0.43% | 0.48% |
| 50–54 y | 1.46% | 1.06% | 1.73% | 1.96% | 1.90% | 1.64% | 1.43% | 1.63% | 1.88% | 1.89% | 0.87% | 0.77% | 0.61% | 0.51% | 0.45% |
| 55–59 y | 2.33% | 2.60% | 2.03% | 1.30% | 0.63% | 1.01% | 1.43% | 1.12% | 0.84% | 1.03% | 0.70% | 0.68% | 0.47% | 0.54% | 0.42% |
| 60–64 y | 0.00% | 0.00% | 0.00% | 0.63% | 0.00% | 0.00% | 0.94% | 0.34% | 0.58% | 0.48% | 0.27% | 0.42% | 0.38% | 0.39% | 0.23% |
| 65–69 y | 0.00% | 0.00% | 0.00% | 2.27% | 2.33% | 0.00% | 0.00% | 0.30% | 0.20% | 0.60% | 0.49% | 0.53% | 0.64% | 0.34% | 0.56% |
| 70–74 y | 0.00% | 0.00% | 0.00% | 0.00% | 0.00% | 1.23% | 0.86% | 0.81% | 0.63% | 0.88% | 0.44% | 0.46% | 0.22% | 0.00% | 0.22% |
| Total | 1.36% | 1.28% | 1.22% | 1.54% | 1.41% | 1.37% | 1.39% | 1.20% | 1.22% | 1.18% | 0.65% | 0.62% | 0.52% | 0.42% | 0.41% |

Table S5. Proportion of those prescribed three or more psychotropic drugs among subscribers to the health insurance service who were prescribed psychotropic drugs (by 5-year age group and sex)

Anxiolytics (female)

|  | 2005 | 2006 | 2007 | 2008 | 2009 | 2010 | 2011 | 2012 | 2013 | 2014 | 2015 | 2016 | 2017 | 2018 | 2019 |
| --- | --- | --- | --- | --- | --- | --- | --- | --- | --- | --- | --- | --- | --- | --- | --- |
| 0–4 y | 0.00% | 0.00% | 0.00% | 0.00% | 0.00% | 0.00% | 0.00% | 0.00% | 0.00% | 0.00% | 0.00% | 0.00% | 0.00% | 0.00% | 0.00% |
| 5–9 y | 0.00% | 0.00% | 0.00% | 0.00% | 0.00% | 0.00% | 0.00% | 0.00% | 0.00% | 0.00% | 0.00% | 0.00% | 0.00% | 0.00% | 0.00% |
| 10–14 y | 0.00% | 5.56% | 0.00% | 0.00% | 0.00% | 3.45% | 1.39% | 0.00% | 0.00% | 0.80% | 0.67% | 0.68% | 0.00% | 0.00% | 0.00% |
| 15–19 y | 0.00% | 1.59% | 7.14% | 5.00% | 0.00% | 2.89% | 1.17% | 2.11% | 2.44% | 1.77% | 0.59% | 0.86% | 1.14% | 1.20% | 0.44% |
| 20–24 y | 6.25% | 3.66% | 3.92% | 5.08% | 4.46% | 4.73% | 2.10% | 2.60% | 1.53% | 2.40% | 1.06% | 1.15% | 0.56% | 0.59% | 0.51% |
| 25–29 y | 2.61% | 0.88% | 4.84% | 4.30% | 4.86% | 3.80% | 1.38% | 2.65% | 1.59% | 1.97% | 1.83% | 1.44% | 1.35% | 0.63% | 0.75% |
| 30–34 y | 3.66% | 3.39% | 4.72% | 3.32% | 2.94% | 2.02% | 3.81% | 2.47% | 2.96% | 1.95% | 1.40% | 0.82% | 1.02% | 0.98% | 0.98% |
| 35–39 y | 3.33% | 3.53% | 3.01% | 3.89% | 3.00% | 2.80% | 3.34% | 2.70% | 2.74% | 2.56% | 1.61% | 1.45% | 1.18% | 0.75% | 1.13% |
| 40–44 y | 3.46% | 2.16% | 4.50% | 3.23% | 3.29% | 2.56% | 2.47% | 2.15% | 2.30% | 2.06% | 1.39% | 0.99% | 0.77% | 0.79% | 0.81% |
| 45–49 y | 2.21% | 1.98% | 2.51% | 3.28% | 2.20% | 2.15% | 2.54% | 2.56% | 2.07% | 1.77% | 1.23% | 1.09% | 0.76% | 0.84% | 0.77% |
| 50–54 y | 2.02% | 1.85% | 0.48% | 0.59% | 1.88% | 1.74% | 2.19% | 2.11% | 2.11% | 2.04% | 0.95% | 0.86% | 1.06% | 0.89% | 0.88% |
| 55–59 y | 0.87% | 1.82% | 1.14% | 2.16% | 1.62% | 1.00% | 1.12% | 1.57% | 1.75% | 1.26% | 1.08% | 0.73% | 0.83% | 0.59% | 0.60% |
| 60–64 y | 2.33% | 2.20% | 1.16% | 0.00% | 1.03% | 1.21% | 0.92% | 0.68% | 1.09% | 1.12% | 0.40% | 0.40% | 0.46% | 0.51% | 0.58% |
| 65–69 y | 0.95% | 3.81% | 0.00% | 0.71% | 1.22% | 1.44% | 0.73% | 1.54% | 0.47% | 0.81% | 1.03% | 0.56% | 0.38% | 0.55% | 0.67% |
| 70–74 y | 1.56% | 1.75% | 0.68% | 0.50% | 0.70% | 0.55% | 0.95% | 0.86% | 0.95% | 0.75% | 0.59% | 0.76% | 0.41% | 0.17% | 0.28% |
| Total | 1.94% | 2.43% | 1.53% | 1.55% | 1.72% | 1.59% | 1.54% | 1.60% | 1.46% | 1.37% | 0.97% | 0.81% | 0.68% | 0.59% | 0.65% |

Table S5. Proportion of those prescribed three or more psychotropic drugs among subscribers to the health insurance service who were prescribed psychotropic drugs (by 5-year age group and sex)

Hypnotics (male)

|  | 2005 | 2006 | 2007 | 2008 | 2009 | 2010 | 2011 | 2012 | 2013 | 2014 | 2015 | 2016 | 2017 | 2018 | 2019 |
| --- | --- | --- | --- | --- | --- | --- | --- | --- | --- | --- | --- | --- | --- | --- | --- |
| 0–4 y | 0.00% | 0.00% | 0.00% | 0.00% | 0.00% | 0.00% | 0.00% | 0.00% | 0.00% | 4.55% | 0.00% | 0.00% | 0.00% | 0.00% | 0.00% |
| 5–9 y | 0.00% | 0.00% | 0.00% | 0.00% | 0.00% | 0.00% | 0.00% | 0.00% | 0.00% | 0.00% | 0.00% | 0.00% | 0.00% | 0.99% | 0.00% |
| 10–14 y | 0.00% | 0.00% | 0.00% | 0.00% | 0.00% | 0.00% | 0.00% | 0.00% | 0.00% | 1.83% | 0.68% | 1.07% | 0.82% | 0.00% | 1.05% |
| 15–19 y | 0.00% | 0.00% | 0.00% | 11.4% | 4.00% | 5.75% | 3.92% | 2.72% | 2.65% | 3.81% | 2.68% | 2.39% | 2.67% | 1.46% | 0.80% |
| 20–24 y | 6.67% | 9.26% | 10.1% | 5.26% | 7.26% | 6.85% | 4.53% | 4.65% | 5.14% | 4.14% | 3.95% | 3.49% | 2.54% | 2.37% | 2.10% |
| 25–29 y | 2.83% | 11.1% | 8.61% | 7.89% | 6.09% | 8.51% | 5.74% | 5.08% | 6.56% | 5.29% | 4.21% | 2.94% | 3.59% | 2.95% | 3.35% |
| 30–34 y | 6.57% | 5.98% | 6.84% | 10.6% | 7.65% | 8.12% | 8.83% | 6.91% | 6.48% | 6.53% | 3.85% | 3.99% | 3.60% | 3.06% | 2.92% |
| 35–39 y | 7.00% | 11.3% | 8.65% | 8.80% | 8.01% | 8.42% | 7.78% | 7.01% | 6.73% | 5.86% | 4.43% | 4.49% | 4.21% | 3.70% | 3.47% |
| 40–44 y | 9.34% | 7.74% | 9.97% | 8.17% | 8.22% | 8.51% | 8.25% | 7.09% | 6.59% | 6.10% | 3.94% | 3.90% | 3.87% | 3.43% | 3.64% |
| 45–49 y | 6.60% | 6.25% | 6.07% | 7.46% | 7.86% | 6.28% | 7.73% | 6.31% | 6.43% | 5.80% | 3.89% | 3.74% | 3.75% | 3.61% | 3.20% |
| 50–54 y | 5.95% | 9.47% | 6.90% | 6.57% | 5.47% | 4.33% | 6.18% | 5.60% | 5.71% | 5.14% | 4.10% | 3.60% | 3.52% | 3.48% | 3.14% |
| 55–59 y | 7.04% | 5.18% | 6.58% | 3.22% | 5.25% | 6.03% | 4.49% | 4.70% | 4.46% | 4.55% | 3.30% | 3.12% | 2.88% | 2.96% | 3.17% |
| 60–64 y | 2.90% | 3.75% | 2.94% | 5.38% | 3.59% | 3.50% | 3.26% | 2.85% | 2.47% | 2.54% | 1.91% | 2.12% | 2.18% | 2.05% | 2.26% |
| 65–69 y | 3.85% | 0.00% | 0.00% | 0.00% | 2.67% | 2.27% | 2.70% | 2.83% | 2.68% | 2.74% | 2.01% | 1.45% | 1.50% | 2.04% | 1.29% |
| 70–74 y | 0.00% | 0.00% | 0.00% | 2.08% | 2.38% | 4.76% | 2.53% | 3.44% | 2.93% | 3.64% | 2.15% | 2.01% | 1.58% | 1.94% | 1.91% |
| Total | 5.01% | 4.45% | 4.19% | 4.70% | 4.78% | 5.26% | 4.76% | 4.56% | 4.37% | 4.33% | 3.03% | 2.81% | 2.69% | 2.70% | 2.58% |

Table S5. Proportion of those prescribed three or more psychotropic drugs among subscribers to the health insurance service who were prescribed psychotropic drugs (by 5-year age group and sex)

Hypnotics (female)

|  | 2005 | 2006 | 2007 | 2008 | 2009 | 2010 | 2011 | 2012 | 2013 | 2014 | 2015 | 2016 | 2017 | 2018 | 2019 |
| --- | --- | --- | --- | --- | --- | --- | --- | --- | --- | --- | --- | --- | --- | --- | --- |
| 0–4 y | 0.00% | 0.00% | 0.00% | 0.00% | 0.00% | 0.00% | 0.00% | 0.00% | 0.00% | 0.00% | 0.00% | 0.00% | 0.00% | 0.00% | 0.00% |
| 5–9 y | 0.00% | 0.00% | 0.00% | 0.00% | 0.00% | 0.00% | 0.00% | 0.00% | 0.00% | 0.00% | 2.13% | 2.08% | 0.00% | 0.00% | 1.28% |
| 10–14 y | 12.5% | 0.00% | 0.00% | 0.00% | 14.3% | 3.85% | 0.00% | 2.33% | 2.70% | 1.02% | 1.50% | 0.69% | 0.53% | 0.91% | 1.70% |
| 15–19 y | 0.00% | 2.94% | 4.88% | 5.88% | 3.13% | 8.55% | 4.80% | 5.08% | 5.32% | 3.40% | 2.28% | 2.29% | 2.71% | 1.93% | 1.73% |
| 20–24 y | 5.56% | 7.84% | 5.26% | 2.44% | 5.06% | 9.70% | 10.7% | 10.8% | 10.1% | 7.49% | 5.09% | 3.81% | 4.49% | 2.63% | 2.91% |
| 25–29 y | 5.33% | 10.7% | 5.68% | 11.4% | 8.29% | 9.95% | 7.11% | 8.05% | 7.28% | 8.53% | 5.55% | 5.21% | 3.51% | 4.54% | 3.02% |
| 30–34 y | 9.92% | 12.6% | 6.75% | 5.21% | 8.47% | 7.66% | 7.44% | 7.38% | 8.68% | 7.59% | 5.57% | 4.72% | 3.88% | 3.47% | 3.84% |
| 35–39 y | 7.28% | 8.28% | 5.49% | 11.3% | 7.97% | 7.85% | 8.46% | 8.89% | 8.65% | 6.96% | 4.37% | 4.25% | 4.74% | 4.08% | 3.52% |
| 40–44 y | 3.45% | 7.24% | 8.74% | 9.34% | 6.97% | 8.14% | 7.04% | 7.68% | 7.30% | 6.94% | 5.12% | 4.77% | 4.46% | 4.40% | 3.47% |
| 45–49 y | 8.26% | 6.61% | 5.26% | 5.24% | 6.29% | 8.30% | 7.44% | 6.22% | 6.98% | 6.54% | 4.24% | 4.41% | 3.94% | 3.47% | 3.79% |
| 50–54 y | 9.70% | 8.47% | 6.25% | 5.94% | 5.22% | 5.86% | 5.90% | 5.68% | 5.59% | 5.20% | 3.69% | 3.29% | 3.64% | 3.58% | 3.16% |
| 55–59 y | 4.19% | 3.72% | 5.81% | 4.37% | 4.59% | 4.18% | 3.42% | 3.01% | 3.71% | 3.68% | 2.91% | 2.96% | 2.91% | 2.90% | 3.44% |
| 60–64 y | 3.66% | 1.30% | 3.49% | 2.91% | 4.43% | 3.02% | 3.07% | 3.33% | 2.97% | 2.92% | 1.99% | 1.78% | 1.68% | 1.95% | 1.85% |
| 65–69 y | 5.43% | 2.02% | 0.91% | 3.11% | 2.17% | 1.75% | 2.22% | 2.38% | 2.04% | 2.75% | 2.14% | 1.91% | 1.79% | 1.82% | 2.17% |
| 70–74 y | 0.00% | 1.61% | 4.08% | 1.41% | 2.34% | 2.64% | 2.51% | 1.90% | 1.73% | 1.87% | 1.58% | 1.10% | 1.42% | 1.47% | 1.95% |
| Total | 4.70% | 3.67% | 4.02% | 3.83% | 4.06% | 4.10% | 3.96% | 3.82% | 3.74% | 3.75% | 2.75% | 2.47% | 2.48% | 2.46% | 2.62% |

Table S5. Proportion of those prescribed three or more psychotropic drugs among subscribers to the health insurance service who were prescribed psychotropic drugs (by 5-year age group and sex)

Antidepressants (male)

|  | 2005 | 2006 | 2007 | 2008 | 2009 | 2010 | 2011 | 2012 | 2013 | 2014 | 2015 | 2016 | 2017 | 2018 | 2019 |
| --- | --- | --- | --- | --- | --- | --- | --- | --- | --- | --- | --- | --- | --- | --- | --- |
| 0–4 y | 0.00% | 0.00% | 0.00% | 0.00% | 0.00% | 0.00% | 0.00% | 0.00% | 0.00% | 0.00% | 0.00% | 0.00% | 0.00% | 0.00% | 0.00% |
| 5–9 y | 0.00% | 0.00% | 0.00% | 0.00% | 0.00% | 0.00% | 0.00% | 0.00% | 0.00% | 0.00% | 0.00% | 0.00% | 0.00% | 0.86% | 0.00% |
| 10–14 y | 3.70% | 0.00% | 0.00% | 3.33% | 1.72% | 0.00% | 0.00% | 0.61% | 0.50% | 0.00% | 0.00% | 0.79% | 0.36% | 0.38% | 0.71% |
| 15–19 y | 25.0% | 3.70% | 3.13% | 0.00% | 1.49% | 2.40% | 3.29% | 0.94% | 1.30% | 2.68% | 1.49% | 1.96% | 1.54% | 0.35% | 0.47% |
| 20–24 y | 25.9% | 7.89% | 9.78% | 5.83% | 4.52% | 7.27% | 5.65% | 6.57% | 4.67% | 3.86% | 3.40% | 2.94% | 2.09% | 1.74% | 1.72% |
| 25–29 y | 33.1% | 12.2% | 14.1% | 11.9% | 10.2% | 7.82% | 5.82% | 6.55% | 5.56% | 4.80% | 4.46% | 2.70% | 3.05% | 2.41% | 3.03% |
| 30–34 y | 22.14 | 16.3% | 13.9% | 12.0% | 8.50% | 8.38% | 9.37% | 6.65% | 6.99% | 5.76% | 4.65% | 2.97% | 2.55% | 2.81% | 2.34% |
| 35–39 y | 13.3% | 16.4% | 15.1% | 14.0% | 11.3% | 9.74% | 9.28% | 9.26% | 7.21% | 6.79% | 6.18% | 4.52% | 3.93% | 3.38% | 2.79% |
| 40–44 y | 9.51% | 15.9% | 15.3% | 15.2% | 10.4% | 10.7% | 9.76% | 10.2% | 9.04% | 7.51% | 6.69% | 4.49% | 3.48% | 3.23% | 3.26% |
| 45–49 y | 8.33% | 16.3% | 12.2% | 9.09% | 10.8% | 10.7% | 9.53% | 8.77% | 7.65% | 7.32% | 6.26% | 5.03% | 4.24% | 3.63% | 3.32% |
| 50–54 y | 7.03% | 10.0% | 13.7% | 12.8% | 11.2% | 9.87% | 9.60% | 6.67% | 6.89% | 7.05% | 6.55% | 4.79% | 3.95% | 4.06% | 3.59% |
| 55–59 y | 3.81% | 7.46% | 4.79% | 7.69% | 7.72% | 10.5% | 10.8% | 8.38% | 7.83% | 5.87% | 6.76% | 4.45% | 3.62% | 3.56% | 3.09% |
| 60–64 y | 0.00% | 12.8% | 6.06% | 4.41% | 4.76% | 4.46% | 5.15% | 6.28% | 4.59% | 5.34% | 3.37% | 3.38% | 2.32% | 2.66% | 2.57% |
| 65–69 y | 0.00% | 0.00% | 0.00% | 7.69% | 5.71% | 2.50% | 3.77% | 3.85% | 3.16% | 2.36% | 2.84% | 1.63% | 1.86% | 2.23% | 1.25% |
| 70–74 y | 0.00% | 0.00% | 0.00% | 0.00% | 0.00% | 3.03% | 7.69% | 3.33% | 4.72% | 4.61% | 1.57% | 1.03% | 0.94% | 0.41% | 1.88% |
| Total | 11.0% | 11.9% | 10.6% | 10.0% | 8.26% | 8.26% | 8.37% | 7.41% | 6.70% | 6.06% | 5.34% | 3.85% | 3.23% | 3.02% | 2.83% |

Table S5. Proportion of those prescribed three or more psychotropic drugs among subscribers to the health insurance service who were prescribed psychotropic drugs (by 5-year age group and sex)

Antidepressants (female)

|  | 2005 | 2006 | 2007 | 2008 | 2009 | 2010 | 2011 | 2012 | 2013 | 2014 | 2015 | 2016 | 2017 | 2018 | 2019 |
| --- | --- | --- | --- | --- | --- | --- | --- | --- | --- | --- | --- | --- | --- | --- | --- |
| 0–4 y | 0.00% | 0.00% | 0.00% | 0.00% | 0.00% | 0.00% | 0.00% | 0.00% | 0.00% | 0.00% | 0.00% | 0.00% | 0.00% | 0.00% | 0.00% |
| 5–9 y | 0.00% | 0.00% | 0.00% | 0.00% | 0.00% | 0.00% | 0.00% | 0.00% | 0.00% | 0.00% | 0.00% | 0.00% | 0.00% | 0.00% | 0.00% |
| 10–14 y | 0.00% | 0.00% | 0.00% | 0.00% | 0.00% | 4.76% | 0.00% | 0.00% | 0.00% | 0.81% | 0.00% | 0.00% | 0.00% | 0.80% | 0.85% |
| 15–19 y | 2.33% | 4.76% | 6.35% | 11.1% | 2.30% | 3.33% | 2.65% | 2.52% | 2.73% | 1.64% | 1.30% | 1.21% | 1.76% | 0.81% | 0.23% |
| 20–24 y | 13.0% | 12.3% | 8.51% | 5.04% | 4.37% | 3.80% | 3.90% | 5.87% | 2.97% | 2.75% | 3.28% | 1.70% | 1.46% | 0.94% | 1.04% |
| 25–29 y | 8.43% | 4.59% | 6.40% | 6.13% | 6.49% | 4.64% | 4.11% | 3.92% | 3.07% | 4.12% | 2.40% | 2.39% | 1.32% | 0.82% | 1.34% |
| 30–34 y | 6.90% | 4.17% | 4.32% | 4.80% | 6.21% | 4.72% | 4.89% | 3.77% | 3.43% | 3.49% | 2.56% | 1.65% | 2.10% | 1.68% | 1.54% |
| 35–39 y | 7.26% | 8.56% | 7.17% | 5.29% | 4.99% | 4.70% | 3.54% | 3.33% | 3.59% | 3.31% | 3.81% | 2.36% | 1.38% | 1.46% | 1.25% |
| 40–44 y | 3.68% | 4.19% | 6.83% | 7.79% | 6.95% | 5.47% | 6.36% | 5.67% | 4.18% | 4.49% | 4.11% | 2.41% | 1.67% | 1.48% | 1.62% |
| 45–49 y | 6.32% | 5.77% | 6.20% | 7.18% | 5.18% | 6.12% | 5.38% | 5.70% | 5.74% | 5.51% | 4.19% | 2.88% | 1.91% | 1.52% | 1.65% |
| 50–54 y | 4.88% | 3.96% | 7.29% | 5.33% | 4.20% | 5.81% | 4.76% | 5.20% | 4.74% | 3.98% | 4.55% | 3.21% | 2.55% | 2.33% | 2.28% |
| 55–59 y | 10.9% | 9.09% | 6.52% | 5.88% | 5.20% | 4.57% | 3.97% | 5.50% | 4.07% | 3.68% | 3.26% | 2.56% | 2.33% | 2.06% | 2.10% |
| 60–64 y | 8.82% | 11.1% | 7.32% | 1.39% | 3.52% | 6.02% | 4.47% | 5.40% | 4.58% | 4.23% | 2.97% | 2.44% | 1.51% | 2.58% | 2.32% |
| 65–69 y | 8.57% | 3.03% | 2.38% | 3.03% | 5.21% | 6.67% | 4.15% | 4.23% | 3.63% | 3.67% | 3.77% | 3.61% | 2.21% | 1.65% | 1.96% |
| 70–74 y | 0.00% | 0.00% | 2.33% | 2.99% | 2.13% | 1.59% | 2.38% | 1.50% | 2.38% | 3.27% | 2.77% | 1.86% | 2.48% | 1.45% | 2.35% |
| Total | 7.05% | 5.57% | 5.37% | 4.70% | 4.69% | 4.87% | 4.21% | 4.32% | 3.81% | 3.86% | 3.44% | 2.53% | 1.99% | 1.68% | 1.86% |

Table S5. Proportion of those prescribed three or more psychotropic drugs among subscribers to the health insurance service who were prescribed psychotropic drugs (by 5-year age group and sex)

Antipsychotics (male)

|  | 2005 | 2006 | 2007 | 2008 | 2009 | 2010 | 2011 | 2012 | 2013 | 2014 | 2015 | 2016 | 2017 | 2018 | 2019 |
| --- | --- | --- | --- | --- | --- | --- | --- | --- | --- | --- | --- | --- | --- | --- | --- |
| 0–4 y | 0.00% | 0.00% | 0.00% | 0.00% | 0.00% | 0.00% | 0.00% | 0.00% | 0.00% | 0.00% | 0.00% | 0.00% | 0.00% | 0.00% | 0.00% |
| 5–9 y | 14.3% | 0.00% | 0.00% | 0.00% | 0.00% | 0.00% | 1.41% | 0.89% | 0.55% | 1.57% | 2.49% | 1.39% | 0.22% | 0.18% | 0.61% |
| 10–14 y | 17.7% | 6.67% | 5.56% | 3.23% | 7.32% | 4.08% | 2.22% | 1.03% | 2.34% | 2.28% | 2.63% | 2.38% | 2.34% | 1.04% | 1.05% |
| 15–19 y | 4.35% | 11.5% | 0.00% | 4.00% | 2.74% | 5.11% | 5.70% | 7.06% | 6.33% | 7.61% | 4.92% | 3.58% | 4.21% | 2.84% | 2.52% |
| 20–24 y | 7.14% | 14.3% | 11.9% | 8.45% | 7.34% | 11.1% | 7.39% | 7.19% | 6.59% | 7.52% | 5.31% | 5.18% | 4.71% | 3.44% | 3.92% |
| 25–29 y | 10.9% | 8.47% | 9.09% | 11.4% | 14.4% | 12.2% | 10.5% | 10.3% | 6.66% | 6.42% | 7.72% | 4.98% | 4.23% | 4.47% | 4.01% |
| 30–34 y | 12.1% | 5.26% | 5.68% | 6.86% | 9.22% | 8.10% | 9.32% | 8.87% | 8.49% | 7.44% | 6.92% | 3.97% | 3.29% | 2.13% | 2.81% |
| 35–39 y | 5.97% | 6.35% | 5.95% | 7.62% | 4.03% | 5.00% | 6.83% | 6.32% | 7.30% | 5.80% | 5.68% | 4.50% | 2.53% | 2.66% | 2.57% |
| 40–44 y | 4.92% | 9.68% | 8.54% | 9.90% | 5.81% | 6.69% | 6.60% | 6.91% | 5.79% | 4.39% | 4.26% | 3.11% | 2.48% | 2.24% | 1.72% |
| 45–49 y | 12.8% | 11.4% | 10.0% | 10.9% | 10.0% | 4.33% | 6.17% | 5.94% | 5.56% | 5.06% | 3.46% | 2.02% | 1.82% | 1.52% | 1.77% |
| 50–54 y | 6.45% | 11.4% | 3.45% | 2.13% | 6.76% | 7.95% | 6.76% | 4.19% | 4.78% | 3.77% | 3.58% | 2.88% | 1.70% | 1.73% | 2.06% |
| 55–59 y | 6.06% | 9.09% | 9.76% | 0.00% | 11.8% | 4.24% | 7.03% | 5.00% | 6.20% | 3.53% | 3.39% | 3.13% | 2.22% | 2.14% | 1.98% |
| 60–64 y | 0.00% | 0.00% | 11.1% | 3.03% | 0.00% | 4.17% | 6.52% | 4.72% | 7.65% | 7.77% | 4.44% | 3.04% | 1.81% | 1.41% | 1.93% |
| 65–69 y | 0.00% | 0.00% | 0.00% | 0.00% | 7.14% | 7.14% | 16.0% | 7.58% | 5.75% | 2.13% | 1.91% | 1.88% | 1.92% | 1.10% | 1.81% |
| 70–74 y | 0.00% | 0.00% | 0.00% | 0.00% | 0.00% | 0.00% | 0.00% | 4.84% | 5.41% | 4.40% | 2.44% | 3.85% | 1.90% | 1.83% | 4.39% |
| Total | 5.98% | 5.39% | 7.20% | 4.52% | 6.10% | 5.64% | 6.76% | 6.20% | 6.00% | 5.04% | 4.31% | 3.36% | 2.52% | 2.09% | 2.37% |

Table S5. Proportion of those prescribed three or more psychotropic drugs among subscribers to the health insurance service who were prescribed psychotropic drugs (by 5-year age group and sex)

Antipsychotics (female)

|  | 2005 | 2006 | 2007 | 2008 | 2009 | 2010 | 2011 | 2012 | 2013 | 2014 | 2015 | 2016 | 2017 | 2018 | 2019 |
| --- | --- | --- | --- | --- | --- | --- | --- | --- | --- | --- | --- | --- | --- | --- | --- |
| 0–4 y | 0.00% | 0.00% | 0.00% | 0.00% | 0.00% | 0.00% | 0.00% | 0.00% | 0.00% | 0.00% | 0.00% | 0.00% | 0.00% | 0.00% | 0.00% |
| 5–9 y | 0.00% | 0.00% | 0.00% | 0.00% | 0.00% | 0.00% | 0.00% | 0.00% | 0.00% | 0.00% | 0.00% | 0.00% | 0.00% | 0.00% | 0.00% |
| 10–14 y | 9.09% | 0.00% | 0.00% | 0.00% | 3.45% | 5.45% | 0.00% | 2.22% | 6.35% | 2.17% | 3.41% | 2.48% | 0.87% | 2.27% | 1.13% |
| 15–19 y | 12.0% | 10.5% | 18.8% | 6.98% | 1.67% | 8.74% | 4.08% | 6.07% | 4.56% | 4.96% | 5.18% | 3.08% | 3.76% | 1.97% | 2.61% |
| 20–24 y | 5.88% | 8.00% | 13.6% | 12.9% | 7.81% | 8.21% | 9.47% | 6.79% | 9.11% | 6.98% | 7.40% | 5.50% | 2.82% | 3.09% | 4.01% |
| 25–29 y | 8.00% | 9.80% | 12.0% | 8.33% | 7.08% | 8.57% | 9.13% | 8.68% | 9.38% | 7.61% | 6.89% | 5.30% | 4.36% | 3.60% | 4.25% |
| 30–34 y | 8.96% | 9.46% | 10.5% | 8.47% | 13.0% | 10.3% | 8.28% | 10.4% | 9.46% | 6.93% | 7.20% | 4.91% | 4.19% | 4.36% | 4.02% |
| 35–39 y | 17.2% | 18.1% | 10.8% | 9.84% | 9.39% | 6.69% | 10.3% | 11.0% | 9.70% | 9.30% | 7.04% | 4.58% | 3.99% | 3.07% | 3.50% |
| 40–44 y | 16.7% | 12.7% | 9.57% | 14.0% | 11.6% | 9.17% | 6.97% | 8.40% | 7.85% | 6.96% | 6.77% | 5.33% | 3.85% | 3.17% | 3.40% |
| 45–49 y | 14.3% | 6.90% | 6.06% | 10.3% | 10.7% | 10.5% | 9.07% | 9.98% | 7.70% | 6.22% | 6.08% | 4.17% | 2.93% | 3.66% | 3.18% |
| 50–54 y | 13.5% | 10.0% | 10.0% | 4.76% | 5.88% | 6.13% | 6.98% | 9.17% | 9.61% | 8.87% | 7.46% | 4.81% | 3.26% | 3.26% | 2.91% |
| 55–59 y | 12.5% | 8.11% | 8.57% | 7.02% | 9.26% | 7.39% | 7.32% | 9.41% | 8.22% | 6.94% | 6.17% | 6.21% | 4.18% | 3.62% | 3.65% |
| 60–64 y | 14.3% | 27.3% | 6.67% | 3.33% | 4.08% | 4.55% | 6.61% | 8.00% | 8.67% | 6.54% | 5.55% | 4.97% | 3.49% | 2.80% | 2.98% |
| 65–69 y | 7.14% | 16.7% | 14.3% | 4.55% | 4.76% | 11.4% | 9.23% | 9.62% | 5.81% | 5.06% | 6.06% | 4.64% | 4.24% | 2.95% | 2.92% |
| 70–74 y | 0.00% | 0.00% | 6.25% | 0.00% | 0.00% | 2.17% | 4.92% | 1.05% | 2.36% | 2.13% | 2.13% | 4.12% | 2.15% | 2.76% | 2.12% |
| Total | 11.2% | 11.6% | 9.74% | 6.95% | 7.08% | 7.52% | 7.64% | 8.02% | 7.59% | 6.39% | 6.01% | 4.80% | 3.52% | 3.23% | 3.21% |
