# Supplementary material for: Changes in psychotropic polypharmacy and high‐potency prescription following policy change: Findings from a large scale Japanese claims database
Source: Psychiatry Clin Neurosci. 2022 Jul 2;76(9):475–7. doi: 10.1111/pcn.13432 (PMC9546399; doi:10.1111/pcn.13432)
Supplement: Supplementary file 11 — Table S6 Potency of psychotropics. [file PCN-76-475-s004.docx]

Table S6. Potency of psychotropics

|  | 2005 | 2006 | 2007 | 2008 | 2009 | 2010 | 2011 | 2012 | 2013 | 2014 | 2015 | 2016 | 2017 | 2018 | 2019 |
| --- | --- | --- | --- | --- | --- | --- | --- | --- | --- | --- | --- | --- | --- | --- | --- |
| Anxiolytics | 6.1 | 5.9 | 5.6 | 5.8 | 5.8 | 5.8 | 5.8 | 5.7 | 5.8 | 5.7 | 5.8 | 5.9 | 5.7 | 5.7 | 5.9 |
| Hypnotics | 1.2 | 1.1 | 1.1 | 1.2 | 1.3 | 1.3 | 1.3 | 1.2 | 1.3 | 1.3 | 1.2 | 1.3 | 1.2 | 1.2 | 1.3 |
| Antidepressants | 95 | 91 | 93 | 94 | 93 | 93 | 103 | 104 | 108 | 111 | 110 | 113 | 113 | 115 | 122 |
| Antipsychotics | 332 | 340 | 354 | 290 | 287 | 289 | 292 | 286 | 300 | 283 | 271 | 271 | 259 | 245 | 257 |

Note: Values are presented as average potency per day (mg/day). Anxiolytic, hypnotic, antidepressant, and antipsychotic doses were converted to diazepam, flunitrazepam, imipramine, and chlorpromazine equivalent doses, respectively. As the values were adjusted for 5-year age group and sex using the census data, standard deviations could not be calculated.
