# Supplementary material for: Changes in psychotropic polypharmacy and high‐potency prescription following policy change: Findings from a large scale Japanese claims database
Source: Psychiatry Clin Neurosci. 2022 Jul 2;76(9):475–7. doi: 10.1111/pcn.13432 (PMC9546399; doi:10.1111/pcn.13432)
Supplement: Supplementary file 12 — Table S7 The proportion of those prescribed high‐potency psychotropics among subscribers to the health insurance service who were prescribed psychotropic drugs (by 5‐year age group and sex). [file PCN-76-475-s007.docx]

Table S7. Proportion of those prescribed high-potency psychotropics among subscribers to the health insurance service who were prescribed psychotropic drugs (by 5-year age group and sex)

Anxiolytics (male)

|  | 2005 | 2006 | 2007 | 2008 | 2009 | 2010 | 2011 | 2012 | 2013 | 2014 | 2015 | 2016 | 2017 | 2018 | 2019 |
| --- | --- | --- | --- | --- | --- | --- | --- | --- | --- | --- | --- | --- | --- | --- | --- |
| 0–4 y | 0.00% | 20.0% | 0.00% | 0.00% | 20.0% | 0.00% | 0.00% | 0.00% | 0.00% | 0.00% | 0.00% | 0.00% | 3.03% | 0.00% | 0.00% |
| 5–9 y | 0.00% | 0.00% | 9.09% | 9.09% | 0.00% | 4.00% | 4.35% | 0.00% | 5.41% | 0.00% | 4.65% | 3.39% | 2.08% | 1.64% | 0.00% |
| 10–14 y | 0.00% | 0.00% | 0.00% | 7.69% | 4.35% | 1.92% | 1.79% | 2.78% | 2.88% | 2.94% | 3.15% | 2.56% | 2.92% | 0.79% | 1.76% |
| 15–19 y | 2.78% | 0.00% | 0.00% | 3.33% | 0.00% | 2.94% | 0.78% | 1.74% | 1.17% | 1.82% | 2.01% | 2.36% | 2.49% | 3.91% | 3.66% |
| 20–24 y | 2.74% | 9.20% | 9.38% | 10.2% | 10.6% | 5.69% | 6.58% | 5.05% | 4.08% | 5.92% | 5.10% | 5.04% | 5.11% | 4.15% | 4.11% |
| 25–29 y | 10.1% | 5.23% | 6.85% | 7.26% | 8.50% | 8.12% | 7.62% | 6.21% | 5.98% | 7.00% | 5.61% | 5.58% | 5.82% | 5.57% | 4.89% |
| 30–34 y | 11.8% | 8.57% | 9.78% | 8.03% | 8.43% | 8.95% | 8.24% | 6.34% | 7.57% | 7.01% | 8.04% | 7.62% | 7.50% | 6.96% | 6.18% |
| 35–39 y | 8.16% | 8.55% | 9.22% | 10.2% | 7.93% | 7.95% | 9.81% | 8.51% | 8.73% | 7.67% | 9.39% | 8.64% | 7.46% | 6.83% | 8.21% |
| 40–44 y | 9.46% | 7.67% | 6.03% | 8.68% | 8.14% | 8.40% | 8.39% | 7.41% | 8.42% | 8.00% | 8.93% | 9.13% | 8.07% | 7.65% | 8.04% |
| 45–49 y | 12.3% | 10.1% | 8.19% | 7.09% | 9.00% | 7.89% | 8.06% | 7.87% | 7.95% | 7.88% | 7.14% | 8.25% | 7.52% | 7.22% | 7.68% |
| 50–54 y | 9.22% | 6.88% | 9.96% | 8.17% | 10.3% | 8.53% | 8.47% | 7.25% | 7.95% | 7.86% | 6.84% | 7.82% | 6.98% | 6.87% | 7.30% |
| 55–59 y | 7.44% | 6.06% | 5.28% | 7.79% | 5.00% | 5.34% | 5.34% | 6.78% | 6.84% | 6.45% | 6.76% | 6.81% | 6.26% | 5.88% | 6.35% |
| 60–64 y | 3.57% | 5.49% | 2.25% | 6.29% | 4.25% | 3.30% | 5.03% | 4.22% | 4.97% | 4.59% | 5.29% | 5.75% | 5.56% | 5.58% | 5.43% |
| 65–69 y | 0.00% | 3.85% | 0.00% | 6.82% | 6.98% | 2.94% | 1.34% | 2.10% | 2.57% | 2.18% | 4.37% | 3.52% | 3.83% | 3.82% | 5.31% |
| 70–74 y | 0.00% | 0.00% | 0.00% | 8.33% | 1.96% | 4.94% | 3.45% | 4.47% | 3.14% | 4.40% | 3.54% | 2.78% | 2.22% | 2.08% | 2.42% |
| Total | 6.15% | 5.98% | 5.02% | 7.89% | 6.65% | 6.20% | 6.13% | 5.89% | 6.14% | 6.08% | 6.37% | 6.42% | 5.96% | 5.71% | 6.13% |

Table S7. Proportion of those prescribed high-potency psychotropics among subscribers to the health insurance service who were prescribed psychotropic drugs (by 5-year age group and sex)

Anxiolytics (female)

|  | 2005 | 2006 | 2007 | 2008 | 2009 | 2010 | 2011 | 2012 | 2013 | 2014 | 2015 | 2016 | 2017 | 2018 | 2019 |
| --- | --- | --- | --- | --- | --- | --- | --- | --- | --- | --- | --- | --- | --- | --- | --- |
| 0–4 y | 0.00% | 0.00% | 0.00% | 0.00% | 0.00% | 0.00% | 0.00% | 0.00% | 0.00% | 0.00% | 3.33% | 0.00% | 0.00% | 0.00% | 0.00% |
| 5–9 y | 0.00% | 0.00% | 0.00% | 0.00% | 0.00% | 0.00% | 0.00% | 0.00% | 2.86% | 2.63% | 2.63% | 1.96% | 2.13% | 2.13% | 0.00% |
| 10–14 y | 5.56% | 0.00% | 0.00% | 0.00% | 7.14% | 1.72% | 1.39% | 0.00% | 0.00% | 0.80% | 1.33% | 1.37% | 2.17% | 0.56% | 0.00% |
| 15–19 y | 3.64% | 1.59% | 4.76% | 1.25% | 4.71% | 4.62% | 4.09% | 4.23% | 5.42% | 2.78% | 2.56% | 3.28% | 2.94% | 0.75% | 2.50% |
| 20–24 y | 9.38% | 7.32% | 3.92% | 10.2% | 6.44% | 8.52% | 7.35% | 7.20% | 6.93% | 5.37% | 4.35% | 5.36% | 3.52% | 4.07% | 3.39% |
| 25–29 y | 11.3% | 11.5% | 7.26% | 8.06% | 9.38% | 9.80% | 8.30% | 6.28% | 7.07% | 6.30% | 7.85% | 7.08% | 5.56% | 5.02% | 5.54% |
| 30–34 y | 8.38% | 5.93% | 8.58% | 11.1% | 10.5% | 9.01% | 7.74% | 7.83% | 8.73% | 8.12% | 8.20% | 8.26% | 7.36% | 6.61% | 7.46% |
| 35–39 y | 11.3% | 11.3% | 9.36% | 9.25% | 7.50% | 8.41% | 10.0% | 7.70% | 9.52% | 7.86% | 7.54% | 7.57% | 7.55% | 6.81% | 7.89% |
| 40–44 y | 9.62% | 7.91% | 10.3% | 9.03% | 8.30% | 8.09% | 8.51% | 7.28% | 8.70% | 7.97% | 7.71% | 8.11% | 7.19% | 7.37% | 7.70% |
| 45–49 y | 11.1% | 8.91% | 11.7% | 8.74% | 9.59% | 10.1% | 7.92% | 8.25% | 8.21% | 7.48% | 8.23% | 7.98% | 7.10% | 6.77% | 8.19% |
| 50–54 y | 4.04% | 5.09% | 4.35% | 6.18% | 6.57% | 7.70% | 6.92% | 6.44% | 7.70% | 8.06% | 7.38% | 7.83% | 6.90% | 7.11% | 7.36% |
| 55–59 y | 5.19% | 5.45% | 5.68% | 3.77% | 5.41% | 5.23% | 4.74% | 4.92% | 5.17% | 5.39% | 5.64% | 5.74% | 5.68% | 5.47% | 6.42% |
| 60–64 y | 4.65% | 4.40% | 2.33% | 2.05% | 4.64% | 4.55% | 4.85% | 4.28% | 4.84% | 4.20% | 3.92% | 4.02% | 3.26% | 3.92% | 5.14% |
| 65–69 y | 3.81% | 7.62% | 1.71% | 3.57% | 2.44% | 2.59% | 3.18% | 3.35% | 3.44% | 3.61% | 3.21% | 4.00% | 2.99% | 3.56% | 3.16% |
| 70–74 y | 3.13% | 1.75% | 3.40% | 2.01% | 2.10% | 2.47% | 1.90% | 1.44% | 1.91% | 2.15% | 2.18% | 2.80% | 2.73% | 2.09% | 2.97% |
| Total | 5.84% | 5.80% | 4.82% | 4.72% | 5.12% | 5.33% | 5.17% | 4.71% | 5.30% | 5.06% | 5.02% | 5.39% | 4.81% | 4.76% | 5.46% |

Table S7. Proportion of those prescribed high-potency psychotropics among subscribers to the health insurance service who were prescribed psychotropic drugs (by 5-year age group and sex)

Hypnotics (male)

|  | 2005 | 2006 | 2007 | 2008 | 2009 | 2010 | 2011 | 2012 | 2013 | 2014 | 2015 | 2016 | 2017 | 2018 | 2019 |
| --- | --- | --- | --- | --- | --- | --- | --- | --- | --- | --- | --- | --- | --- | --- | --- |
| 0–4 y | 0.00% | 0.00% | 0.00% | 0.00% | 20.0% | 0.00% | 11.1% | 0.00% | 4.55% | 4.55% | 0.00% | 3.70% | 6.67% | 0.00% | 0.00% |
| 5–9 y | 0.00% | 0.00% | 0.00% | 0.00% | 0.00% | 0.00% | 0.00% | 0.00% | 6.98% | 10.3% | 1.61% | 4.35% | 3.85% | 10.9% | 11.4% |
| 10–14 y | 0.00% | 10.0% | 11.1% | 9.09% | 11.1% | 0.00% | 5.88% | 1.85% | 6.93% | 10.1% | 4.05% | 5.88% | 8.61% | 6.42% | 6.27% |
| 15–19 y | 11.8% | 4.55% | 13.3% | 20.0% | 10.0% | 9.20% | 13.7% | 15.0% | 12.0% | 11.4% | 10.4% | 12.2% | 10.7% | 8.37% | 11.0% |
| 20–24 y | 20.0% | 18.5% | 20.3% | 22.1% | 23.4% | 18.3% | 17.7% | 17.1% | 18.7% | 14.0% | 14.3% | 13.7% | 12.8% | 11.4% | 12.4% |
| 25–29 y | 17.0% | 21.4% | 21.9% | 20.0% | 24.8% | 25.5% | 19.5% | 20.2% | 17.7% | 17.2% | 16.9% | 18.2% | 16.9% | 15.3% | 16.4% |
| 30–34 y | 18.2% | 20.5% | 21.8% | 26.7% | 27.9% | 22.5% | 23.1% | 19.1% | 19.8% | 20.8% | 19.4% | 19.0% | 17.5% | 16.3% | 17.9% |
| 35–39 y | 21.8% | 25.5% | 22.4% | 26.9% | 25.7% | 23.1% | 23.0% | 20.8% | 20.9% | 21.4% | 20.6% | 20.8% | 18.1% | 17.4% | 19.0% |
| 40–44 y | 24.9% | 21.3% | 23.0% | 24.3% | 22.7% | 23.6% | 23.3% | 21.0% | 21.2% | 20.4% | 19.6% | 20.6% | 19.4% | 19.4% | 19.9% |
| 45–49 y | 23.4% | 20.4% | 20.0% | 20.7% | 19.2% | 19.6% | 22.2% | 19.1% | 19.6% | 20.1% | 18.3% | 19.8% | 18.7% | 18.8% | 19.8% |
| 50–54 y | 17.3% | 21.9% | 19.2% | 16.3% | 17.5% | 18.1% | 18.3% | 18.0% | 17.8% | 18.7% | 18.8% | 20.0% | 18.2% | 17.3% | 18.8% |
| 55–59 y | 14.6% | 13.2% | 12.7% | 9.97% | 13.8% | 16.5% | 14.0% | 14.6% | 15.5% | 15.8% | 15.9% | 16.5% | 15.2% | 15.3% | 16.7% |
| 60–64 y | 11.6% | 8.75% | 8.82% | 14.0% | 10.2% | 10.8% | 10.2% | 9.80% | 9.33% | 10.5% | 10.2% | 11.5% | 11.4% | 10.6% | 11.8% |
| 65–69 y | 3.85% | 20.0% | 0.00% | 5.66% | 13.3% | 11.4% | 12.2% | 9.61% | 9.38% | 9.43% | 9.04% | 9.01% | 7.56% | 6.83% | 8.31% |
| 70–74 y | 14.3% | 0.00% | 0.00% | 2.08% | 10.7% | 12.2% | 9.09% | 9.31% | 9.21% | 10.9% | 8.60% | 9.11% | 7.17% | 6.34% | 6.78% |
| Total | 13.8% | 14.1% | 13.7% | 13.1% | 15.9% | 15.9% | 15.1% | 14.1% | 14.1% | 14.8% | 13.9% | 14.6% | 13.3% | 12.7% | 13.9% |

Table S7. Proportion of those prescribed high-potency psychotropics among subscribers to the health insurance service who were prescribed psychotropic drugs (by 5-year age group and sex)

Hypnotics (female)

|  | 2005 | 2006 | 2007 | 2008 | 2009 | 2010 | 2011 | 2012 | 2013 | 2014 | 2015 | 2016 | 2017 | 2018 | 2019 |
| --- | --- | --- | --- | --- | --- | --- | --- | --- | --- | --- | --- | --- | --- | --- | --- |
| 0–4 y | 0.00% | 0.00% | 0.00% | 0.00% | 0.00% | 0.00% | 0.00% | 0.00% | 0.00% | 5.56% | 4.35% | 0.00% | 3.85% | 0.00% | 3.85% |
| 5–9 y | 0.00% | 0.00% | 0.00% | 0.00% | 0.00% | 0.00% | 12.5% | 0.00% | 0.00% | 3.03% | 2.13% | 4.17% | 6.56% | 8.77% | 6.41% |
| 10–14 y | 12.5% | 0.00% | 14.3% | 0.00% | 7.14% | 11.5% | 12.9% | 11.6% | 12.2% | 4.08% | 4.51% | 2.07% | 3.72% | 5.94% | 5.96% |
| 15–19 y | 0.00% | 14.7% | 19.5% | 17.7% | 18.8% | 21.4% | 14.4% | 15.7% | 12.6% | 7.82% | 9.62% | 10.0% | 12.5% | 10.7% | 11.0% |
| 20–24 y | 19.4% | 21.6% | 24.6% | 22.0% | 25.3% | 25.7% | 22.3% | 26.3% | 21.4% | 23.9% | 17.2% | 18.0% | 15.7% | 11.5% | 17.4% |
| 25–29 y | 24.0% | 19.1% | 22.7% | 18.4% | 25.8% | 23.0% | 17.9% | 19.4% | 21.2% | 20.5% | 20.1% | 20.8% | 16.8% | 18.5% | 19.2% |
| 30–34 y | 18.3% | 22.1% | 12.9% | 23.2% | 23.7% | 25.1% | 22.3% | 19.7% | 22.2% | 20.3% | 19.4% | 19.8% | 20.0% | 18.3% | 20.2% |
| 35–39 y | 18.5% | 21.3% | 19.2% | 23.0% | 21.0% | 22.4% | 22.2% | 22.9% | 23.3% | 20.5% | 19.7% | 19.7% | 19.8% | 19.4% | 20.0% |
| 40–44 y | 15.9% | 20.4% | 22.4% | 22.5% | 21.1% | 22.2% | 22.0% | 20.6% | 19.8% | 20.2% | 19.9% | 20.1% | 19.5% | 18.9% | 19.4% |
| 45–49 y | 21.1% | 19.8% | 22.4% | 21.4% | 22.7% | 21.6% | 17.9% | 18.3% | 19.3% | 19.9% | 18.0% | 18.5% | 17.4% | 17.4% | 18.9% |
| 50–54 y | 14.9% | 13.6% | 12.5% | 16.9% | 15.7% | 15.4% | 16.8% | 16.3% | 16.9% | 17.1% | 16.4% | 16.1% | 16.5% | 15.6% | 16.6% |
| 55–59 y | 10.8% | 9.04% | 12.2% | 12.3% | 12.7% | 14.1% | 12.5% | 11.9% | 12.3% | 13.2% | 12.3% | 13.2% | 13.2% | 13.4% | 14.6% |
| 60–64 y | 8.54% | 3.90% | 9.30% | 9.30% | 12.2% | 9.21% | 10.7% | 9.61% | 10.5% | 9.88% | 10.1% | 9.16% | 9.43% | 9.47% | 10.8% |
| 65–69 y | 9.78% | 8.08% | 3.64% | 8.07% | 6.86% | 8.48% | 10.5% | 7.46% | 8.03% | 9.46% | 9.26% | 8.77% | 7.07% | 6.33% | 7.85% |
| 70–74 y | 3.28% | 5.65% | 8.16% | 6.57% | 7.31% | 6.91% | 6.52% | 6.50% | 7.22% | 7.71% | 7.64% | 5.75% | 6.41% | 6.37% | 8.07% |
| Total | 11.1% | 9.76% | 10.4% | 11.6% | 12.4% | 12.2% | 12.2% | 11.3% | 11.9% | 12.3% | 11.9% | 11.4% | 11.1% | 10.8% | 12.5% |

Table S7. Proportion of those prescribed high-potency psychotropics among subscribers to the health insurance service who were prescribed psychotropic drugs (by 5-year age group and sex)

Antidepressants (male)

|  | 2005 | 2006 | 2007 | 2008 | 2009 | 2010 | 2011 | 2012 | 2013 | 2014 | 2015 | 2016 | 2017 | 2018 | 2019 |
| --- | --- | --- | --- | --- | --- | --- | --- | --- | --- | --- | --- | --- | --- | --- | --- |
| 0–4 y | 0.00% | 0.00% | 0.00% | 0.00% | 0.00% | 0.00% | 0.00% | 0.00% | 0.00% | 0.00% | 0.00% | 0.00% | 0.00% | 0.00% | 0.00% |
| 5–9 y | 0.00% | 0.00% | 0.00% | 0.00% | 0.00% | 0.00% | 0.00% | 0.00% | 0.00% | 0.00% | 0.00% | 0.00% | 0.00% | 0.00% | 0.00% |
| 10–14 y | 0.00% | 0.00% | 0.00% | 0.00% | 0.00% | 0.96% | 0.81% | 0.00% | 0.50% | 0.00% | 0.42% | 1.19% | 0.00% | 1.15% | 1.42% |
| 15–19 y | 4.17% | 3.70% | 0.00% | 0.00% | 1.49% | 0.80% | 3.95% | 4.69% | 2.92% | 2.68% | 4.46% | 4.70% | 3.07% | 2.64% | 3.63% |
| 20–24 y | 7.41% | 6.58% | 7.61% | 4.17% | 2.58% | 3.81% | 5.95% | 6.10% | 5.30% | 3.56% | 4.80% | 5.28% | 4.77% | 4.61% | 4.82% |
| 25–29 y | 4.14% | 3.72% | 5.45% | 6.32% | 3.20% | 3.37% | 6.90% | 6.77% | 7.64% | 6.95% | 6.41% | 4.88% | 5.85% | 6.31% | 7.18% |
| 30–34 y | 7.14% | 6.53% | 6.13% | 4.31% | 5.34% | 4.81% | 7.27% | 7.76% | 9.33% | 8.16% | 6.80% | 7.89% | 7.27% | 7.53% | 8.00% |
| 35–39 y | 3.73% | 5.23% | 4.95% | 7.64% | 6.14% | 6.00% | 7.75% | 8.74% | 8.53% | 8.61% | 8.72% | 8.78% | 8.64% | 7.27% | 8.04% |
| 40–44 y | 7.54% | 8.52% | 6.42% | 5.93% | 5.06% | 4.71% | 7.19% | 9.25% | 9.75% | 9.85% | 8.94% | 8.89% | 8.31% | 8.16% | 9.18% |
| 45–49 y | 5.21% | 7.39% | 6.12% | 3.11% | 4.83% | 4.64% | 7.88% | 7.96% | 7.99% | 8.36% | 8.40% | 9.25% | 8.89% | 8.63% | 9.32% |
| 50–54 y | 3.91% | 5.38% | 7.14% | 6.77% | 5.46% | 5.01% | 8.12% | 6.07% | 6.74% | 7.70% | 7.91% | 8.85% | 8.20% | 8.20% | 9.38% |
| 55–59 y | 3.81% | 3.73% | 2.05% | 2.20% | 3.02% | 3.79% | 6.55% | 6.96% | 7.25% | 6.84% | 6.83% | 7.35% | 7.50% | 7.28% | 8.10% |
| 60–64 y | 8.70% | 2.56% | 0.00% | 1.47% | 1.90% | 0.99% | 3.09% | 2.17% | 3.01% | 4.07% | 4.51% | 5.01% | 4.86% | 5.48% | 6.61% |
| 65–69 y | 0.00% | 0.00% | 20.0% | 0.00% | 0.00% | 2.50% | 0.00% | 0.77% | 3.16% | 1.89% | 2.84% | 3.03% | 4.35% | 5.26% | 5.88% |
| 70–74 y | 0.00% | 0.00% | 0.00% | 0.00% | 4.00% | 3.03% | 0.00% | 0.00% | 4.72% | 4.61% | 2.62% | 4.10% | 3.76% | 4.07% | 5.64% |
| Total | 4.62% | 4.98% | 5.64% | 4.22% | 4.15% | 4.12% | 6.10% | 6.40% | 7.20% | 7.13% | 6.92% | 7.38% | 7.18% | 7.09% | 7.96% |

Table S7. Proportion of those prescribed high-potency psychotropics among subscribers to the health insurance service who were prescribed psychotropic drugs (by 5-year age group and sex)

Antidepressants (female)

|  | 2005 | 2006 | 2007 | 2008 | 2009 | 2010 | 2011 | 2012 | 2013 | 2014 | 2015 | 2016 | 2017 | 2018 | 2019 |
| --- | --- | --- | --- | --- | --- | --- | --- | --- | --- | --- | --- | --- | --- | --- | --- |
| 0–4 y | 0.00% | 0.00% | 0.00% | 0.00% | 0.00% | 0.00% | 0.00% | 0.00% | 0.00% | 0.00% | 0.00% | 0.00% | 0.00% | 0.00% | 0.00% |
| 5–9 y | 0.00% | 0.00% | 0.00% | 0.00% | 0.00% | 0.00% | 0.00% | 0.00% | 0.00% | 0.00% | 0.00% | 0.00% | 0.00% | 0.00% | 0.00% |
| 10–14 y | 0.00% | 0.00% | 0.00% | 0.00% | 0.00% | 0.00% | 0.00% | 1.15% | 0.89% | 0.00% | 0.00% | 0.00% | 0.48% | 0.80% | 0.00% |
| 15–19 y | 0.00% | 0.00% | 0.00% | 0.00% | 0.00% | 1.33% | 1.06% | 3.24% | 2.73% | 2.19% | 2.23% | 1.56% | 1.90% | 2.69% | 1.95% |
| 20–24 y | 1.85% | 5.48% | 3.19% | 1.68% | 0.49% | 0.88% | 3.90% | 6.07% | 4.24% | 3.31% | 5.29% | 4.71% | 2.92% | 3.17% | 3.83% |
| 25–29 y | 2.41% | 2.75% | 3.20% | 4.29% | 2.29% | 2.32% | 6.07% | 5.38% | 5.14% | 5.40% | 4.61% | 5.43% | 4.02% | 4.11% | 5.63% |
| 30–34 y | 1.38% | 1.19% | 1.08% | 6.99% | 3.11% | 3.32% | 5.18% | 5.31% | 3.90% | 4.61% | 4.45% | 5.20% | 5.04% | 4.95% | 6.94% |
| 35–39 y | 2.79% | 3.15% | 4.48% | 3.53% | 3.39% | 2.10% | 5.10% | 4.68% | 5.30% | 5.92% | 4.50% | 5.24% | 4.84% | 4.19% | 5.63% |
| 40–44 y | 3.68% | 2.99% | 2.44% | 3.74% | 3.27% | 2.85% | 4.77% | 5.80% | 6.57% | 7.02% | 5.55% | 5.85% | 4.74% | 5.58% | 6.44% |
| 45–49 y | 1.05% | 2.88% | 1.55% | 4.78% | 4.40% | 3.73% | 5.38% | 4.16% | 4.98% | 5.47% | 6.34% | 6.14% | 5.40% | 5.97% | 7.37% |
| 50–54 y | 1.22% | 0.99% | 2.08% | 1.33% | 2.52% | 3.94% | 3.33% | 5.49% | 5.19% | 5.43% | 5.48% | 6.31% | 5.89% | 6.24% | 7.42% |
| 55–59 y | 3.26% | 4.04% | 4.35% | 2.61% | 2.00% | 2.79% | 2.92% | 2.75% | 3.34% | 4.05% | 3.85% | 5.31% | 5.58% | 5.88% | 7.49% |
| 60–64 y | 0.00% | 0.00% | 0.00% | 4.17% | 2.11% | 1.88% | 2.89% | 2.78% | 4.03% | 4.00% | 3.77% | 3.72% | 3.77% | 5.05% | 6.64% |
| 65–69 y | 2.86% | 3.03% | 0.00% | 1.52% | 3.13% | 4.44% | 5.18% | 2.61% | 3.40% | 3.05% | 3.90% | 4.69% | 4.65% | 4.83% | 6.10% |
| 70–74 y | 0.00% | 0.00% | 0.00% | 0.00% | 2.13% | 0.00% | 0.00% | 1.13% | 3.09% | 3.27% | 2.96% | 3.17% | 2.30% | 4.19% | 4.08% |
| Total | 1.82% | 2.10% | 1.61% | 2.71% | 2.64% | 2.52% | 3.73% | 3.75% | 4.30% | 4.52% | 4.44% | 4.92% | 4.44% | 5.02% | 6.11% |

Table S7. Proportion of those prescribed high-potency psychotropics among subscribers to the health insurance service who were prescribed psychotropic drugs (by 5-year age group and sex)

Antipsychotics (male)

|  | 2005 | 2006 | 2007 | 2008 | 2009 | 2010 | 2011 | 2012 | 2013 | 2014 | 2015 | 2016 | 2017 | 2018 | 2019 |
| --- | --- | --- | --- | --- | --- | --- | --- | --- | --- | --- | --- | --- | --- | --- | --- |
| 0–4 y | 0.00% | 0.00% | 0.00% | 0.00% | 0.00% | 0.00% | 0.00% | 0.00% | 0.00% | 3.45% | 0.00% | 0.00% | 0.00% | 0.00% | 0.00% |
| 5–9 y | 0.00% | 0.00% | 0.00% | 0.00% | 0.00% | 1.54% | 0.00% | 1.79% | 1.10% | 1.57% | 0.83% | 0.56% | 0.22% | 0.35% | 0.46% |
| 10–14 y | 5.88% | 6.67% | 0.00% | 0.00% | 2.44% | 3.06% | 2.22% | 4.12% | 4.09% | 5.32% | 4.05% | 3.81% | 3.05% | 3.11% | 3.44% |
| 15–19 y | 4.35% | 26.9% | 3.57% | 6.00% | 5.48% | 9.49% | 8.86% | 11.2% | 11.1% | 11.3% | 9.21% | 11.4% | 10.0% | 9.17% | 8.66% |
| 20–24 y | 26.2% | 23.8% | 28.6% | 25.4% | 24.8% | 14.4% | 15.7% | 16.2% | 18.4% | 18.4% | 15.6% | 16.4% | 16.4% | 16.5% | 16.1% |
| 25–29 y | 23.6% | 23.7% | 25.8% | 23.9% | 24.0% | 27.2% | 21.6% | 22.2% | 20.4% | 18.2% | 18.0% | 19.1% | 15.7% | 15.9% | 15.4% |
| 30–34 y | 17.2% | 15.8% | 14.8% | 17.7% | 18.4% | 18.6% | 18.9% | 17.8% | 20.5% | 17.2% | 17.5% | 14.9% | 14.7% | 12.5% | 14.7% |
| 35–39 y | 8.96% | 14.3% | 10.7% | 8.57% | 8.72% | 11.2% | 14.8% | 10.7% | 14.5% | 12.4% | 12.9% | 12.9% | 12.1% | 11.5% | 12.0% |
| 40–44 y | 11.5% | 17.7% | 13.4% | 10.9% | 10.5% | 9.29% | 12.2% | 13.0% | 11.2% | 11.1% | 11.0% | 10.8% | 8.79% | 7.88% | 8.38% |
| 45–49 y | 12.8% | 18.2% | 20.0% | 20.3% | 13.6% | 8.66% | 10.7% | 7.92% | 10.4% | 9.56% | 9.61% | 9.35% | 8.57% | 7.92% | 8.09% |
| 50–54 y | 9.68% | 17.1% | 3.45% | 6.38% | 8.11% | 15.9% | 12.6% | 12.6% | 10.5% | 8.06% | 8.90% | 9.23% | 7.91% | 7.37% | 7.96% |
| 55–59 y | 15.2% | 15.2% | 19.5% | 4.44% | 11.8% | 8.47% | 7.81% | 5.50% | 8.53% | 9.71% | 8.41% | 8.83% | 9.57% | 8.44% | 8.35% |
| 60–64 y | 0.00% | 0.00% | 0.00% | 0.00% | 2.44% | 5.56% | 12.0% | 9.45% | 7.65% | 7.77% | 8.88% | 8.52% | 6.14% | 7.39% | 7.73% |
| 65–69 y | 0.00% | 0.00% | 0.00% | 0.00% | 7.14% | 0.00% | 16.0% | 1.52% | 9.20% | 5.32% | 5.10% | 3.75% | 5.13% | 3.87% | 6.02% |
| 70–74 y | 0.00% | 0.00% | 0.00% | 0.00% | 7.69% | 5.88% | 4.76% | 9.68% | 8.11% | 6.59% | 6.50% | 8.46% | 7.62% | 6.42% | 7.89% |
| Total | 10.1% | 10.5% | 13.5% | 8.20% | 11.2% | 10.5% | 12.2% | 10.9% | 11.9% | 10.6% | 10.5% | 10.5% | 9.53% | 8.73% | 9.24% |

Table S7. Proportion of those prescribed high-potency psychotropics among subscribers to the health insurance service who were prescribed psychotropic drugs (by 5-year age group and sex)

Antipsychotics (female)

|  | 2005 | 2006 | 2007 | 2008 | 2009 | 2010 | 2011 | 2012 | 2013 | 2014 | 2015 | 2016 | 2017 | 2018 | 2019 |
| --- | --- | --- | --- | --- | --- | --- | --- | --- | --- | --- | --- | --- | --- | --- | --- |
| 0–4 y | 0.00% | 0.00% | 0.00% | 0.00% | 0.00% | 0.00% | 0.00% | 0.00% | 0.00% | 0.00% | 0.00% | 0.00% | 0.00% | 0.00% | 0.00% |
| 5–9 y | 0.00% | 0.00% | 0.00% | 0.00% | 0.00% | 0.00% | 0.00% | 0.00% | 2.04% | 0.00% | 0.00% | 0.00% | 0.00% | 0.63% | 0.00% |
| 10–14 y | 0.00% | 0.00% | 0.00% | 0.00% | 3.45% | 1.82% | 1.92% | 3.33% | 4.76% | 3.62% | 3.90% | 1.65% | 2.60% | 1.70% | 1.59% |
| 15–19 y | 16.0% | 15.8% | 18.8% | 20.9% | 10.0% | 15.5% | 7.48% | 9.81% | 11.3% | 8.62% | 8.90% | 7.88% | 7.24% | 6.03% | 5.47% |
| 20–24 y | 11.8% | 8.00% | 13.6% | 18.6% | 14.1% | 13.5% | 19.8% | 15.4% | 17.6% | 14.5% | 14.0% | 14.8% | 11.8% | 9.86% | 10.2% |
| 25–29 y | 12.0% | 11.8% | 16.0% | 13.3% | 12.4% | 18.6% | 18.3% | 15.8% | 17.4% | 15.8% | 15.7% | 15.1% | 14.2% | 12.3% | 11.8% |
| 30–34 y | 20.9% | 17.6% | 17.1% | 15.3% | 14.3% | 15.8% | 14.5% | 18.2% | 18.3% | 16.2% | 15.4% | 14.3% | 14.1% | 14.1% | 13.5% |
| 35–39 y | 24.1% | 19.3% | 14.7% | 19.7% | 17.1% | 13.7% | 17.7% | 18.9% | 17.8% | 17.8% | 16.2% | 16.3% | 14.7% | 13.3% | 13.3% |
| 40–44 y | 16.7% | 21.5% | 16.0% | 18.2% | 16.4% | 18.7% | 16.9% | 18.1% | 15.7% | 14.4% | 13.8% | 14.3% | 13.7% | 12.9% | 14.7% |
| 45–49 y | 28.6% | 24.1% | 21.2% | 17.5% | 18.0% | 20.0% | 15.5% | 16.1% | 16.0% | 13.6% | 13.1% | 13.7% | 14.0% | 12.6% | 13.0% |
| 50–54 y | 32.4% | 15.0% | 10.0% | 12.7% | 12.6% | 14.2% | 16.9% | 16.0% | 15.9% | 16.8% | 14.7% | 16.3% | 13.9% | 13.6% | 12.7% |
| 55–59 y | 15.6% | 24.3% | 25.7% | 22.8% | 18.5% | 16.5% | 16.1% | 15.0% | 15.1% | 14.9% | 13.9% | 14.3% | 14.2% | 11.9% | 13.0% |
| 60–64 y | 28.6% | 45.5% | 26.7% | 13.3% | 18.4% | 15.9% | 13.2% | 15.6% | 13.6% | 13.9% | 13.9% | 14.4% | 11.8% | 11.7% | 12.8% |
| 65–69 y | 7.14% | 27.8% | 14.3% | 4.55% | 9.52% | 11.4% | 10.8% | 7.69% | 12.9% | 10.1% | 12.1% | 11.5% | 12.4% | 10.3% | 12.8% |
| 70–74 y | 0.0% | 0.00% | 6.25% | 10.0% | 2.94% | 6.52% | 6.56% | 5.26% | 7.09% | 8.51% | 5.32% | 6.70% | 8.60% | 6.08% | 8.47% |
| Total | 18.0% | 20.2% | 16.1% | 14.4% | 13.4% | 14.4% | 14.1% | 14.0% | 14.5% | 13.7% | 12.9% | 13.1% | 12.6% | 11.3% | 12.0% |
