# Supplementary material for: Changes in psychotropic polypharmacy and high‐potency prescription following policy change: Findings from a large scale Japanese claims database
Source: Psychiatry Clin Neurosci. 2022 Jul 2;76(9):475–7. doi: 10.1111/pcn.13432 (PMC9546399; doi:10.1111/pcn.13432)
Supplement: Supplementary file 13 — Table S8 Monthly prescription rate of psychotropics (by 5‐year age group and sex). [file PCN-76-475-s010.docx]

Table S8. Monthly prescription rate of psychotropics (by 5-year age group and sex)

Anxiolytics (male)

|  | 2005 | 2006 | 2007 | 2008 | 2009 | 2010 | 2011 | 2012 | 2013 | 2014 | 2015 | 2016 | 2017 | 2018 | 2019 |
| --- | --- | --- | --- | --- | --- | --- | --- | --- | --- | --- | --- | --- | --- | --- | --- |
| 0–4 y | 0.02% | 0.04% | 0.02% | 0.02% | 0.02% | 0.03% | 0.03% | 0.02% | 0.03% | 0.02% | 0.02% | 0.02% | 0.02% | 0.02% | 0.02% |
| 5–9 y | 0.03% | 0.03% | 0.09% | 0.07% | 0.09% | 0.07% | 0.05% | 0.04% | 0.04% | 0.04% | 0.03% | 0.04% | 0.03% | 0.04% | 0.03% |
| 10–14 y | 0.09% | 0.08% | 0.12% | 0.09% | 0.11% | 0.14% | 0.13% | 0.11% | 0.11% | 0.10% | 0.10% | 0.11% | 0.10% | 0.08% | 0.10% |
| 15–19 y | 0.36% | 0.27% | 0.27% | 0.37% | 0.33% | 0.37% | 0.30% | 0.27% | 0.27% | 0.27% | 0.25% | 0.24% | 0.24% | 0.23% | 0.24% |
| 20–24 y | 0.64% | 0.67% | 0.66% | 0.68% | 0.59% | 0.64% | 0.58% | 0.58% | 0.56% | 0.55% | 0.54% | 0.50% | 0.48% | 0.49% | 0.48% |
| 25–29 y | 1.01% | 1.17% | 1.39% | 1.11% | 1.00% | 1.03% | 1.03% | 0.98% | 0.97% | 0.89% | 0.92% | 0.88% | 0.86% | 0.81% | 0.82% |
| 30–34 y | 1.45% | 1.69% | 1.71% | 1.59% | 1.42% | 1.41% | 1.50% | 1.36% | 1.29% | 1.19% | 1.25% | 1.16% | 1.17% | 1.15% | 1.11% |
| 35–39 y | 2.20% | 2.30% | 2.29% | 2.21% | 1.74% | 1.68% | 1.79% | 1.68% | 1.67% | 1.61% | 1.63% | 1.57% | 1.50% | 1.37% | 1.37% |
| 40–44 y | 2.25% | 2.39% | 2.49% | 2.59% | 2.31% | 2.32% | 2.36% | 2.16% | 2.09% | 1.93% | 1.93% | 1.85% | 1.88% | 1.82% | 1.81% |
| 45–49 y | 2.26% | 2.62% | 2.41% | 2.43% | 2.39% | 2.48% | 2.57% | 2.50% | 2.47% | 2.34% | 2.39% | 2.29% | 2.20% | 2.08% | 2.05% |
| 50–54 y | 2.37% | 2.12% | 2.56% | 2.52% | 2.32% | 2.40% | 2.61% | 2.56% | 2.61% | 2.54% | 2.60% | 2.52% | 2.47% | 2.41% | 2.36% |
| 55–59 y | 2.65% | 2.67% | 2.81% | 2.46% | 2.40% | 2.29% | 2.33% | 2.33% | 2.38% | 2.36% | 2.57% | 2.55% | 2.50% | 2.44% | 2.40% |
| 60–64 y | 3.21% | 3.26% | 2.78% | 2.62% | 2.10% | 2.19% | 2.18% | 2.00% | 2.05% | 1.86% | 2.02% | 1.97% | 1.96% | 1.92% | 1.92% |
| 65–69 y | 5.51% | 7.18% | 4.55% | 3.95% | 3.08% | 2.54% | 2.71% | 2.65% | 2.45% | 2.19% | 2.06% | 2.04% | 1.83% | 1.75% | 1.69% |
| 70–74 y | 2.35% | 2.91% | 5.05% | 5.65% | 4.12% | 4.71% | 4.87% | 3.99% | 3.53% | 3.38% | 3.13% | 2.94% | 2.76% | 2.43% | 2.37% |
| Total | 1.84% | 2.04% | 2.04% | 1.98% | 1.69% | 1.71% | 1.77% | 1.67% | 1.63% | 1.55% | 1.57% | 1.52% | 1.47% | 1.41% | 1.40% |

Supporting Table 4. Monthly prescription rate of psychotropics (by 5-year age group and sex)

Anxiolytics (female）

|  | 2005 | 2006 | 2007 | 2008 | 2009 | 2010 | 2011 | 2012 | 2013 | 2014 | 2015 | 2016 | 2017 | 2018 | 2019 |
| --- | --- | --- | --- | --- | --- | --- | --- | --- | --- | --- | --- | --- | --- | --- | --- |
| 0–4 y | 0.07% | 0.04% | 0.02% | 0.03% | 0.04% | 0.04% | 0.02% | 0.02% | 0.03% | 0.02% | 0.02% | 0.02% | 0.01% | 0.02% | 0.02% |
| 5–9 y | 0.07% | 0.06% | 0.03% | 0.03% | 0.05% | 0.05% | 0.05% | 0.05% | 0.04% | 0.04% | 0.03% | 0.04% | 0.03% | 0.03% | 0.03% |
| 10–14 y | 0.19% | 0.18% | 0.13% | 0.13% | 0.14% | 0.17% | 0.17% | 0.14% | 0.14% | 0.13% | 0.12% | 0.11% | 0.12% | 0.11% | 0.11% |
| 15–19 y | 0.68% | 0.73% | 0.93% | 0.64% | 0.47% | 0.57% | 0.48% | 0.52% | 0.44% | 0.44% | 0.41% | 0.41% | 0.37% | 0.40% | 0.41% |
| 20–24 y | 1.04% | 1.18% | 1.33% | 1.11% | 1.20% | 1.15% | 1.16% | 1.17% | 1.09% | 1.02% | 1.02% | 0.96% | 0.93% | 0.87% | 0.85% |
| 25–29 y | 1.54% | 1.49% | 1.58% | 1.66% | 1.59% | 1.58% | 1.53% | 1.50% | 1.56% | 1.42% | 1.52% | 1.33% | 1.35% | 1.32% | 1.35% |
| 30–34 y | 1.47% | 1.83% | 1.90% | 1.71% | 1.74% | 1.68% | 1.72% | 1.63% | 1.61% | 1.66% | 1.58% | 1.52% | 1.51% | 1.50% | 1.56% |
| 35–39 y | 1.92% | 2.15% | 2.16% | 2.12% | 2.03% | 2.11% | 2.07% | 2.06% | 1.97% | 1.88% | 1.82% | 1.82% | 1.77% | 1.78% | 1.84% |
| 40–44 y | 2.74% | 2.72% | 2.88% | 2.93% | 2.78% | 2.74% | 2.54% | 2.42% | 2.44% | 2.32% | 2.26% | 2.23% | 2.15% | 2.11% | 2.12% |
| 45–49 y | 2.85% | 2.98% | 3.25% | 3.20% | 3.41% | 3.02% | 3.19% | 3.05% | 2.82% | 2.79% | 2.69% | 2.63% | 2.52% | 2.44% | 2.49% |
| 50–54 y | 3.22% | 3.59% | 3.53% | 3.97% | 3.64% | 3.58% | 3.61% | 3.52% | 3.48% | 3.33% | 3.26% | 3.23% | 3.10% | 2.98% | 2.95% |
| 55–59 y | 4.78% | 5.16% | 4.79% | 4.36% | 3.92% | 3.89% | 3.88% | 3.80% | 3.72% | 3.51% | 3.37% | 3.47% | 3.34% | 3.19% | 3.18% |
| 60–64 y | 5.72% | 6.03% | 5.28% | 5.81% | 5.21% | 4.96% | 4.58% | 4.46% | 4.12% | 3.83% | 3.67% | 3.58% | 3.34% | 3.24% | 3.21% |
| 65–69 y | 9.47% | 9.51% | 10.3% | 7.71% | 7.09% | 7.13% | 6.82% | 6.45% | 5.97% | 5.46% | 4.90% | 4.73% | 4.36% | 4.00% | 3.75% |
| 70–74 y | 5.94% | 10.3% | 12.7% | 12.1% | 10.8% | 9.86% | 9.07% | 8.97% | 8.56% | 7.83% | 7.40% | 7.16% | 6.58% | 5.96% | 5.47% |
| Total | 2.96% | 3.41% | 3.62% | 3.42% | 3.19% | 3.11% | 3.00% | 2.94% | 2.83% | 2.68% | 2.58% | 2.52% | 2.39% | 2.28% | 2.24% |

Supporting Table 4. Monthly prescription rate of psychotropics (by 5-year age group and sex)

Hypnotics (male)

|  | 2005 | 2006 | 2007 | 2008 | 2009 | 2010 | 2011 | 2012 | 2013 | 2014 | 2015 | 2016 | 2017 | 2018 | 2019 |
| --- | --- | --- | --- | --- | --- | --- | --- | --- | --- | --- | --- | --- | --- | --- | --- |
| 0–4 y | 0.00% | 0.00% | 0.01% | 0.01% | 0.02% | 0.02% | 0.02% | 0.02% | 0.02% | 0.02% | 0.02% | 0.02% | 0.02% | 0.02% | 0.02% |
| 5–9 y | 0.03% | 0.03% | 0.01% | 0.01% | 0.02% | 0.02% | 0.04% | 0.03% | 0.05% | 0.04% | 0.05% | 0.05% | 0.06% | 0.06% | 0.07% |
| 10–14 y | 0.05% | 0.10% | 0.08% | 0.07% | 0.04% | 0.06% | 0.08% | 0.09% | 0.11% | 0.11% | 0.11% | 0.13% | 0.15% | 0.16% | 0.18% |
| 15–19 y | 0.17% | 0.20% | 0.24% | 0.22% | 0.22% | 0.23% | 0.24% | 0.23% | 0.24% | 0.23% | 0.24% | 0.23% | 0.24% | 0.26% | 0.27% |
| 20–24 y | 0.39% | 0.41% | 0.47% | 0.47% | 0.46% | 0.50% | 0.51% | 0.54% | 0.48% | 0.49% | 0.55% | 0.50% | 0.48% | 0.50% | 0.53% |
| 25–29 y | 0.78% | 0.86% | 0.96% | 0.90% | 0.78% | 0.84% | 0.89% | 0.88% | 0.91% | 0.88% | 0.95% | 0.95% | 0.93% | 0.92% | 0.95% |
| 30–34 y | 1.09% | 1.25% | 1.26% | 1.37% | 1.15% | 1.22% | 1.31% | 1.29% | 1.24% | 1.18% | 1.29% | 1.24% | 1.28% | 1.26% | 1.26% |
| 35–39 y | 1.44% | 1.50% | 1.69% | 1.71% | 1.55% | 1.56% | 1.70% | 1.71% | 1.70% | 1.62% | 1.70% | 1.64% | 1.64% | 1.59% | 1.57% |
| 40–44 y | 1.66% | 1.89% | 1.91% | 2.01% | 1.93% | 2.02% | 2.19% | 2.19% | 2.17% | 2.11% | 2.14% | 2.10% | 2.12% | 2.10% | 2.12% |
| 45–49 y | 1.89% | 2.18% | 2.30% | 2.24% | 2.19% | 2.49% | 2.57% | 2.60% | 2.73% | 2.61% | 2.76% | 2.66% | 2.62% | 2.58% | 2.57% |
| 50–54 y | 1.93% | 1.90% | 2.25% | 2.38% | 2.32% | 2.53% | 3.00% | 3.00% | 3.03% | 3.07% | 3.24% | 3.19% | 3.20% | 3.19% | 3.27% |
| 55–59 y | 2.63% | 2.90% | 2.61% | 2.48% | 2.57% | 2.69% | 2.83% | 2.89% | 3.08% | 3.21% | 3.42% | 3.64% | 3.56% | 3.58% | 3.65% |
| 60–64 y | 2.64% | 2.87% | 3.19% | 3.07% | 2.93% | 2.83% | 3.04% | 2.89% | 2.97% | 2.83% | 3.08% | 3.06% | 3.06% | 3.17% | 3.36% |
| 65–69 y | 7.16% | 4.14% | 4.55% | 4.76% | 5.38% | 4.39% | 5.38% | 5.04% | 4.35% | 3.96% | 3.73% | 3.75% | 3.76% | 3.65% | 3.41% |
| 70–74 y | 2.35% | 7.44% | 8.08% | 7.54% | 6.78% | 8.56% | 8.32% | 8.01% | 7.95% | 7.63% | 7.40% | 7.13% | 6.58% | 6.39% | 6.01% |
| Total | 1.66% | 1.86% | 2.01% | 2.00% | 1.97% | 2.07% | 2.24% | 2.21% | 2.21% | 2.17% | 2.23% | 2.20% | 2.18% | 2.18% | 2.18% |

Supporting Table 4. Monthly prescription rate of psychotropics (by 5-year age group and sex)

Hypnotics (female)

|  | 2005 | 2006 | 2007 | 2008 | 2009 | 2010 | 2011 | 2012 | 2013 | 2014 | 2015 | 2016 | 2017 | 2018 | 2019 |
| --- | --- | --- | --- | --- | --- | --- | --- | --- | --- | --- | --- | --- | --- | --- | --- |
| 0–4 y | 0.00% | 0.00% | 0.02% | 0.01% | 0.02% | 0.01% | 0.00% | 0.02% | 0.02% | 0.02% | 0.02% | 0.02% | 0.02% | 0.02% | 0.02% |
| 5–9 y | 0.01% | 0.04% | 0.01% | 0.04% | 0.02% | 0.02% | 0.02% | 0.01% | 0.03% | 0.04% | 0.04% | 0.04% | 0.04% | 0.04% | 0.05% |
| 10–14 y | 0.09% | 0.08% | 0.07% | 0.03% | 0.07% | 0.08% | 0.08% | 0.07% | 0.08% | 0.10% | 0.11% | 0.11% | 0.12% | 0.14% | 0.15% |
| 15–19 y | 0.42% | 0.39% | 0.45% | 0.41% | 0.35% | 0.39% | 0.35% | 0.36% | 0.32% | 0.33% | 0.32% | 0.34% | 0.34% | 0.35% | 0.39% |
| 20–24 y | 0.58% | 0.73% | 0.74% | 0.77% | 0.94% | 0.86% | 0.89% | 0.97% | 0.92% | 0.94% | 0.95% | 0.85% | 0.85% | 0.87% | 0.87% |
| 25–29 y | 1.00% | 1.11% | 1.12% | 1.02% | 1.19% | 1.23% | 1.16% | 1.28% | 1.29% | 1.25% | 1.45% | 1.35% | 1.42% | 1.40% | 1.50% |
| 30–34 y | 1.01% | 0.98% | 1.33% | 1.33% | 1.26% | 1.26% | 1.26% | 1.18% | 1.32% | 1.37% | 1.46% | 1.43% | 1.43% | 1.45% | 1.57% |
| 35–39 y | 1.21% | 1.29% | 1.31% | 1.33% | 1.40% | 1.43% | 1.47% | 1.53% | 1.53% | 1.51% | 1.51% | 1.56% | 1.60% | 1.65% | 1.70% |
| 40–44 y | 1.53% | 1.49% | 1.70% | 1.82% | 1.94% | 1.92% | 1.84% | 1.93% | 1.89% | 1.88% | 1.93% | 1.99% | 1.95% | 1.96% | 2.03% |
| 45–49 y | 1.72% | 1.78% | 2.07% | 2.17% | 2.39% | 2.35% | 2.43% | 2.43% | 2.48% | 2.36% | 2.42% | 2.38% | 2.35% | 2.37% | 2.48% |
| 50–54 y | 2.18% | 1.96% | 2.45% | 2.56% | 3.01% | 2.85% | 2.90% | 3.15% | 3.17% | 3.21% | 3.21% | 3.17% | 3.06% | 3.09% | 3.09% |
| 55–59 y | 3.45% | 3.53% | 3.12% | 2.96% | 3.38% | 3.42% | 3.37% | 3.79% | 3.89% | 3.73% | 3.80% | 3.82% | 3.81% | 3.88% | 3.91% |
| 60–64 y | 5.46% | 5.11% | 5.28% | 5.13% | 5.16% | 5.23% | 4.81% | 5.12% | 4.85% | 4.56% | 4.44% | 4.45% | 4.29% | 4.31% | 4.46% |
| 65–69 y | 8.30% | 8.97% | 9.68% | 8.86% | 7.99% | 8.24% | 8.27% | 8.69% | 8.16% | 7.33% | 7.06% | 6.85% | 6.37% | 6.32% | 6.04% |
| 70–74 y | 5.66% | 11.2% | 12.7% | 13.0% | 12.9% | 13.3% | 12.9% | 12.9% | 12.6% | 11.7% | 12.2% | 11.6% | 11.1% | 10.6% | 9.62% |
| Total | 2.31% | 2.70% | 2.97% | 2.95% | 3.03% | 3.10% | 3.05% | 3.21% | 3.18% | 3.05% | 3.13% | 3.05% | 2.96% | 2.97% | 2.93% |

Supporting Table 4. Monthly prescription rate of psychotropics (by 5-year age group and sex)

Antidepressants (male)

|  | 2005 | 2006 | 2007 | 2008 | 2009 | 2010 | 2011 | 2012 | 2013 | 2014 | 2015 | 2016 | 2017 | 2018 | 2019 |
| --- | --- | --- | --- | --- | --- | --- | --- | --- | --- | --- | --- | --- | --- | --- | --- |
| 0–4 y | 0.00% | 0.00% | 0.00% | 0.01% | 0.00% | 0.00% | 0.00% | 0.00% | 0.00% | 0.00% | 0.00% | 0.00% | 0.00% | 0.00% | 0.00% |
| 5–9 y | 0.13% | 0.14% | 0.17% | 0.22% | 0.16% | 0.17% | 0.14% | 0.12% | 0.12% | 0.09% | 0.10% | 0.07% | 0.07% | 0.07% | 0.07% |
| 10–14 y | 0.27% | 0.21% | 0.18% | 0.20% | 0.27% | 0.29% | 0.29% | 0.26% | 0.21% | 0.21% | 0.18% | 0.17% | 0.17% | 0.16% | 0.17% |
| 15–19 y | 0.24% | 0.24% | 0.26% | 0.30% | 0.30% | 0.34% | 0.35% | 0.34% | 0.32% | 0.33% | 0.29% | 0.32% | 0.32% | 0.31% | 0.34% |
| 20–24 y | 0.47% | 0.58% | 0.63% | 0.59% | 0.57% | 0.66% | 0.64% | 0.59% | 0.59% | 0.59% | 0.68% | 0.62% | 0.61% | 0.66% | 0.70% |
| 25–29 y | 1.06% | 1.28% | 1.40% | 1.20% | 1.17% | 1.12% | 1.26% | 1.20% | 1.19% | 1.14% | 1.18% | 1.15% | 1.14% | 1.13% | 1.20% |
| 30–34 y | 1.55% | 1.80% | 2.02% | 1.94% | 1.59% | 1.58% | 1.70% | 1.63% | 1.53% | 1.48% | 1.58% | 1.52% | 1.57% | 1.56% | 1.53% |
| 35–39 y | 2.11% | 2.40% | 2.62% | 2.56% | 2.02% | 1.97% | 2.06% | 2.07% | 2.00% | 1.96% | 1.96% | 1.94% | 1.92% | 1.84% | 1.86% |
| 40–44 y | 1.97% | 2.22% | 2.61% | 2.70% | 2.43% | 2.49% | 2.59% | 2.41% | 2.35% | 2.30% | 2.34% | 2.26% | 2.29% | 2.30% | 2.35% |
| 45–49 y | 1.84% | 2.34% | 2.28% | 2.33% | 2.23% | 2.54% | 2.63% | 2.68% | 2.66% | 2.63% | 2.75% | 2.63% | 2.64% | 2.57% | 2.59% |
| 50–54 y | 1.47% | 1.46% | 2.02% | 2.19% | 1.97% | 1.96% | 2.20% | 2.39% | 2.42% | 2.48% | 2.66% | 2.59% | 2.75% | 2.79% | 2.94% |
| 55–59 y | 1.30% | 1.55% | 1.67% | 1.45% | 1.49% | 1.57% | 1.64% | 1.71% | 1.80% | 1.96% | 2.21% | 2.26% | 2.35% | 2.45% | 2.62% |
| 60–64 y | 0.88% | 1.40% | 1.03% | 1.12% | 0.85% | 0.91% | 1.00% | 0.95% | 0.95% | 1.05% | 1.18% | 1.26% | 1.37% | 1.43% | 1.60% |
| 65–69 y | 2.20% | 1.38% | 1.34% | 1.17% | 1.25% | 1.00% | 0.96% | 1.03% | 0.92% | 0.92% | 0.97% | 0.94% | 0.94% | 0.97% | 1.07% |
| 70–74 y | 0.34% | 0.97% | 1.68% | 2.04% | 2.02% | 1.92% | 1.64% | 1.46% | 1.41% | 1.51% | 1.32% | 1.33% | 1.30% | 1.38% | 1.39% |
| Total | 1.13% | 1.30% | 1.44% | 1.44% | 1.31% | 1.32% | 1.38% | 1.37% | 1.35% | 1.36% | 1.42% | 1.40% | 1.43% | 1.45% | 1.52% |

Supporting Table 4. Monthly prescription rate of psychotropics (by 5-year age group and sex)

Antidepressants (female)

|  | 2005 | 2006 | 2007 | 2008 | 2009 | 2010 | 2011 | 2012 | 2013 | 2014 | 2015 | 2016 | 2017 | 2018 | 2019 |
| --- | --- | --- | --- | --- | --- | --- | --- | --- | --- | --- | --- | --- | --- | --- | --- |
| 0–4 y | 0.00% | 0.00% | 0.00% | 0.00% | 0.00% | 0.00% | 0.00% | 0.00% | 0.00% | 0.00% | 0.00% | 0.00% | 0.00% | 0.00% | 0.00% |
| 5–9 y | 0.11% | 0.05% | 0.06% | 0.09% | 0.08% | 0.04% | 0.06% | 0.05% | 0.07% | 0.04% | 0.04% | 0.04% | 0.03% | 0.04% | 0.04% |
| 10–14 y | 0.17% | 0.12% | 0.09% | 0.09% | 0.15% | 0.12% | 0.13% | 0.15% | 0.13% | 0.13% | 0.14% | 0.12% | 0.13% | 0.16% | 0.15% |
| 15–19 y | 0.53% | 0.49% | 0.70% | 0.65% | 0.48% | 0.50% | 0.53% | 0.51% | 0.44% | 0.41% | 0.44% | 0.40% | 0.41% | 0.45% | 0.53% |
| 20–24 y | 0.87% | 1.05% | 1.22% | 1.12% | 1.23% | 1.24% | 1.17% | 1.19% | 1.07% | 1.04% | 1.02% | 0.98% | 1.02% | 1.02% | 1.11% |
| 25–29 y | 1.11% | 1.44% | 1.59% | 1.46% | 1.44% | 1.49% | 1.49% | 1.44% | 1.39% | 1.36% | 1.55% | 1.41% | 1.44% | 1.51% | 1.68% |
| 30–34 y | 1.12% | 1.30% | 1.51% | 1.44% | 1.51% | 1.49% | 1.52% | 1.42% | 1.42% | 1.45% | 1.47% | 1.47% | 1.52% | 1.55% | 1.70% |
| 35–39 y | 1.43% | 1.69% | 1.61% | 1.76% | 1.69% | 1.65% | 1.66% | 1.67% | 1.65% | 1.61% | 1.65% | 1.61% | 1.67% | 1.74% | 1.86% |
| 40–44 y | 1.43% | 1.64% | 1.90% | 2.02% | 1.95% | 2.05% | 1.97% | 1.92% | 1.87% | 1.85% | 1.91% | 1.88% | 1.87% | 1.95% | 2.02% |
| 45–49 y | 1.50% | 1.53% | 1.76% | 1.83% | 2.07% | 1.98% | 2.12% | 2.07% | 2.08% | 2.07% | 2.11% | 2.09% | 2.07% | 2.11% | 2.26% |
| 50–54 y | 1.33% | 1.68% | 1.64% | 1.75% | 1.63% | 1.87% | 2.00% | 2.14% | 2.19% | 2.15% | 2.15% | 2.28% | 2.35% | 2.37% | 2.46% |
| 55–59 y | 1.90% | 1.86% | 1.67% | 1.80% | 1.77% | 1.71% | 1.73% | 1.89% | 1.95% | 2.00% | 2.06% | 2.08% | 2.17% | 2.33% | 2.48% |
| 60–64 y | 2.26% | 2.39% | 2.52% | 2.15% | 1.91% | 2.00% | 2.01% | 2.05% | 1.97% | 1.83% | 1.82% | 1.87% | 1.88% | 1.97% | 2.16% |
| 65–69 y | 3.16% | 2.99% | 3.70% | 3.63% | 2.77% | 2.77% | 3.22% | 2.77% | 2.45% | 2.42% | 2.34% | 2.22% | 2.14% | 2.08% | 2.19% |
| 70–74 y | 1.95% | 3.51% | 3.71% | 4.08% | 3.54% | 3.41% | 3.61% | 3.43% | 3.82% | 3.60% | 3.14% | 3.26% | 3.07% | 3.22% | 3.24% |
| Total | 1.35% | 1.55% | 1.68% | 1.71% | 1.60% | 1.62% | 1.68% | 1.66% | 1.65% | 1.62% | 1.62% | 1.61% | 1.62% | 1.67% | 1.78% |

Supporting Table 4. Monthly prescription rate of psychotropics (by 5-year age group and sex)

Antipsychotics (male)

|  | 2005 | 2006 | 2007 | 2008 | 2009 | 2010 | 2011 | 2012 | 2013 | 2014 | 2015 | 2016 | 2017 | 2018 | 2019 |
| --- | --- | --- | --- | --- | --- | --- | --- | --- | --- | --- | --- | --- | --- | --- | --- |
| 0–4 y | 0.03% | 0.02% | 0.02% | 0.02% | 0.01% | 0.01% | 0.01% | 0.02% | 0.02% | 0.03% | 0.02% | 0.02% | 0.02% | 0.03% | 0.03% |
| 5–9 y | 0.06% | 0.08% | 0.09% | 0.10% | 0.13% | 0.18% | 0.16% | 0.19% | 0.20% | 0.20% | 0.19% | 0.25% | 0.28% | 0.35% | 0.40% |
| 10–14 y | 0.17% | 0.14% | 0.16% | 0.21% | 0.19% | 0.27% | 0.31% | 0.31% | 0.36% | 0.40% | 0.38% | 0.43% | 0.52% | 0.59% | 0.65% |
| 15–19 y | 0.23% | 0.23% | 0.23% | 0.31% | 0.32% | 0.37% | 0.37% | 0.43% | 0.41% | 0.45% | 0.45% | 0.43% | 0.47% | 0.49% | 0.54% |
| 20–24 y | 0.37% | 0.32% | 0.29% | 0.35% | 0.40% | 0.41% | 0.44% | 0.46% | 0.47% | 0.47% | 0.56% | 0.55% | 0.54% | 0.56% | 0.57% |
| 25–29 y | 0.40% | 0.40% | 0.42% | 0.42% | 0.42% | 0.42% | 0.49% | 0.50% | 0.52% | 0.57% | 0.60% | 0.61% | 0.62% | 0.60% | 0.66% |
| 30–34 y | 0.32% | 0.41% | 0.47% | 0.45% | 0.44% | 0.48% | 0.52% | 0.55% | 0.54% | 0.56% | 0.66% | 0.65% | 0.65% | 0.66% | 0.68% |
| 35–39 y | 0.38% | 0.34% | 0.45% | 0.44% | 0.42% | 0.45% | 0.53% | 0.54% | 0.57% | 0.60% | 0.69% | 0.66% | 0.67% | 0.66% | 0.69% |
| 40–44 y | 0.39% | 0.38% | 0.47% | 0.44% | 0.50% | 0.49% | 0.58% | 0.59% | 0.61% | 0.65% | 0.69% | 0.68% | 0.71% | 0.73% | 0.75% |
| 45–49 y | 0.45% | 0.40% | 0.49% | 0.36% | 0.39% | 0.49% | 0.53% | 0.58% | 0.60% | 0.62% | 0.74% | 0.74% | 0.75% | 0.79% | 0.82% |
| 50–54 y | 0.36% | 0.39% | 0.32% | 0.39% | 0.36% | 0.42% | 0.52% | 0.51% | 0.55% | 0.63% | 0.74% | 0.74% | 0.76% | 0.81% | 0.83% |
| 55–59 y | 0.41% | 0.38% | 0.47% | 0.36% | 0.34% | 0.39% | 0.35% | 0.37% | 0.45% | 0.48% | 0.61% | 0.65% | 0.68% | 0.73% | 0.76% |
| 60–64 y | 0.31% | 0.32% | 0.28% | 0.54% | 0.33% | 0.32% | 0.31% | 0.29% | 0.27% | 0.28% | 0.39% | 0.40% | 0.41% | 0.42% | 0.46% |
| 65–69 y | 0.83% | 1.66% | 0.27% | 0.72% | 0.50% | 0.35% | 0.45% | 0.52% | 0.42% | 0.41% | 0.39% | 0.35% | 0.30% | 0.36% | 0.32% |
| 70–74 y | 0.67% | 0.97% | 0.00% | 1.10% | 1.05% | 0.99% | 0.88% | 1.00% | 0.82% | 0.90% | 0.85% | 0.89% | 0.64% | 0.61% | 0.60% |
| Total | 0.36% | 0.43% | 0.32% | 0.42% | 0.40% | 0.41% | 0.44% | 0.47% | 0.47% | 0.50% | 0.55% | 0.56% | 0.56% | 0.58% | 0.61% |

Supporting Table 4. Monthly prescription rate of psychotropics (by 5-year age group and sex)

Antipsychotics (female)

|  | 2005 | 2006 | 2007 | 2008 | 2009 | 2010 | 2011 | 2012 | 2013 | 2014 | 2015 | 2016 | 2017 | 2018 | 2019 |
| --- | --- | --- | --- | --- | --- | --- | --- | --- | --- | --- | --- | --- | --- | --- | --- |
| 0–4 y | 0.01% | 0.00% | 0.03% | 0.02% | 0.01% | 0.01% | 0.01% | 0.00% | 0.00% | 0.01% | 0.01% | 0.01% | 0.01% | 0.01% | 0.01% |
| 5–9 y | 0.05% | 0.05% | 0.07% | 0.05% | 0.07% | 0.05% | 0.04% | 0.06% | 0.06% | 0.06% | 0.07% | 0.08% | 0.08% | 0.10% | 0.11% |
| 10–14 y | 0.12% | 0.09% | 0.11% | 0.06% | 0.14% | 0.16% | 0.13% | 0.15% | 0.14% | 0.15% | 0.17% | 0.18% | 0.22% | 0.23% | 0.29% |
| 15–19 y | 0.31% | 0.44% | 0.35% | 0.34% | 0.33% | 0.34% | 0.41% | 0.40% | 0.45% | 0.43% | 0.39% | 0.41% | 0.42% | 0.46% | 0.49% |
| 20–24 y | 0.55% | 0.36% | 0.57% | 0.66% | 0.76% | 0.75% | 0.74% | 0.76% | 0.74% | 0.74% | 0.83% | 0.79% | 0.74% | 0.76% | 0.79% |
| 25–29 y | 0.67% | 0.67% | 0.64% | 0.54% | 0.62% | 0.66% | 0.67% | 0.80% | 0.82% | 0.86% | 1.00% | 0.95% | 0.94% | 0.96% | 1.04% |
| 30–34 y | 0.52% | 0.57% | 0.62% | 0.74% | 0.66% | 0.66% | 0.63% | 0.66% | 0.74% | 0.78% | 0.85% | 0.82% | 0.84% | 0.90% | 0.98% |
| 35–39 y | 0.70% | 0.63% | 0.74% | 0.63% | 0.61% | 0.70% | 0.73% | 0.77% | 0.78% | 0.80% | 0.83% | 0.80% | 0.80% | 0.84% | 0.89% |
| 40–44 y | 0.76% | 0.77% | 0.87% | 0.90% | 0.75% | 0.76% | 0.82% | 0.85% | 0.84% | 0.88% | 0.88% | 0.91% | 0.90% | 0.92% | 0.95% |
| 45–49 y | 0.66% | 0.85% | 0.90% | 0.85% | 0.95% | 0.93% | 0.92% | 0.94% | 0.94% | 0.97% | 0.97% | 0.97% | 0.98% | 1.03% | 1.10% |
| 50–54 y | 0.60% | 0.66% | 0.68% | 0.74% | 0.81% | 0.82% | 0.95% | 0.99% | 0.99% | 1.04% | 1.04% | 1.02% | 1.05% | 1.09% | 1.12% |
| 55–59 y | 0.66% | 0.69% | 0.64% | 0.67% | 0.76% | 0.76% | 0.74% | 0.75% | 0.85% | 0.89% | 0.92% | 0.98% | 1.02% | 1.09% | 1.14% |
| 60–64 y | 0.93% | 0.73% | 0.92% | 0.89% | 0.66% | 0.66% | 0.64% | 0.75% | 0.74% | 0.79% | 0.76% | 0.77% | 0.81% | 0.83% | 0.91% |
| 65–69 y | 1.26% | 1.63% | 1.23% | 1.21% | 0.61% | 0.90% | 1.08% | 0.94% | 0.86% | 0.78% | 0.83% | 0.86% | 0.84% | 0.83% | 0.86% |
| 70–74 y | 0.46% | 1.08% | 1.38% | 1.22% | 1.28% | 1.25% | 1.31% | 1.23% | 1.15% | 1.18% | 1.17% | 1.18% | 1.01% | 0.94% | 0.96% |
| Total | 0.59% | 0.65% | 0.69% | 0.68% | 0.64% | 0.67% | 0.70% | 0.72% | 0.72% | 0.74% | 0.77% | 0.77% | 0.77% | 0.79% | 0.84% |
